# Characterization and engineering of the biosynthesis gene cluster for antitumor macrolides PM100117 and PM100118 from a marine actinobacteria: generation of a novel improved derivative

**DOI:** 10.1186/s12934-016-0443-5

**Published:** 2016-02-22

**Authors:** Raúl García Salcedo, Carlos Olano, Cristina Gómez, Rogelio Fernández, Alfredo F. Braña, Carmen Méndez, Fernando de la Calle, José A. Salas

**Affiliations:** Departamento de Biología Funcional e Instituto Universitario de Oncología del Principado de Asturias (I.U.O.P.A), Universidad de Oviedo, 33006 Oviedo, Asturias Spain; Drug Discovery Area, PharmaMar SA, Avda. de los Reyes 1, Colmenar Viejo, 28770 Madrid, Spain

**Keywords:** Polyketide, Antitumor, Modular polyketide syntase, Deoxysugar, Glycosyltranferase, Structural analogue

## Abstract

**Background:**

PM100117 and PM100118 are glycosylated polyketides with remarkable antitumor activity, which derive from the marine symbiotic actinobacteria *Streptomyces caniferus* GUA-06-05-006A. Structurally, PM100117 and PM100118 are composed of a macrocyclic lactone, three deoxysugar units and a naphthoquinone (NQ) chromophore that shows a clear structural similarity to menaquinone.

**Results:**

Whole-genome sequencing of *S. caniferus* GUA-06-05-006A has enabled the identification of PM100117 and PM100118 biosynthesis gene cluster, which has been characterized on the basis of bioinformatics and genetic engineering data. The product of four genes shows high identity to proteins involved in the biosynthesis of menaquinone via futalosine. Deletion of one of these genes led to a decay in PM100117 and PM100118 production, and to the accumulation of several derivatives lacking NQ. Likewise, five additional genes have been genetically characterized to be involved in the biosynthesis of this moiety. Moreover, the generation of a mutant in a gene coding for a putative cytochrome P450 has led to the production of PM100117 and PM100118 structural analogues showing an enhanced in vitro cytotoxic activity relative to the parental products.

**Conclusions:**

Although a number of compounds structurally related to PM100117 and PM100118 has been discovered, this is, to our knowledge, the first insight reported into their biosynthesis. The structural resemblance of the NQ moiety to menaquinone, and the presence in the cluster of four putative menaquinone biosynthetic genes, suggests a connection between the biosynthesis pathways of both compounds. The availability of the PM100117 and PM100118 biosynthetic gene cluster will surely pave a way to the combinatorial engineering of more derivatives.

**Electronic supplementary material:**

The online version of this article (doi:10.1186/s12934-016-0443-5) contains supplementary material, which is available to authorized users.

## Background

Actinobacteria is an extensive phyla within the domain bacteria with a wide distribution in nature, encompassing both terrestrial and aquatic environments [1, 2]. Numerous actinobacteria species have an outstanding medical value as producers of cancer chemotherapeutic drugs [3], among other biologically active compounds [4, 5]. Terrestrial actinobacteria are the source of the vast majority of natural antitumor agents discovered up to date, many of which are the base of currently available chemotherapeutic treatments or are in advance clinical trials [6]. However, in spite of the enormous potential of soil actinobacteria as producers of antitumor agents, the rate of discovery of new antitumor drugs and other bioactive compounds from the terrestrial environment has nowadays decayed [7–9]. This, together with the worldwide-rising occurrence of cancer and the appearance of multi-drug resistant tumor cell lines, urges to extend the screening of new and improved chemotherapeutic products to less explored environments.

Ocean is a major component of the biosphere and an example of unexploited habitat with the potential to host the chemical and biological diversity required for the discovery of novel anticancer agents. A staggering diversity of actinobacteria species has been isolated from different marine substrates such as sediments seawater, seaweeds or mangroves [2]. Furthermore, tissues of mollusks and invertebrates are the niche of remarkably diverse symbiotic actinobacteria populations many of which could have lost the ability to grow independently of their host [10]. Some of the symbiotic functions attributable to symbiotic actinobacteria entail the production of bioactive secondary metabolites (e.g., for host defense) with potential pharmaceutical significance. Indeed, many natural products isolated from diverse marine invertebrates have been proven to be produced by their symbiotic microorganisms. That is the case, for example, of the dibenzodiazepinone diazepinomicin [11], the thiodepsipeptide thiocoraline [12] and the polyketide bryostatin [13], three potent antitumor drug leads; or the tetrahydroisoquinoline ecteinascidin 743 [14], an anticancer drug in current clinical use.

Although the screening of antitumor natural products from yet unexploited environments is a valid plan to alleviate the problem of the apparent chemical exhaustion of terrestrial sources, the use of metabolic engineering and combinatorial biosynthesis strategies intended to generate novel analogues from known natural agents [15–17], can be a no less interesting approach. However, application of these genetic engineering strategies requires to a certain extent detailed knowledge on the genetic and biochemical basis of the biosynthesis of relevant natural metabolites. Hence, the identification and characterization of biosynthetic gene clusters is not only an invaluable tool for the elucidation of the biosynthesis pathway of bioactive natural agents, but also an essential requirement for tackling the combinatorial engineering of novel analogues. In parallel with the development of these genetic engineering approaches, advances in next-generation whole-genome sequencing techniques [18] has favored the availability of a growing number of genomes from actinobacteria producing clinical attractive bioactive compounds. Mining of these genomes has enabled the identification and analysis of an exponentially increasing number of clusters [19], setting the stage for subsequent combinatorial engineering of novel derivatives. Furthermore, genome analysis by enhanced bioinformatics platforms associated to database [20–24] allows for disclosing the chemical diversity potentially hosted in theses actinobacteria, contributing to widen the availability of natural products by activation of cryptic clusters [19].

**Fig. 1:**
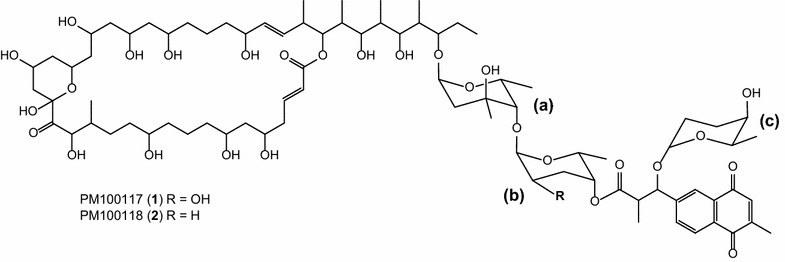
Chemical structures of PM100117 and PM100118. Deoxysugar moieties are indicated: (*a*) l-axenose, (*b*) l-rhodinose (R=H) or l-2-deoxi-fucose (R=OH), (*c*) l-rhodinose

In a recent publication [25], the discovery of PM100117 and PM100118 was reported. These glycosylated polyketide compounds, with remarkable antitumor activity, are produced by *Streptomyces caniferus* GUA-06-05-006A, a symbiotic actinobacteria isolated from the marine sponge *Filograna* sp. The structures of PM100117 and PM100118, consisting of a macrocyclic lactone, three deoxysugars and a 1, 4-naphthoquinone chromophore (Fig. 1), share a clear similarity with other antitumor polyketide compounds, including langkolide [26] and the promising anticancer drug lead GT35 [27]. Given their attractive as potential anticancer clinical drugs, PM100117 and PM100118 represent interesting targets for the combinatorial engineering of novel derivatives. The goals of this work were the identification and characterization of the PM100117 and PM100118 biosynthesis gene cluster, and engineering the gene cluster to generate novel derivatives with improved biological activity.

## Results

### Sequencing and bioinformatics analysis of *S. caniferus* GUA-06-05-006A genome

The first step to accomplish the identification of the PM100117 and PM100118 biosynthesis gene cluster was sequencing the *S. caniferus* GUA-06-05-006A chromosome. Whole-genome sequencing of this strain generated a total number of 548,579 paired-end reads with an average length of 372.5 nucleotides, producing a total sequence of 204.3 Mb. This represents a 20-fold coverage of the chromosome sequence, which is estimated in 9.8 Mb. De novo assembly of those sequences resulted in 907 contigs. Median (N50) of the contig assembly was 20.9 Kb, and the largest was around 119.3 Kb. Subsequent contig arrangement rinsed 33 scaffolds (mean: 276.2 Kb; N50: 1.5 Mb) generating a draft genome of 9.1 Mb with a G + C content of 70.63 %. *In silico* genome analyses with the antibiotics and Secondary Metabolite Analysis Shell (antiSMASH) algorithm [22] revealed the presence of 8582 open reading frames (ORFs), having at least 5045 proteins an assigned putative function. Sequence analyses with antiSMASH also showed the presence of 32 gene clusters potentially involved in the biosynthesis of secondary metabolites. Seven of these clusters were identified as containing gene sequences belonging to the type I (four clusters), II (two clusters) and III (one cluster) family of polyketide synthases (PKS). Likewise, genome sequence analysis detected nine additional gene clusters comprising modular enzyme-coding genes such as non-ribosomal peptide synthetase (NRPS, seven clusters) and hybrid PKSI-NRPS genes (two clusters). Other products from these gene clusters include one nucleoside, five terpenes and three butyrolactones, as well as compounds with a peptidic backbone such as three siderophores, three lantipeptides and one ectoine.

### Identification of PM100117 and PM100118 biosynthetic gene cluster

**Fig. 2:**
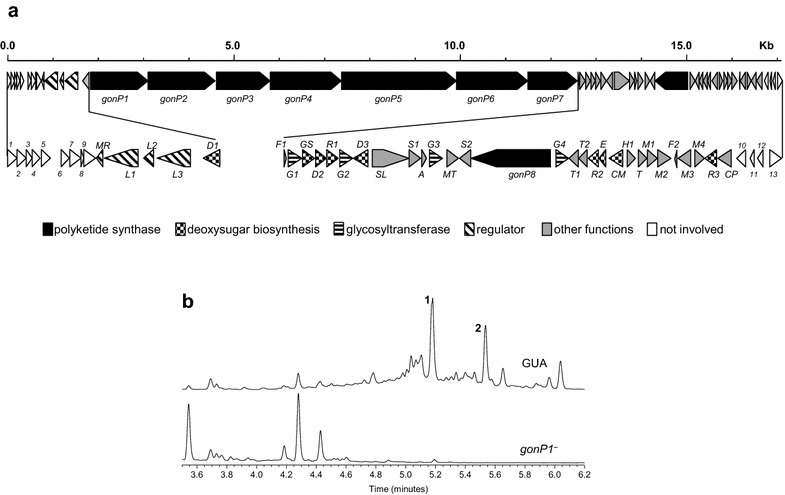
Identification and organization of the PM100117 and PM100118 biosynthetic gene cluster. **a** Organization of the PM100117 and PM100118 gene cluster. The proposed gene functions are listed in Table 1. **b** UPLC analysis of PM100117 (*1*) and PM100118 (*2*) production in *Streptomyces caniferus* GUA-06-05-006A wild type (GUA) and mutant *gonP1* ^−^

PM100117/18 chemical structures have been previously elucidated by nuclear magnetic resonance (NMR) spectroscopy [25]. They contain a 48 carbons aglycone that is likely biosynthesized by the condensation of 21 ketide moieties. The aglycone forms a 36-membered macrolactone ring and is decorated with a side chain consisting of three 2,6-dideoxy sugars and a 1,4-naphtoquinone chromophore (Fig. 1). The first sugar moiety attached to the aglycone is l-axenose, which can be linked either to l-2-deoxy-fucose (PM100117) or l-rhodinose (PM100118) as second sugar. The second sugar unit is linked to the naphtoquinone moiety, which shares a clear structural similarity with menaquinone (MK). An l-rhodinose moiety attached to the naphtoquinone structure represents the third deoxysugar of the PM100117/18 glycosylation pattern. Based on these structural features, glycosyltransferase and sugar biosynthetic genes, as well as a minimum of 21 PKS modules, are presumably involved in the biosynthesis of PM100117/18. Among the seven PKS gene clusters identified by antiSMASH, one of them contained ORFs designated with such putative functions, representing the most suitable candidate cluster to accomplish PM100117/18 biosynthesis. The putative PM100117/18 cluster covers a 171 kb region and contains 54 ORFs (Fig. 2a) coding for proteins with the putative functions listed in Table 1. The involvement of this cluster in PM100117/18 biosynthesis was demonstrated by inactivation of the PKS gene *gonP1*. The analysis of the resulting mutant strain showed that *gonP1* inactivation abolished PM100117/18 biosynthesis, thus confirming the implication of this cluster in the production of PM100117/18 (Fig. 2b).

**Table 1:** Deduced functions of ORFs in PM100117 and PM100118 biosynthetic gene cluster

Gene	Deduced aa. length	Protein homologue (accession no.)	Identity/similarity (%)	Proposed function
*orf1*	264	WP_030324639.1	84/92	Aspartate dehydrogenase
*orf2*	265	WP_030324637.1	77/83	Hydrolase
*orf3*	173	WP_018535062.1	94/98	Cupin domain-containing protein
*orf4*	228	EDY62802.1	81/86	3-Ketoacyl-(acyl-carrier-protein) reductase
*orf5*	260	WP_005320771.1	83/89	Short-chain dehydrogenase
*orf6*	541	WP_033304767.1	82/87	Acetolactate synthase
*orf7*	313	WP_005320767.1	79/85	Ferredoxin
*orf8*	75	WP_031101148.1	70/75	Hypothetical protein
*orf9*	337	WP_033304770.1	88/93	3-Phenylpropionate dioxygenase
*gonMR*	176	WP_030624728.1	92/95	MarR family transcriptional regulator
*gonL1*	959	WP_006607813.1	86/90	LuxR family transcriptional regulator
*gonL2*	241	EJJ02892.1	80/84	LuxR family transcriptional regulator
*gonL3*	939	WP_006607811.1	80/87	LuxR family transcriptional regulator
*gonD1*	469	WP_006607808.1	90/94	NDP-hexose 2,3-dehydratase
*gonP1*	4219	AJE81080.1	60/69	Type I polyketide synthase (loading module and modules 1 and 2)
*gonP2*	4939	AAZ94388.1	57/68	Type I polyketide synthase (modules 3 to 5)
*gonP3*	3959	EJJ02484.1	86/89	Type I polyketide synthase (modules 6 and 7)
*gonP4*	5234	EJJ02491.1	86/90	Type I polyketide synthase (modules 8 to 10)
*gonP5*	8461	EDX25447.1	55/67	Type I polyketide synthase (modules 11 to 15)
*gonP6*	5238	EJJ02498.1	87/90	Type I polyketide synthase (modules 16 to 18)
*gonP7*	3641	EJJ02497.1	87/91	Type I polyketide synthase (modules 19 and 20)
*gonF1*	67	WP_006608219.1	76/80	Ferredoxin
*gonG1*	415	ABO28820.1	66/80	Glycosyltransferase
*gonGS*	358	WP_006608216.1	94/97	Glucose-1-phosphate thymidylyltransferase (glucose synthase)
*gonD2*	333	WP_040902050.1	91/93	dTDP-glucose 4,6-dehydratase
*gonR1*	356	WP_051007140.1	70/79	NDP-4-keto-6-deoxyhexose reductase
*gonG2*	408	WP_006608213.1	74/83	Glycosyltransferase
*gonD3*	433	BAP34744.1	73/84	NDP-hexose-3,4-dehydratase
*gonSL*	1107	WP_006608212.1	68/78	AMP-dependent synthetase and ligase (acyl-CoA ligase, DH, KR, ACP)
*gonS1*	347	WP_040902110	82/91	3-oxoacyl-ACP synthase III
*gonA*	146	WP_040902049	72/84	Cold-Shock DNA-binding domain-containing protein
*gonG3*	402	WP_006608209	55/68	Glycosyltransferase
*gonMT*	345	WP_014208548	49/62	Methylase involved in ubiquinone/menaquinone biosynthesis
*gonS2*	344	WP_040246457	81/90	3-Oxoacyl-ACP synthase III
*gonP8*	2408	WP_006608212	58/59	Type I polyketide synthase (loading module and extension module 1)
*gonG4*	368	WP_042158955	89/93	Glycosyltransferase
*gonT1*	286	WP_040902100.1	83/92	Multidrug ABC transporter permease
*gonT2*	260	WP_006608193.1	89/94	Multidrug ABC transporter permease
*gonR2*	319	AAB63047.1	49/63	dTDP-4-keto-2,3,6-trideoxyhexulose reductase
*gonE*	211	WP_006608191.1	83/89	dTDP-4-deoxyglucose 3,5-epimerase
*gonCM*	410	AKT74284.1	79/87	NDP-hexose-3-C-methyltransferase
*gonH1*	252	WP_006608189.1	79/88	Hypothetical protein
*gonT*	256	WP_006608180.1	90/94	Thioesterase
*gonM1*	301	WP_046708157.1	77/86	Menaquinone biosynthesis protein (chorismate dehydratase)
*gonM2*	407	EJJ02566.1	96/97	Menaquinone biosynthesis protein (dehypoxanthine futalosine cyclase)
*gonF2*	101	WP_006608177.1	94/96	Ferredoxin
*gonM3*	387	WP_006608176.1	98/99	Menaquinone biosynthesis protein (aminofutalosine synthase)
*gonM4*	291	WP_006608174.1	87/91	Menaquinone biosynthesis protein (1,4-dihydroxy-6-naphthoate synthase)
*gonR3*	364	WP_006608173.1	91/94	NDP-hexose 3-ketoreductase
*gonCP*	482	WP_006608172.1	91/95	Cytochrome P450
*orf10*	264	WP_030985702.1	88/92	3-Oxoacyl-ACP reductase
*orf11*	128	WP_053688399.1	86/91	HxlR family transcriptional regulator
*orf12*	174	WP_051733431.1	93/96	Hypothetical membrane protein
*orf13*	355	WP_037651728.1	90/93	NADPH: quinone reductase

### *In silico* analysis of PM100117 and PM100118 gene cluster and proposed biosynthesis pathway

#### Polyketide ring and post-PKS modifications

**Fig. 3:**
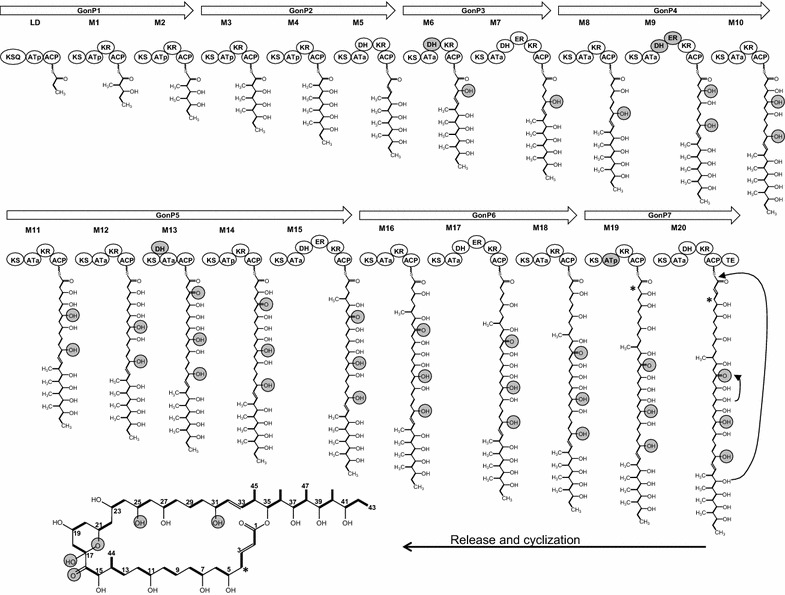
Proposed biosynthesis pathway of PM100117 and PM100118 polyketide skeleton. *LD* loading domain, *M1–M20* extension modules. Polyketide synthase domains are as follows: *KS* ketosynthase, *AT* acyltransferase, *KR* ketoreductase, *DH* dehydratase, *ER* enoylreductase, *ACP* acyl-carrier-protein. *Grey circles* indicate inconsistencies between catalytic domains of the PKS modules and chemical functional groups in the polyketide. *Numbers* indicate aglycone carbon positions and *asterisks* mark the carbon where presence of a methyl group was expected

The PM100117/18 cluster encompasses seven contiguous PKS genes (*gonP1*–*gonP7*) coding for a multimodular PKS that comprises a loading domain (LD) and 20 extension modules (M1–M20), in agreement with the 21 condensation steps required for the biosynthesis of the PM100117/18 macrolide ring. Sequence analysis of GonP1–GonP7 allowed to define ketosynthase (KS or KSQ), acyltranferase (AT), ketoreductase (KR), dehydratase (DH), enoylreductase (ER) and acyl-carrier-protein (ACP) domains. LD contains a ketosynthase (KSQ) domain, in which the essential cysteine of the conserved DTxCSxS sequence at the active site is replaced by glutamine [28]. Sequence alignment of the cluster PKS domains active sites is shown as supplementary data (Additional file 1: Figure S1). AntiSmash analysis also predicted substrate specificity of AT domains within the modular PKS for methylmalonyl-CoA (ATp) in seven modules (LD, M1–M4, M14 and M19) and for malonyl-CoA (ATa) in 14 modules (M5–M13, M15–M18 and M20). The organization of the PKS genes and the proposed biosynthesis pathway of the PM100117/18 macrolide backbone are shown in Fig. 3. Overall, the predicted substrate specificity of AT domains alongside the disposal of the defined KR, DH and ER domains are consistent with the hypothetical elongation through collinear reactions of the polyketide chain. Only a few discrepancies are found between this biosynthesis model and the chemical structure of the polyketide moiety, suggesting the inactivity/inability of certain PM100117/18 PKS domains or the occurrence of post-PKS modifications. The presence of a DH domain in M6 (GonP3), as well as a DH and an ER domain in M9 (GonP4), apparently disturbs the collinear biosynthesis of the polyketide, given the presence of hydroxyl groups at carbon (C) 31 and C25 of the polyketide skeleton (Fig. 3). Nonetheless, a detailed sequence analysis of the M6 DH domain revealed an arginine in place of an otherwise conserved histidine in the NADPH motif LxxHxxxGxxxxP [29] at the active site (Aditional file 1: Figure S1). This, together with a shorter C-terminal sequence in comparison with other DH domains within the cluster PKSs, suggests that M6 DH is most possibly inactive. By contrast, sequence analysis did not show any alteration of the M9 DH and ER domains, which are probably functional. Thus, the unexpected presence of these domains could be explained by a “domain skipping” mechanism preventing dehydrogenation of C25 during polyketide elongation [30]. Alternatively, the presence of a hydroxyl group at C25 can be the result of a post-PKS modification following polyketide biosynthesis, catalyzed by any of the two putative oxygenases coded by *gonCP* and *orf9*. Moreover, the involvement of the M13 DH domain (GonP5) in the polyketide biosynthesis is unlikely due to the lack of a KR domain in this module catalyzing a previous ketoreduction reaction. One last inconsistency is found between the substrate specificity of the AT domain of M19 (GonP7), which is predicted to utilize methylmalonyl-CoA as extension unit, and the absence of a methyl group at C4 (Fig. 3). However, the presence in this domain of a YASH motif, which specifies utilization of methylmalonyl-CoA [31], can be confirmed as shown in the alignment of cluster AT active sites (Additional file 1: Figure S1). Despite this, no PM100117/18 analogues containing an additional methyl group at C4 have been detected in *S. caniferus* GUA-06-05-006A cultures.

#### Sugars biosynthesis

**Fig. 4:**
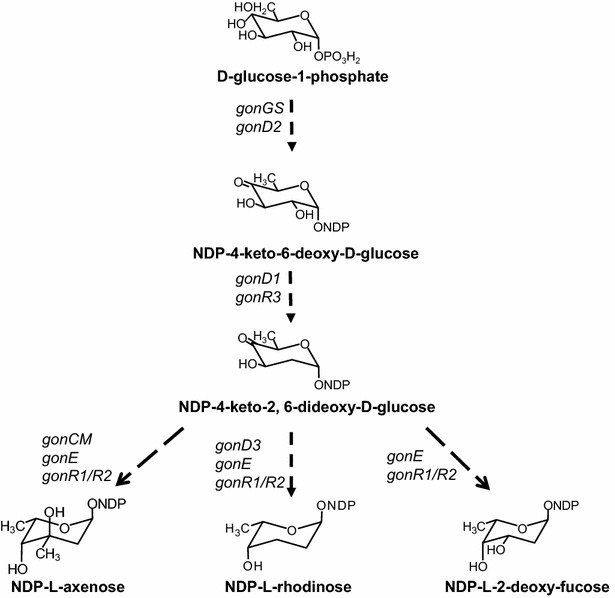
Schematic representation of the proposed biosynthesis pathway of PM100117 and PM100118 deoxysugar moieties

A distinctive feature of the PM100117/18 biosynthesis gene cluster, among the 32 clusters for secondary metabolites hosted by *S. caniferus* GUA-06-05-006A, is the presence of ORFs with assigned putative functions implicated in biosynthesis or transfer of deoxysugars. Based on these putative functions the biosynthesis pathway of l-axenose, l-2-deoxi-fucose and l-rhodinose can be predicted (Fig. 4). Proteins GonGS and GonD2, putative NDP-glucose synthase and NDP-glucose 4,6-dehydratase, respectively, might catalyze the biosynthesis of the key intermediate NDP-4-keto-6-deoxy-d-glucose [32], which should be then transformed into NDP-4-keto-2,6-dideoxy-d-glucose (2,6-DG) by the activity of the putative NDP-hexose 2,3-dehydratase GonD1 and the putative NDP-hexose 3-ketoreductase GonR3. Biosynthesis of l-2-deoxi-fucose from 2,6-DG requires 3,5-epimerization (3,5-EPI) and C4-ketoreduction (C4-KR) reaction steps, which are possibly catalyzed by the putative dTDP-deoxyglucose 3,5-epimerase GonE and one of the putative NDP-4-keto-6-deoxyhexose reductases GonR1 or GonR2, respectively. In addition to 3,5-EPI and C4-KR reactions, biosynthesis of l-axenose from 2,6-DG involves a C3-metylation step, presumably catalyzed by the putative NDP-hexose-3-C-methyltransferase GonCM. Moreover, biosynthesis of l-rhodinose requires the C3-dehydration reaction of 2,6-DG, possibly catalyzed by the putative NDP-hexose-3,4-dehydratase enzyme GonD3, followed by 3,5-EPI and C4-KR. After macrolactone formation, four putative glycosyltransferase-coding genes (*gonG1*, *gonG2*, *gonG3* and *gonG4*) could be involved in the transfer of the three deoxysugar moieties to generate PM100117/18.

#### Naphthoquinone (NQ) biosynthesis

A further peculiarity of the PM100117/18 biosynthesis cluster is the presence of four ORFs (*gonM1*, *gonM2*, *gonM3* and *gonM4*) with high identity to genes previously reported as involved in menaquinone biosynthesis via futalosine. This newly discovered MK pathway was first described in *S. coelicolorA3*(*2*) [33–35] but bioinformatics analyses suggest its presence also in other bacteria lacking the classic MK biosynthetic pathway via isochorismate [33, 36, 37]. Sequence analysis reveals a high degree of identity (I) and similarity (S) of GonM1, GonM2 and GonM4 to the *S. coelicolor* A3(2) proteins SCO4506/MqnA (I: 60.1 %, S: 74.4 %), SCO4550/MqnC (I: 85 %, S: 91.6 %) and SCO4326/MqnD (I: 69.5 %, S: 77.1 %), respectively. These *S. coelicolor* A3(2) proteins, together with SCO4327/MqnB, have been previously shown to be involved in the biosynthesis of the futalosine MK pathway intermediate 1,4-dihydroxy 6-napthoic acid (DH6N) [33–35]. Furthermore, GonM3 shares sequence resemblance with SCO4494/MqnE (I: 93 %, S: 96.4), a protein proposed to catalyze the biosynthesis of aminodeoxyfutalosine [38], an alternative substrate for MK biosynthesis. An additional copy of genes coding for proteins with high identity and similarity to *S. coelicolor* A3(2) MqnA (I: 56.6 %, S: 62.2 %), MqnB (I: 68.1 %, S: 72.9 %), MqnC (I: 92.7 %, S: 97.0 %) and MqnD (I: 72.2 %, S: 75.7 %) has been detected in the *S. caniferus* GUA-06-05-006A genome, outside the PM100117/18 gene cluster. The structural similarity of DH6N with the NQ unit led us to suspect a role of genes *gonM1*–*gonM4* on the biosynthesis of this compound. Other ORFs potentially involved in the biosynthesis of NQ code for AMP-dependent synthetase and ligase GonSL, 3-oxoacyl-ACP synthase III GonS1 and GonS2, type I PKS GonP8 and methyltransferase GonMT. GonSL contains an apparently active CoA ligase (CAL) and ACP domain, and a KR and DH domain with important amino acid substitutions (Additional file 1: Figure S1). The putative PKS gene *gonP8* consists of a loading module containing a CAL and an ACP domain, and an extension module with a KS, ATp, KR, DH and ACP domain, all of them with no significant active site amino acid replacements (Additional file 1: Figure S1).

**Fig. 5:**
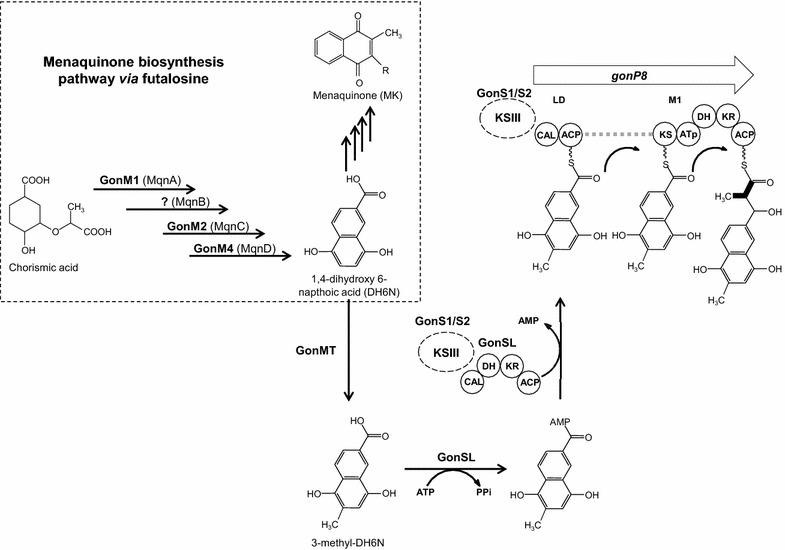
Proposed biosynthesis pathway of PM100117 and PM100118 naphthoquinone moiety. *LD* loading domain, *M1* extension module. Polyketide synthase domains are as follows *KS* ketosynthase, *AT* acyltransferase, *KR* ketoreductase, *DH* dehydratase, *ACP* acyl-carrier-protein, *CAL* CoA ligase, *KSIII* 3-oxoacyl-ACP synthase III

The proposed biosynthesis pathway of the PM100117/18 NQ moiety is depicted in Fig. 5. GonM1 (MqnA), GonM2 (MqnC), GonM4 (MqnD) and a futalosine hydrolase (MqnB) enzyme coded by a gene external to the PM100117/18 cluster could catalyze the biosynthesis of DH6N, which would be then methylated by the putative methyltransferase GonMT to form 3-methyl-DH6N (Fig. 5). Interestingly, GonMT is only 11 % identical to SCO4556, which is the enzyme proposed to catalyze the quinone-ring C2-methylation in the last step of MK biosynthesis [33–35]. There is no available information to deduce the possible mechanism by which 3-methyl-DH6N is elongated with a propionate unit to form the NQ moiety. However, based on the fatty acid activation process catalyzed by fatty acyl-AMP ligases [39], we could speculate that the putative synthetase-ligase GonSL could catalyze the synthesis of a 3-methyl-DH6N-AMP adduct, and the subsequent transfer of 3-methyl-DH6N to the pantetheine group of its own ACP domain. In a next step, 3-methyl-DH6N might be transferred to the LD of PKS GonP8, and then elongated by the GonP8 extension module. Binding of 3-methyl-DH6N-AMP to the GonSL ACP domain, and its following transfer to the LD of PKS GonP8, may require the participation of any of the putative 3-oxoacyl-ACP synthase III (KSIII) enzymes coded by genes *gonS1* and *gonS2*. The involvement of KSIII proteins in the priming of starter units alternative to malonyl-CoA and methylmalonyl-CoA has been previously reported in the biosynthesis of a number of compounds [40–42]. Likewise, the mechanism by which NQ is eventually transferred to PM100117/18 may also require intervention of KSIII, GonP8 or both.

#### Pathway regulation

Four ORFs (*gonMR*, *gonL1*, *gonL2 and gonL3*) could be responsible for PM100117/18 pathway regulation as they code for proteins with high sequence resemblance to transcription regulatory proteins. GonMR contains a helix-turn-helix (HTH) motif (smart00347) highly conserved among members of the MarR family of protein regulators. GonL1 and GonL3 contain a N-terminal nucleoside triphosphate binding motif (pfam13191) and a HTH domain of the LuxR-type at de C terminus (smart00421), which are distinctive functional features of the LAL family of transcription factors [43, 44]. In addition to a LuxR-like C-terminal HTH motif, GonL2 contains a PAS-sensor binding fold at the N terminus (cd00130). This domain is designated PAS because of its homology to the *Drosophila* period protein (Per), the aryl hydrocarbon receptor nuclear translocator protein (ARNT) and the *Drosophila* single-minded protein (Sim) [45].

#### PM100117 and PM100118 transport

ORFs *gonT1* and *gonT2* code for a putative ATP-binding cassette (ABC) transport system. ABC transport complexes are comprised of a hydrophilic protein containing an ATP-binding domain and a hydrophobic protein with six membrane-spanning domains [46]. GonT1 shares high sequence identity and similarity with ABC-type membrane permeases and its analysis with the TMHMM server predicts the formation in this protein of six hydrophobic transmembrane domains. Conversely, GonT2 displays an ABC-like nucleoside triphosphate hydrolase domain (cl21455), which in combination with the putative permease GonT1 could produce a complex to facilitate PM100117/18 transport across the membrane.

### Cluster boundaries analysis

**Fig. 6:**
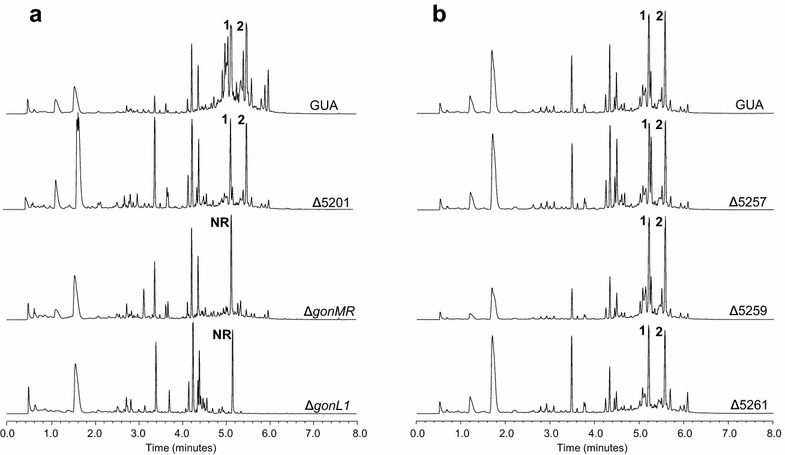
Cluster boundaries delimitation. UPLC analysis of PM100117 (*1*) and PM100118 (*2*) production in *Streptomyces caniferus* GUA-06-05-006A wild-type (GUA) and mutant strains **a** Δ5201, Δ*gonMR* and Δ*gonL1* (*left border*) and **b** Δ5257, Δ5259 and Δ5261 (*right border*). *NR* compound not related to PM100117/18

On the cluster left side, genes *gonMR, gonL1, gonL2 and gonL3* were presumed to code for pathway-specific regulators for PM100117/18 biosynthesis, and *orf9*, which codes for a putative dioxygenase, was a candidate gene to accomplish polyketide post-PKS oxygenations. Thus, to verify this boundary, we performed ultra-performance liquid chromatography (UPLC) analysis of PM100117/18 production in mutant strains Δ*gonL1*, Δ*gonMR* and Δ5201 in which *gonL1*, *gonMR* and *orf9*, respectively, have been deleted. The result of this analysis showed that in Δ5201 PM100117/18 production was not altered with respect to *S. caniferus* GUA-06-05-006A wild type. By contrast, in Δ*gonL1* and Δ*gonMR*, PM100117/18 biosynthesis was absent or severely decreased (Fig. 6a). Curiously, deletion of these genes induced the production of a new compound (NR), with a maximum absorption wavelength at 260 nm, apparently not related to PM100117/18 biosynthesis. Since PM100117/18 derivatives lacking small functional groups could possess retention times close to those of the parental products, Δ5201 extracts were also analyzed by LC-MS, confirming that peaks labeled as **1** and **2** indeed correspond to PM100117 and PM100118, respectively. These results verified the involvement of genes *gonL1* and *gonMR* in PM100117/18 biosynthesis, presumably coding for positive pathway-specific transcriptional regulators. PM100117/18 production was partially recovered in mutant strains Δ*gonL1* and Δ*gonMR* when a copy of *gonL1* and *gonMR*, respectively, was re-introduced (Additional file 1: Figure S2). The cluster left border is thus apparently defined by *gonMR*. Upstream to this gene, antiSMASH detected nine ORFs coding for proteins with putative functions (Table 1) likely not pertaining to PM100117/18 biosynthesis. Most of these putative activities are related to oxidation-reduction reactions, such as ferredoxin (*orf7*), short-chain dehydrogenase (*orf5*), 3-ketoacyl-ACP reductase (*orf4*) and aspartate dehydrogenase (*orf1*), as well as a cupin domain-containing protein (*orf3*).

On the cluster right side, gene *gonCP* has been demonstrated by genetic engineering to be involved in the polyketide post-PKS oxygenation. Detailed data on this finding are described in section below. Adjacent to *gonCP*, antiSMASH detected ORFs coding for proteins with putative activities 3-oxoacyl-ACP reductase (*orf10*), HxlR family transcriptional regulator (*orf11*), hypothetical membrane protein (*orf12*) and NADPH-dependent quinone reductase (*orf13*). Hence, in order to verify the cluster right boundary, strains Δ5257, Δ5259 and Δ5261, which lack *orf10*, *orf11* and *orf13*, respectively, were assessed for PM100117/18 production by UPLC. As shown in Fig. 6b, deletion of these genes does not have any effect on PM100117/18 biosynthesis, confirming that they do not belong to the cluster. Therefore, the right cluster boundary seems to be defined by gene *gonCP*.

### Generation and characterization of novel PM100117/18 derivatives

**Fig. 7:**
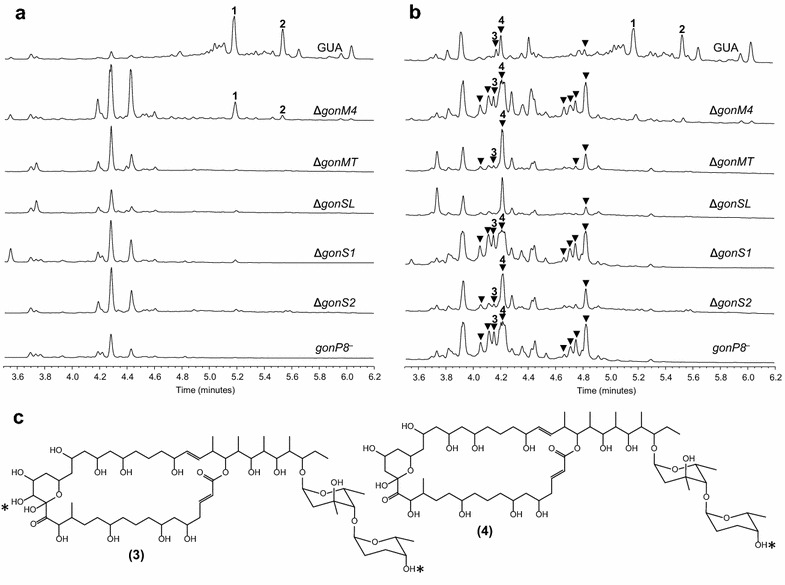
Characterization of genes involved in the biosynthesis of the PM100117 and PM100118 naphthoquinone unit. Analysis of PM100117 (*1*) and PM100118 (*2*) production by UPLC at 254 nm (**a**) and 216 nm (**b**) in *Streptomyces caniferus* GUA-06-05-006A wild type (GUA) and Δ*gonM4*, Δ*gonMT*, Δ*gonSL*, Δ*gonS1*, Δ*gonS2*, and *gonP8* ^−^ mutant strains. Peaks with an absorption spectrum compatible with PM100117 and PM100118 derivatives lacking the NQ moiety are tagged with *triangles*. **c** Chemical structures of PM100118 derivatives lacking the naphthoquinone moiety. *Asterisks* indicate the point where the PM100118 chemical structures have been modified

The main goal of this work was engineering novel PM100117/18 analogues with improved antitumor properties. We first sought to obtain structural analogues lacking the NQ moiety. For that purpose, a series of mutant strains affected in NQ biosynthesis putative genes (Fig. 5) was generated by disruption of *gonP8*, or individual deletion of *gonM4*, *gonMT*, *gonSL*, *gonS1* or *gonS2*. The resulting strains, *gonP8*^−^, Δ*gonM4*, Δ*gonMT*, Δ*gonSL*, Δ*gonS1* and Δ*gonS2*, were examined for PM100117/18 production by UPLC at 254 nm (Fig. 7a). These analyses did not detect PM100117/18 biosynthesis in *gonP8*^−^, Δ*gonMT*, Δ*gonSL*, Δ*gonS1* and Δ*gonS2.* Only in Δ*gonM4* some traces of PM100117/18 were detected (Fig. 7a). To assess whether the reason of these changes in the PM100117/18 production level was the absence or decrease of NQ biosynthesis, we examined the accumulation of PM100117/18 intermediates lacking the NQ moiety in the mutant strains. It is important to note that loss of the NQ unit causes a change in the maximum absorption wavelength with respect to the parental compounds, shifting from 254 to 216 nm. UPLC and LC-MS analysis at 216 nm detected in the six mutant strains several compounds (Fig. 7b, triangles) with the expected absorption spectra. In addition, two of these products (**3** and **4**) possessed molecular weights compatible with PM100117 [**1**, UPLC R_t_ = 5.182 min, m/z 1601.9 (*M* + Na)^+^] and PM1001118 [**2**, UPLC R_t_ = 5.536 min, m/z 1585.9 (*M* + Na)^+^] biosynthetic intermediates lacking the NQ moiety. The chemical structures of compounds **3** [UPLC R_t_ = 4.14 min, m/z 1231.7 (*M* + Na)^+^] and **4** [UPLC R_t_ = 4.21 min, m/z 1215.7 (*M* + Na)^+^] were determined by NMR (Additional file 2: Figure S5), confirming that both products correspond to PM100118 analogues lacking the NQ moiety (Fig. 7c). Interestingly, compound **3** carries an additional hydroxyl group at the aglycone C18, which is not present in any of the parental products, suggesting that this derivative could belong to a PM100118 shunt biosynthetic pathway. Mutant complementation with the corresponding genes partially restored PM100117/18 production (Additional file 1: Figure S2). These results confirm the involvement of genes *gonM4*, *gonMT*, *gonSL*, *gonS1*, *gonS2* and *gonP8* in the biosynthesis and/or transfer of the NQ unit to PM100117/18.

The in vitro antitumor activity of **3** and **4** was determined by measuring their GI_50_ (50 % inhibition on cell growth), TGI (total growth inhibition) and LC_50_ (50 % cell death) concentrations [47], against cancer cell lines A549 (human lung carcinoma cells), PSN1 (pancreas carcinoma), MDA-MB-231 (human breast adenocarcinoma) and HT29 (human colorectal carcinoma). The values of these three antitumor indicators for compounds **3** and **4** were remarkably higher than those of PM100117/18, indicating a weaker cytotoxic activity of the derivative compounds in comparison with the natural drugs (Table 2).

**Table 2:** In vitro antitumor activity of compounds **1**–**6**

Compounds	A549 (µM)	HT29 (µM)	MDA-MB-231 (µM)	PSN1 (µM)
**1** PM100117				
GI_50_	1.52	3.04	2.66	nd
TGI	1.84	3.23	2.79	nd
LC_50_	2.22	3.61	2.97	nd
**2** PM100118				
GI50	2.24	1.92	1.73	nd
TGI	3.13	2.81	2.75	nd
LC_50_	4.28	4.09	4.16	nd
**3** from mutant *gonP8* ^−^				
GI_50_	>8.40	>8.40	>8.40	>8.40
TGI	>8.40	>8.40	>8.40	>8.40
LC_50_	>8.40	>8.40	>8.40	>8.40
**4** from mutant *gonP8* ^−^				
GI_50_	>8.40	>8.40	>8.40	>8.40
TGI	>8.40	>8.40	>8.40	>8.40
LC_50_	>8.40	>8.40	>8.40	>8.40
**5** from mutant ∆*gonCP*				
GI_50_	0.38	0.13	0.71	0.90
TGI	0.54	1.80	0.83	0.96
LC_50_	0.77	2.50	1.03	1.03
**6** from mutant ∆*gonCP*				
GI_50_	1.11	3.39	2.60	2.80
TGI	1.17	4.30	2.80	3.00
LC_50_	1.24	5.48	3.00	3.19

*GI*_*50*_ compound concentration that produces 50 % inhibition on cell growth as compared to control cells, *TGI* compound concentration that produces total growth inhibition as compared to control cells, *LC*_*50*_ compound concentration that produces 50 % cell death as compared to control cells, *Nd* values not determined

**Fig. 8:**
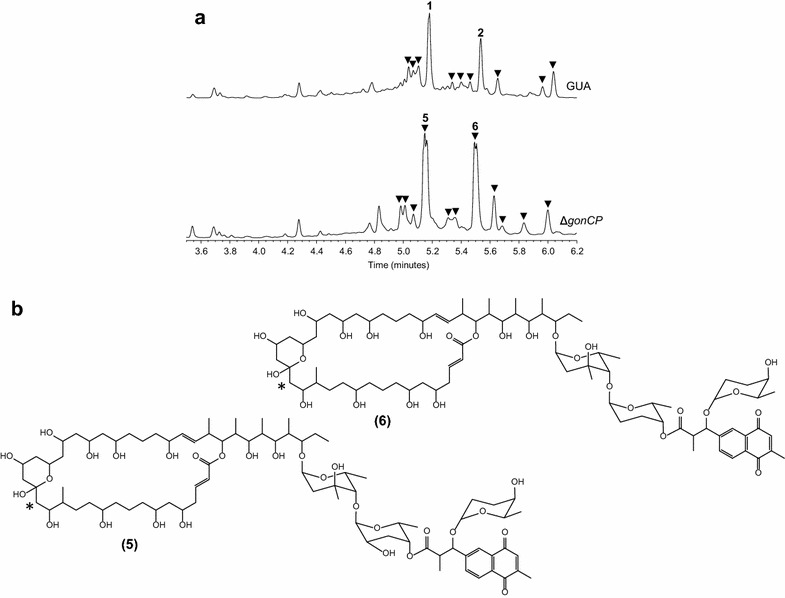
Characterization of cytochrome P450 monooxygenase gene *gonCP*. **a** UPLC analysis of PM100117 (*1*) and PM100118 (*2*) production in *Streptomyces caniferus* GUA-06-05-006A wild type (GUA) and Δ*gonCP* mutant. Peaks with an absorption spectrum related to PM100117 and PM100118 derivatives are tagged with* triangles*. **b** Chemical structures of PM100117 and PM100118 derivatives lacking a keto functional group of the macrolactone moiety. *Asterisks* indicate the point where the PM100117 and PM100118 chemical structures have been modified

Given the cytotoxicity decay caused by the loss of the NQ moiety, we sought to perform a genetic manipulation leading to minor modifications of the PM100117/18 structure while preserving the NQ unit. With this aim, we deleted *gonCP*, which codes for a putative cytochrome P450 monooxygenase potentially involved in the oxygenation of the aglycone C16 and C17 (Fig. 3). UPLC analysis revealed the ability of the resulting mutant strain, Δ*gonCP*, to produce several compounds (triangles, Fig. 8a) with absorption spectra related to PM100117/18. Furthermore, analysis of fermentation extracts from Δ*gonCP* by LC-MS showed that two of these compounds, **5** [UPLC R_t_ = 5.14 min, m/z 1587.9 (*M* + Na)^+^] and **6** [UPLC R_t_ = 5.50 min, m/z 1571.9 (*M* + Na)^+^], possess molecular weights corresponding to PM100117 (**1**) and PM100118 (**2**) analogues, respectively, lacking a keto group. Compounds **5** and **6** were then purified and analyzed by NMR to determine their chemical structures (Additional file 2: Figure S5), confirming that they derive from PM100117 and PM100118, respectively, by loss of the C16 aglycone keto functional group (Fig. 8b). This confirms the implication of gene *gonCP* in C16 oxygenation, but the question on the enzyme that catalyzes C17 hydroxylation still remains. The production of the natural compounds was restored when *gonCP* was re-introduced in the Δ*gonCP* mutant (Additional file 1: Figure S2).

Likewise compounds **3** and **4**, the antitumor activity of derivatives **5** and **6** was examined against various cancer cell lines. Interestingly, compound **5** possesses an in vitro cytotoxicity threefold to fourfold higher than its corresponding parental product (**1**, PM100117) against A549 and MDA-MB-231 cell lines, as indicated by the lower GI_50_, TGI and LC_50_ concentration values (Table 2). The GI_50_ concentration of this compound also showed an outstanding 23-fold increase of antitumor activity, relative to PM100117, against HT29 tumor cells. However, this result is not concomitant with a similar decrease of the TGI and LC_50_ concentrations, which keep values close to that of compound PM100117. Additional analysis of **5** by antibiotic activity assays against *Micrococcus luteus* and *Saccharomyces cerevisiae* further confirms an enhanced bioactivity of this derivative with respect to the parental products (Additional file 1: Figure S3). PM100118 analogue **6** showed a twofold to threefold improvement in the in vitro cytotoxic activity against the A549 cell line, as revealed by its lower GI_50_, TGI and LC_50_ values relative to the natural compound (**2**, PM100118).

## Discussion

In this work, we have identified the PM100117 and PM100118 biosynthetic gene cluster, which has been characterized on the basis of bioinformatics analysis and genetic engineering data. The cluster spans a region of 169 kb and contains 41 genes coding for all the putative functions presumably required for PM100117/18 biosynthesis (Fig. 2; Table 1). From these activities, biosynthesis of PM100117/18 can be predicted as follows. Firstly, a type I multimodular PKS containing a loading module and 20 extension modules, catalyzes the 21 condensation reactions required for the biosynthesis of the aglycone polyketide chain (Fig. 3). The macrolatone moiety is then decorated with two 2,6-dideoxysugar moieties. Next, the NQ unit is transferred to the second deoxysugar and then glycosylated with the third 2,6-dideoxysugar. The presence in the cluster of four putative glycosyltransferase genes is consistent with the PM100117/18 glycosylation pattern. One of the four glycosyltransferases might transfer l-axenose to the first position of the glycosylation profile. Two glycosyltransferases might be responsible for the transfer of l-2-deoxy-fucose (PM100117) or l-rhodinose (PM100118) to the second glycosylation position. Finally, the last glycosyltransferase would transfer l-rhodinose to the NQ moiety. Carbons 16 and 17 of the aglycone contain a keto and a hydroxyl group, respectively. These oxygenations could be introduced at any step of PM100117/18 biosynthesis, after completion of the polyketide biosynthesis, as a tailoring modification. We have shown that the putative cytochrome P450 monooxygenase GonCP is responsible, at least, for C16 oxygenation (Fig. 8). There is not a clear candidate for C17 oxygenation, being the closest *orf9*. Deletion of *orf9*, which codes for a putative dioxygenase and lies outside the PM100117/100118 cluster boundaries, does not show any effect on C17 hydroxylation (Fig. 6). However, the involvement of *gonCP* acting as a multifunctional oxygenase in C17 modification cannot be excluded. The oxygenation of consecutive carbons at polyketide aglyca by multifunctional cytochrome P450 monooxygenases has been previously reported [48]. Taking this into consideration we cannot discard the possibility of *orf9* product, or any other oxygenase coding gene at *S. caniferus* GUA-06-05-006A chromosome, complementing the putative GonCP C17 oxygenation in Δ*gonCP* mutant strain. Four genes code for putative transcriptional regulators belonging to the MarR (GonMR) and LuxR (GonL1, GonL2 and GonL3) family, indicating that PM100117/18 biosynthesis might be subjected to a tight transcriptional regulation. Genetic engineering data suggest that proteins GonMR and GonL1 act as putative transcriptional activators. This is an interesting finding because MarR transcriptional activators have been rarely described in the literature [49]. However, other mechanisms can also be considered regarding the mode of action of GonMR, such as a dual role as activator-repressor [50] or co-activator.

The noticeable structural similarity of the NQ unit to MK induced us to envisage a connection between the biosynthesis of both compounds (Fig. 5). This hypothesis is further supported by the presence in the cluster of four genes (*gonM1*, *gonM2*, *gonM3* and *gonM4*), coding for proteins highly identical to those involved in the early steps of MK biosynthesis via futalosine [33–35]. Based on their assigned putative functions, GonM1 (MqnA), GonM2 (MqnC) and GonM4 (MqnD) could catalyze three of the four reaction steps leading to the biosynthesis of DH6N, which, according to our model, defines the branching point towards NQ and MK biosynthesis (Fig. 5). Even though C2-methylation of the NQ quinone ring could occurs at any step of its biosynthesis, it is very plausible that this reaction delivers DH6N to the NQ branch. Deletion of *gonM4* diminishes PM100117/18 production, and leads to the accumulation of biosynthetic intermediates lacking the NQ unit (Fig. 7c). This result confirms the involvement of *gonM4* in NQ biosynthesis. However, in contrast to *gonP8*^−^, Δ*gonMT*, Δ*gonSL*, Δ*gonS1* and Δ*gonS2* mutant strains, in which PM100117/18 biosynthesis is completely abolished, in Δ*gonM4* a low PM100117/18 production level can be detected (Fig. 7a). This indicates that the additional *mqnD* homologue, present outside the PM100117/18 gene cluster, is functional and able to partially complement the loss of *gonM4*. We do not fully understand the role of duplicated MqnA, MqnC and MqnD gene functions in the genome. However, we can speculate that the presence in the cluster of dedicated *mqnA*, *mqnC* and *mqnD* genes leading to DH6N biosynthesis could offers two main advantages. One is to provide sufficient supply of DH6N, which is probably a limiting intermediate as it is common to MK and NQ biosynthesis. The second advantage could be to facilitate a coordinated regulation with other genes involved in PM100117/18 biosynthesis, thus ensuring an optimal PM100117/18 production when these are required. To date, this is the first time that MK biosynthesis is described as a nexus between secondary and primary metabolism. Nonetheless, the existence of duplicated gene homologues in a single genome, one being part of the primary metabolism and the other present in a secondary metabolites gene cluster, has been frequently described for genes (*ccr*) coding for crotonyl-coA carboxylase/reductase. Multiple *ccr* homologous have been identified in gene clusters for the biosynthesis of various polyketide natural products [51–54]. As we have speculated for the presence of MK biosynthesis genes in the PM100117/18 gene cluster, the role of *ccr* duplications has also been postulated to be the supply of sufficient precursor building blocks for polyketide biosynthesis [51].

The generation of PM100117/18 structural analogues with improved antitumor activity relative to their natural products, represents a substantial achievement of this work. These derivatives were accomplished by a genetic manipulation, *gonCP* deletion, leading to a minor modification of PM100117/18 structure, the loss of a keto functional group. The twofold to fourfold improvement of antitumor activity showed by these derivatives is an interesting finding because the generation of truncated products, or even the removal of minor structural elements of a natural compound, often affects bioactivity negatively [55]. For instance, deletion of a gene coding for a cytochrome P450 monooxygenase of the pimaricin gene cluster in *S. natalensis* conduces to the production of analogue 4,5-deepoxypimaricin, which differs from the natural compound in a single oxygenation and shows a diminished antibacterial activity relative to the parental product [56]. Similar results have been reported on the pikromycin analogues neopikromycin and narbomycin produced by *S. venezuelae*, which lack a single hydroxyl group at different positions of the polyene ring and possess a noticeably reduced antibacterial activity [57]. Instead, most of the reported genetic engineering approaches, leading to the generation of improved bioactive analogues, consist of the addition or replacement of structural components in the parental product [17, 58]. In this regard, cytochrome P450 monooxygenases have been frequently contemplated as promising targets for engineering the biosynthesis of novel therapeutic natural compounds. As an example, replacement of the C16 carboxyl on the nystatin analogue S44HP with a methyl group by mutation of a P450 monooxygenase gene, yielded a twofold more active antifungal analogue [59]. Herein, compounds **5** and **6** has been only assessed for in vitro cytotoxicity against different tumor cell lines. However, aside from an enhanced bioactivity, structural modifications frequently yield derivatives with other additional desirable pharmacological properties, such as lower toxicity or improved solubility [59–61]. In future works some of these properties of compounds **5** and **6** might also be addressed.

On the other hand, the loss of the NQ unit caused in vitro cytotoxicity decay, indicating that this moiety is central to PM100117/18 antitumor activity. Curiously, two compounds structurally related to PM100117/18, langkolide [26] and GT35 [27], which harbor a similar napthtoquinone moiety, also possess cytotoxic activity. Furthermore, the anti-proliferative effect of MKs on tumor cells both in vitro and in vivo has been repeatedly reported [62, 63]. Conversely, other macrolides structurally resembling PM100117/18 but lacking a napthtoquinone unit, such as liposidolide A [64] and polaramycin [65], exhibit antifungal and antibacterial but not antitumor activity. All together, these observations suggest the idea that PM100117/18 cytotoxic activity could, to large extent, stems from the NQ moiety.

The availability of the PM100117 and PM100118 gene cluster, and the genetic insights into their biosynthesis, will help to understand how similar natural compounds are produced. This information enables the engineering of more derivatives with improved pharmacological properties such as an enhanced biological activity.

## Conclusions

PM100117 and PM100118 are members of a group of glycosylated compounds hallmarked by the presence in their structures of a NQ chromophore structurally resembling MK. Our results show that biosynthesis of the NQ chromophore is a complex process that involves diverse enzymes and which is connected to primary metabolism. The presence in secondary metabolite gene clusters of certain primary metabolism genes may be explained in terms of a sufficient supply of limiting intermediates. This connection of secondary metabolism with MK biosynthesis has never been reported before. A similar situation might be encountered in the future when other gene clusters for natural products structurally related to PM100117/18 will be characterized. On the other hand, the analysis of PM100117/18 analogues has shown interesting insights into the structure-bioactivity relationship of these family of natural products. Removal of the C16 keto group leads to an increased antitumor activity of both the PM100117 and PM100118 derivative. However, the overall cytotoxicity level showed by compound **5** is higher than the observed in compound **6**. This suggests that PM100117 could be a more promising target to undertake other structural modifications. In addition, based on the results presented in this work and previously described data on similar compounds, the presence of NQ moieties might be a predictive structural feature of cytotoxic activity. This issue could be taken into account at future screening for novel antitumor natural products.

## Methods

### Bacterial strains, tumor cell lines, media and cultivation conditions

Unless otherwise indicated, media used in this work have been described in Kieser et al. [66]. The PM100117/18 producer strain *S. caniferus* GUA-06-05-006A [25] was routinely grown in MA medium (2.1 % morpholinepropanesulfonic acid, 0.5 % glucose, 0.05 % yeast extract, 0.05 % meat extract, 0.1 % casaminoacids, pH 7.0 adjusted with KOH). The *Escherichia coli* strains used as host for cloning (DH10B) [67] and for intergeneric conjugation (ET12567/pUB307) [66] were grown in 2× TY medium supplemented, when required, with the appropriate antibiotic for plasmid selection. For metabolite production, a seed culture was grown in 50-ml falcon tubes containing 5 ml TSB medium on a rotary shaker at 250 rpm and 30 °C for 24 h. Then, 1.5 ml of the seed culture was used to inoculate 25 ml of SM medium (1 % glucose, 0.4 % yeast extract, 0.4 % peptone, 0.4 % K_2_HPO_4_, 0.2 % KH_2_PO_4_, 0.05 % MgSO_4_, pH 7.0 adjusted with KOH) supplemented with 10.3 % sucrose in a 250-ml shake flask. The culture was continued at 30 °C for 7 days with constant shaking at 200 rpm. Large-scale fermentations for compound purification were carried out for 9 days in a 2.5 L culture final volume inoculated (5 % v/v) with a seed culture grown for 72 h.

A-549 (ATCC CCL-185), lung carcinoma; HT-29 (ATCC HTB-38), colorectal carcinoma and MDA-MB-231 (ATCC HTB-26), breast adenocarcinoma cell lines were obtained from ATCC (http://www.lgcstandards-atcc.org/). Cell lines were maintained in RPMI medium 1640 (Gibco-RBL) supplemented with 10 % fetal calf serum (FCS), 2 mM l-glutamine and 100 U/mL penicillin and streptomycin, at 37  °C and 5 % CO_2_.

### Analysis of metabolite production and compound purification

Samples (3 ml) from *S. caniferus* GUA-06-05-006A whole-cultures (see above) were mixed with an equal volume of ethyl acetate and incubated at room temperature for 2 h. The organic phase was then recovered by centrifugation (3000×*g*, 10 min) and evaporated *in vacuo*. The residue was dissolved in methanol:DMSO (1:1) to perform UPLC and LC-MS analyses as described elsewhere [68].

For the purification of compounds **3**, **4**, **5** and **6**, mycelia of the corresponding producing strains were separated from the culture by centrifugation and extracted twice with ethyl acetate. The supernatants were filtered and applied to a solid-phase extraction cartridge (Sep-Pak Vac C18, 10 g, Waters) that had been fitted with a perforated stopper pierced by a stainless steel HPLC tubing. The culture broth was applied by means of a peristaltic pump and subsequently the cartridge was connected to a HPLC quaternary pump (model 600E, Waters). The retained material was eluted with a mixture of methanol and 0.05 % trifluoroacetic acid (TFA) in water. A linear gradient from 0 to 100 % methanol in 60 min, at 10 ml/min, was used. Fractions were taken every 5 min, collected on 5 ml of 0.1 M phosphate buffer, pH 7.0 and analyzed by UPLC. Those fractions containing the desired compounds were evaporated *in vacuo* and subsequently re-dissolved in a small volume of a mixture of DMSO and methanol (50:50). The organic extract of the culture pellets was similarly evaporated and re-dissolved. The compounds of interest were purified by preparative HPLC using a SunFire C18 column (10 µm, 10 × 250 mm, Waters). Compounds were chromatographed with mixtures of acetonitrile or methanol and 0.05 % TFA in water in isocratic conditions optimized for each peak, at 7 ml/min, and were always collected on 0.1 M phosphate buffer, pH 7.0. Compound **5** was purified with 55 % acetonitrile in a first step and with 82 % methanol in a second step. Compound **6** was purified with 55 % acetonitrile in a first step and with 85 % methanol in a second step. Compounds **3** and **4** were purified with 32 % acetonitrile in a first step and with 37 % acetonitrile in a second step. After every purification step, the collected compounds were diluted fourfold with water and then applied to a solid-phase extraction cartridge (Sep-Pak C18, Waters). The cartridge was washed with water, the retained compound was eluted with methanol and dried *in vacuo*. Once the purification was finished, the compounds were dissolved in a mixture of tert-butanol and water (1:1) and lyophilized.

### In vitro cytotoxicity assay

Triplicate cultures were incubated for 72 h in the presence or absence of test compounds (at ten concentrations ranging from 10 to 0.0026 mg/mL). For quantitative estimation of cytotoxicity, the colorimetric sulforhodamine B (SRB) method was used [69]. Briefly, cells were washed twice with PBS, fixed for 15 min in 1 % glutaraldehyde solution, rinsed twice in PBS, and stained in 0.4 % SRB solution for 30 min at room temperature. Cells were then rinsed several times with 1 % acetic acid solution and air-dried. Sulforhodamine B was then extracted in 10 mM trizma base solution and the absorbance measured at 490 nm. Using the mean ± SD of triplicate cultures, a dose-response curve was automatically generated using nonlinear regression analysis. Three reference parameters were calculated (NCI algorithm) by automatic interpolation: GI_50_ = compound concentration that produces 50 % cell growth inhibition, as compared to control cultures; TGI = total cell growth inhibition (cytostatic effect), as compared to control cultures, and LC_50_ = compound concentration that produces 50 % net cell killing (cytotoxic effect).

### Mass spectra and structural elucidation

(+)-HRESIMS was performed on an Agilent 6230 time of flight LC/MS. NMR spectra were recorded on a Varian “Unity 500” spectrometer at 500/125 MHz (^1^H/^13^C). Chemical shifts were reported in ppm using residual CD_3_OD (d 3.31 for ^1^H and 49.0 for ^13^C) as internal reference. HMBC experiments were optimized for a ^3^*J*_CH_ of 8 Hz. ROESY spectra were measured with a mixing time of 500 ms. The structures were established by ^1^H- and ^13^C-NMR and two dimensional NMR experiments correlation spectroscopy (COSY), heteronuclear multiple quantum coherence (HMQC), heteronuclear multiple-bond correlation (HMBC).

### DNA manipulation and plasmids construction

The isolation and manipulation of DNA were carried out following standard general methods previously described for *E. coli* [70] and *Sreptomyces* [66]. PCR amplifications were conducted by using Herculase II Fusion polymerase (Agilent Technologies) with a touchdown PCR procedure. The termocycler (SureCycler 8800, Agilent Technologies) was programmed as follow: initial denaturation at 99.9 °C for 4 min; 20 cycles of 99.9 °C for 20 s, 65–45 °C touchdown for 20 s and 72 °C for tx (20 s/kb) min followed by 10 cycles of 99.9 °C for 20 s, 60 °C for 20 s and 72 °C for tx (20 s/kb) min. Final extension was performed at 72 °C for 3 min. PCR products of the expected size were gel-purified and sequenced.

A detailed description of the construction of plasmids used in this work can be found in Additional file 3: Methods S1. Plasmids for inactivation of genes *gonP1* and *gonP8* were constructed in the conjugative plasmid pOJ260 [71], which lacks the capacity to replicate in *Streptomyces* and carries the *aac*(*3*)*IV* gene marker that confers resistance to apramycin (Apm^R^). To achieve the single deletion of genes *gonM4*, *gonMT*, *gonSL*, *gonS1*, *gonS2*, *gonCP*, *gonMR*, *gonL1*, *orf9*, *orf10*, *orf11* and *orf13*, DNA sequences flanking the target genes were amplified with the primer pairs designated in Additional file 3: Table S1 and cloned at both sides of the *aac*(*3*)*IV* gene in plasmid pEFBA-oriT [72]. The hygromycin B resistance (Hyg^R^) gene marker, *hyg*, was then extracted from plasmid pLHyg [73] and introduced in the deletion plasmids (Additional file 3: Methods S1). Gene *hyg* allows recognizing clones in which a complete gene replacement by a double cross-over has taken place (Hyg^S^ Apm^R^) from those in which a single cross-over event has integrated the deletion plasmid into the chromosome (Hyg^R^ Apm^R^). A suitable plasmid backbone to accomplish the complementation of Apm^R^ mutants was constructed as follows. The integrative plasmid pSETec [68], which harbors the constitutive ermE*p promoter, was digested with NcoI. A 1.6 Kb fragment containing *hyg* was extracted from pLHyg by NheI/SpeI digestion. The linearized pSETec plasmid and the pLHyg NheI-SpeI fragment were then blunt-ended with the Klenow fragment of DNA polymerase I and ligated to afford plasmid pSETHe. Complementation plasmids were generated by inserting the target genes into the XbaI/EcoRV sites of pSETHe, under the transcriptional control of the ermE*p promoter (Additional file 3: Methods S1).

### Gene mutation and complementation by intergeneric conjugation

Plasmids pOJ-*gonP1* and pOJ-*gonP8*, pD-*gonM4*, pD-*gonMT*, pD-*gonSL*, pD-*gonS1*, pD-*gonS2*, pD-*gonCP*, pD-*gonMR*, pD-*gonL1*, pD-*orf9*, pD-*orf10*, pD-*orf11* and pD-*orf13* (Additional file 3: Methods S1) were transferred into *S. caniferus* GUA-06-05-006A by intergeneric conjugation to generate the mutant strains *gonP1*^−^, *gonP8*^−^, Δ*gonM4*, Δ*gonMT*, Δ*gonSL*, Δ*gonS1*, Δ*gonS2*, Δ*gonCP*, Δ*gonMR*, Δ*gonL1*, Δ5201, Δ5257, Δ5259 and Δ5261, respectively. A standard conjugation procedure [66] with minor modifications was followed. Briefly, *S. caniferus* GUA-06-05-006A mycelia fragments stored at −20 °C from a SMS culture (5 ml) supplemented with 10 mM MgCl_2_ were used as plasmid recipient. *E. coli* donor cells were grown to an absorbance of 0.6 at 600 nm in 2× TY media containing the appropriate antibiotics for plasmid selection and 10 mM MgCl_2_. Donor cells (5 × 10^−8^) were washed twice with 2× TY and mixed with mycelia in 2× TY to a final volume of 1 ml. The mating mixture was spread on mannitol-soya (MS) plates supplemented with 25 mM MgCl_2_ and incubated for 20 h at 30 °C. Plates were then overlayed with 3 ml of SNA containing nalidixic acid (0.6 mg) and apramycin (1.2 mg), and further incubated for 7–10 days. Exconjugants potentially harboring a gene deletion were transferred to MA plates with and without hygromycin B (100 µg/ml) and cultivated for 2 days. After several rounds of non-selective growth, Hyg^s^ recombinants were selected for gene replacement confirmation by PCR. Likewise, *gonP1*^−^ and *gonP8*^−^ exconjugants were refreshed in MA plates with nalidixic acid and apramycin and tested for correct gene inactivation by PCR (Additional file 3: Table S1**).** The resulting mutant strains were complemented with the conjugative plasmids pSETHe, pC-*gonP8*, pC-*gonM4*, pC-*gonMT*, pC-*gonSL*, pC-*gonS1*, pC-*gonS2*, pC-*gonCP*, pC-*gonMR* and pC-*gonL1* (Additional file 3: Methods S1), generating the strains *GUA*-pS, CP*gonP8*, CP*gonM4*, CP*gonMT*, CP*gonSL*, CP*gonS1*, CP*gonS2*, CP*gonCP*, CP*gonMR* and CP*gonL1*, respectively. Selection of exconjugants carrying the corresponding complementation plasmid was performed by overlying conjugation plates with 3 ml of SNA containing nalidixic acid (0.6 mg) and hygromycin (2 mg).

### Sequencing and bioinformatics analysis

The *S. caniferus* GUA-06-05-006A chromosome was sequenced at Lifesequencing Ltd., Valencia, Spain by Roche/454 pyrosequencing [74] on a Genome Sequencing FLX platform. The genome was assembled in the Newbler assembler package [75] version 2.8 by using default parameters. Identification of gene clusters for biosynthesis of secondary metabolites was performed by the antibiotics and secondary metabolite analysis shell: antiSMASH 3.0.4 [22]. Annotation of ORFs within the PM100117/18 biosynthesis gene cluster was based on database searching of the corresponding proteins carried out by BLAST algorithm [76] at the National Center for Biotechnology Information (NCBI). Additional sequence alignments were conducted by ClustalW2 [77] and EMBOSS needle [78] from the European Molecular Biology Laboratory (EMBL). Prediction of transmembrane domains was performed by TMHMM Server v. 2.0 (http://www.cbs.dtu.dk/services/TMHMM/).

The nucleotide sequence of the PM100117/18 gene cluster was deposited in GeneBank (accession numbers: LN997801 and LN997802).

## Additional files

10.1186/s12934-016-0443-5 Sequence alignment of the cluster PKS domains active sites. LD, loading domain; M1-M20, extension modules. Polyketide synthase domains are as follows: KS, ketosynthase; AT, acyltransferase; KR, ketoreductase; DH, dehydratase; ER, enoilreductase; ACP, acyl carrier protein; CAL, CoA-ligase. **Figure S2.** Genetic complementation of mutant strains. UPLC analysis of PM100117 (1) and PM100118 (2) production in strains *GUA*-pS, CP*gonP8*, CP*gonM4*, CP*gonMT*, CP*gonSL*, CP*gonS1*, CP*gonS2*, CP*gonCP*, CP*gonMR* and CP*gonL1*. **Figure S3.** Antibiotic activity test of compound 5. Diffusion disc assay against *Saccharomyces cerevisiae* and *Micrococcus luteus*. The length (cm) of the inhibition-growth halo is indicated by numbers and yellow lines. **Methods S2.** Antibiotic activity assay. Format: PDF.
10.1186/s12934-016-0443-5 ^1^H-NMR (CD_3_OD, 500 MHZ) spectra of PM100117 and PM100118. **Figure S5.** Mass and NMR spectra of compounds 3, 4, 5 and 6. ^1^H-NMR (CD_3_OD, 500 MHZ), ^1^H and MS spectra of compounds 3, 4, 5 and 6. ^13^C, gCOSY, TOCSY, gHSQC, gHMBC and ROESY spectra of compounds 3, 4 and 5. Format: PDF.
10.1186/s12934-016-0443-5 Plasmids construction. **Table S1.** Primers used in this work. Format: PDF.

## Abbreviations

2,6-DG: NDP-4-keto-2,6-dideoxy-d-glucose
3,5-EPI: 3,5-epimerization
ABC: ATP-binding cassette transporters
ACP: acyl-carrier-protein
Apm^R^: apramycin resistant phenotype
ARNT: aryl hydrocarbon receptor nuclear translocator protein
AT: acyltranferase
ATa: malonyl-CoA acyltranferase
ATp: methylmalonyl-CoA acyltranferase
ATP: adenosine triphosphate
C4-KR: C4-ketoreduction
CAL: CoA ligase
CoA: Coenzyme A
DH: dehydratase
DH6N: 1,4-dihydroxy 6-napthoic acid
ER: enoylreductase
HPLC: high-performance liquid chromatography
HTH: helix-turn-helix
Hyg^R^: hygromycin B resistant phenotype
Hyg^s^: hygromycin B sensitive phenotype
I: identity
KR: ketoreductase
KS and KSQ: ketosynthase
KSIII: 3-oxoacyl-ACP synthase III
LAL: large ATP binding members of the LuxR family
LC: liquid chromatography
LD: loading domain
M: module
MK: menaquinone
MS: mass spectrometry
NMR: nuclear magnetic resonance
NQ: naphthoquinone
NRPS: non-ribosomal peptide synthetase
ORF: open reading frame
PAS: PER-ARNT-SIM homologous
PER: *Drosophila* period protein
PKS: polyketide synthases
Rt: retention time
S: similarity
SIM: *Drosophila* single-minded protein
TFA: trifluoroacetic acid
UPLC: ultra-performance liquid chromatography
