# Supplementary material for: Characterization and engineering of the biosynthesis gene cluster for antitumor macrolides PM100117 and PM100118 from a marine actinobacteria: generation of a novel improved derivative
Source: Microb Cell Fact. 2016 Feb 22;15:44. doi: 10.1186/s12934-016-0443-5 (PMC4763440; doi:10.1186/s12934-016-0443-5)
Supplement: Supplementary file 1 — 10.1186/s12934-016-0443-5 Sequence alignment of the cluster PKS domains active sites. LD, loading domain; M1-M20, extension modules. Polyketide synthase domains are as follows: KS, ketosynthase; AT, acyltransferase; KR, ketoreductase; DH, dehydratase; ER, enoilreductase; ACP, acyl carrier protein; CAL, CoA-ligase. Figure S2. Genetic complementation of mutant strains. UPLC analysis of PM100117 (1) and PM100118 (2) production in strains GUA-pS, CPgonP8, CPgonM4, CPgonMT, CPgonSL, CPgonS1, CPgonS2, CPgonCP, CPgonMR and CPgonL1. Figure S3. Antibiotic activity test of compound 5. Diffusion disc assay against Saccharomyces cerevisiae and Micrococcus luteus. The length (cm) of the inhibition-growth halo is indicated by numbers and yellow lines. Methods S2. Antibiotic activity assay. Format: PDF. [file 12934_2016_443_MOESM1_ESM.pdf]

Figure S1. Sequence alignment of the cluster PKS domains active sites. LM, loading module; M1-M20, extension modules. Polyketide synthase domains are as follows: KS, ketosynthase; AT, acyltransferase; KR, ketoreductase; DH, dehydratase; ER, enoilreductase; ACP, acyl carrier protein; CAL, CoA-ligase.

ACPs

```
ACP-LM       DVVRDHAAAVLGHREPSAIHPGRTFKELGFDSVTSVELRNRLNRALTARLPSTALYNHPS
ACP-LM-P8    DLVRSAVAEVLGLGSAQEVAADRPLHQLGFTSLAAVALRDRLSAAIGRQLSAAIVYDYPT
ACP-M1       DLVREQIAAVLGHDKAAPVDSERPFKELGFDSLTAVELRHRLSRATGLRLPRTVVFDHPS
ACP-M2       DLVRTHASAVLGHSSTDAVRPARAFRELGFDSLTGVELRNRLSTATGSTLPATLVFDHPT
ACP-M3       GLVRKQVAAVLGYADTAEVETGRAFRELGFDSLTAVELRNRLNTAIGQKLPATVVFDHPN
ACP-M4       DAVRAAAAAVLGHDRPDAIPADRAFRDLGYDSLTAVELRNRLAEATGLRLPVTLVFDHPT
ACP-M5       DAVLGQAALVLGHTSAEAIDPERGFLELGFDSLTAVELRNRLTTVTGTRLPATLLFDYPE
ACP-M6       DLVRTEIAAVLGHSSAGRVDADRAFEDLGLDSLTALELRNALGSRTGLRLPATLVFDHPT
ACP-M7       DVVCTQVAAVLGHTSAAEVGDQQSFKDLGFDSLTAVELRNRLDTATGALLPATLVFDHPT
ACP-M8       GLVRRTTATVLGYPGSESVGTTRTFQELGTDSLTAVELRNALSAAVSLPLSPTLVFDHPT
ACP-M9       ELVRTQAALVLGHPGPEAIEPTRDFRGLGVDSLTAVELRNQLQAATGLPMPATLVFDHPS
ACP-M10      DLVRGHTAAVLGFRDRKAVEAVRAFRDLGFDSLTAVELRNGLNADTGMRLPATLVFDYPT
ACP-M11      DLVRSYAAGVLHYAGPAEVPPDRPFRDLGVDSLIAVELRNALSLACGITLSTTVVFDYPT
ACP-M12      ELVRTHAAAVLGYTTSADLEIGDTFRDLGFDSLTAVELRNGLNAETGLRLPATLVFDYPT
ACP-M13      TVVRTQVAHVLGYAGPDEVEPSRSFTDLGFTSLTAVELRNQLREATGMALPATLVFDYPT
ACP-M14      DVVRSYVATVLGYAGPSAVDPERAFSDLGFDSLSAVELRNGMSGITGLRLPATLVFDYPT
ACP-M15      DLVCTQVALVLGHGSAEDIEPDQAFKELGFDSLTAVELRNHLNAATQLRLPATLVFDYPT
ACP-M16      ELVRTYAAAVLGYYKPESLEARSAFRDLGFDSLTAVELRGRLNTETGLRLPATVVFDYPS
ACP-M17      ELVRAQAALVLGHTGADAVEPARDFRGLGIDSLTAVELRNRLGVATGLRLPATLVFDYPS
ACP-M18      TLVRTHVAGVLGHDSVDAIDPKRAFSELGFDSLMAVELRNRLGLATGTQLPATLLFDHPT
ACP-M19      ALVRAHVAAVLNHTDPDEVASHRAFRELGFDSLMAVELRNALSTAIGKRLPATLIFDHPT
ACP-M20      DLVRRQVAAVLGHGSPEEVEPDRGFPELGFDSLTAVELRNQLGTVTGLRLPPTLVFDFAT
ACP-M1-P8    DLVREQIAAVLGHSSADPVTATTELNGLGFDSLTSLTLRNALNSATKLVLAPGVAFDFTT
ACP-SL       ALVRRHVAAGLGQVSAEAIGPDQAFQDLGFDSLTALTVRNALNAATQLTLPPAALFDFAD
consensus    dlVrt iaaVLgh    d vdp raFreLGxDSLtavELRnrl  atglrlpaTlvFDhpt
```

DHs

```
DH-M5        HQPWLADHALDGTLLFPGTGFLELALQACERTGAGHVEELTLHAPLVVPEDG  ACTIVE
DH-M7        DQPWLADHALAGTAVVPGTAFVELAVLAGDRAGCPRVADLTLVSPLVLPEED  ACTIVE
DH-M15       THPWLGDHAVTGTVLFPGTGFLELAVRAADEVGCDRVEEFTIAAPLVLPERG  ACTIVE
DH-M17       AQPWLAEHRVSGSVLLPGTAFLELAVRAGDQVGCAQVEELTLEAPLVLPERG  ACTIVE
DH-M13       TQPWLAEHRIAGAIVVPSTALLELAVRAGDEVGCSHIRELALEAPLVLAEDG  ACTIVE?
DH-M20       THAWLADHAVLGRVILPATAYLDLAVSAGDRTGCDHLAELTLEAPLVLPEQG  ACTIVE
DH-SL        THDWLTHHRVADRTVVPGSALVELAVRAGDEVGCGLLETLSLEAPLVLPEQD  INACTIVE
DH-M9        DHPWLADHVVDGSLLVPGTAFLELALRAADQAGCDHVESLTIEAPLVLPAHE  ACTIVE
DH-M6        AHPWLADRAVDGRVQLPTSAYLDLALHLGAVAGCEHVGELGVDAPLVLSEQS  INACTIVE
DH-M1-P8     AYGWLARHDIDGVPLAPDPVLVELAVRAGDQVGFGKLAELTVQTPLVLPESG  ACTIVE?
consensus     hpwLxxHxxxGtllvPgtafleLavragd vGc hveeltleapLvlpe g
```

KRs

```
KR-M1        RLVLTGRRG...VSALG...DHGAYAAANA... ACTIVE
KR-M3        HLVLVGRRG...VGLLV...DHGAYAASNA... ACTIVE
KR-M2        HLVLTSRRG...VGEAR...GQGPYAAANA... ACTIVE
KR-M8        HLLLISRRG...VLDDG...GQANYAAANA... ACTIVE
KR-M19       HLLLVSRSG...VLDDG...GHANYAPGNA... ACTIVE
KR-M4        HLVLVSRRG...VLDDG...GQGSYAAANA... ACTIVE
KR-M5        GLLLASRRG...VLDDG...GQANYAAANA... ACTIVE
KR-M6        HLLLVGRRG...VLDDG...GQGNYAAGNT... ACTIVE
KR-M20       HLLLTSRRG...VVDDG...GQANYAAANA... ACTIVE
KR-M9        HLLLASRRG...VLDDG...GQGNYAAANA... ACTIVE
KR-M15       HLLLASRRG...VLDDG...GQANYAAANA... ACTIVE
KR-M17       RLVLSSRRG...VLDDG...GQGNYAAANA... ACTIVE
KR-M7        HLMLAGRRG...VLDDT...GQANYAAANA... ACTIVE
KR-M10       HVVLTSRRG...VVDSV...GQGAYAAANA... ACTIVE
KR-M16       HVVLTSRRG...VVDSV...GQGAYAAANA... ACTIVE
KR-M12       HVVLTSRRG...VVDSV...GQAAYAAANA... ACTIVE
KR-M11       RVVLTSRRG...VLDDG...GQANYAAANA... ACTIVE
KR-M18       HLVLAGRRG...VLDDG...GQANYAAANA... ACTIVE
KR-M1-P8     NLLLVSRQG...TVDDG...GQGNYAAANA... ACTIVE
KRx-M14      HLLLTSRRG...TLDDG...GLGNYAPGNA... ACTIVE
KR-SL        DLLVTSRRG...VTDNG...GQGNYAAANV... INACTIVE
consensus    hlvLxSRxG...vLDDg...GxGxxAxxxA...
```

ERs

```
ER-M17       VELGDVQAGESVLVHAAAGGVGMAAVQLARHLGAEVFGTASPGKWETLRSSGLNEAHIAS
ER-M9        VDLGGLEAGESVLVHAGAGGVGMAAVQLARHLGAEVFATASPGKWATLRALDLDEAHIAS
ER-M15       VELGEVQAGESVLVHSAAGGVGMAAVQLARHLGAEVFGTASPGKWETLRGSGLDEAHIAS
ER-M7        AELGDLQAGESVLVHAAAGGVGMAAVQVARHLGAEVFGTASPGKWDTLRELGLDEARIAS
consensus    veLGdvqAGESVLxHxxxGGVGxxAxxxARHLGAEVFgTASPGKWeTLRa gLdEAhIAS
```

KSs

```
KSQ-LM       GPSMTLDTAQSSSLVA
KS-M1        GPAVTVDTACSSSLVA
KS-M2        GPAVTVDTACSSSLVA
KS-M3        GPAVTVDTMCSSSLVA
KS-M4        GPAITLDTGCSSSLVS
KS-M5        GPAVTVDTACSSSLVA
KS-M6        GPAVTVDTACSSSLVA
KS-M7        GPAVTVDTACSSSLVA
KS-M8        GPAVTVDTACSSSLVT
KS-M9        GPALTVDTACSSSLVA
KS-M10       GPAVTVDTACSASLVA
KS-M11       GPAVTVDTACSSSLVA
KS-M12       GPAVTVDTACSSSLVA
KS-M13       GPAVTVDTACSSSLVA
KS-M14       GPAVSVDTACSSSLVA
KS-M15       GPAVTVDTACSASLVA
KS-M16       GPAVTVDTACSSSLVA
KS-M17       GPAVTVDTACSSSLVA
KS-M18       GPAVTVDTACSSSLVA
KS-M19       GPAVTVDTACSSSLVA
KS-M20       GPAVTVDTACSSSLVA
KS-M1-P8     GPAVTVDTACSSSLVA
consensus    GPavtvDTacSsSLVa
```

ATa

```
ATa-M5      RTVYTQ...GHSIGE...HAFH
ATa-M6      RTGFTQ...GHSIGE...HAFH
ATa-M7      RTEYTQ...GHSIGE...HAFH
ATa-M8      RTGFTQ...GHSIGE...HAFH
ATa-M9      RTEFTQ...GHSIGE...HAFH
ATa-M10     RTEFTQ...GHSVGE...HAFH
ATa-M11     RTEFAQ...GHSVGE...HAFH
ATa-M12     RTEFTQ...GHSIGE...HAFH
ATa-M13     QTGFTQ...GHSIGE...HAFH
ATa-M15     QTGFTQ...GHSIGE...HAFH
ATa-M16     RTAFTQ...GHSIGE...HAFH
ATa-M17     RTGFTQ...GHSIGE...HAFH
ATa-M18     RTAYTQ...GHSIGE...HAFH
ATp-M19     RVDILQ...GHSQGE...YASH
ATa-M20     RTGFTQ...GHSIGE...HAFH
consensus   rtgftQ...GHSiGE...hAfH
```

ATp

```
ATp-LM      RVDVVQ...GHSQGE...YASH
ATp-M1      RVDVVQ...GHSQGE...YASH
ATp-M2      RVDVVQ...GHSQGE...YASH
ATp-M3      RVDVVQ...GHSQGE...YASH
ATp-M4      RVDVVQ...GHSQGE...YASH
ATp-M14     RVDVIQ...GHSQGE...YGSH
ATp-M19     RVDILQ...GHSQGE...YASH
ATp-M1-P8   RVDVVQ...GHSQGE...YASH
consensus   RVDvvQ...GHSQGE...YaSH
```

CAL-domains (CoA-ligase) 1 Minowa substrate specificity prediction: AHBA

```
CAL-LD-P8     1 VSDTLRNELIRPLHELLSHHARRSPEKTAFRDAQRSVTYGELEERTRRLAGHLADLGVAR
CAL-SL        1 ----MRTELMRPLDELLLRHAGQRADETAFSDARRSLTYAALEQRTARLAGHLADLGLDR
consensus         vsdtlR ELiRPL ELL hHA    e TAF DA RSvTYg LE RT RLAGHLADLGv R

CAL-LD-P8    61 GDRVLLRMGNRVEMVESYVAVARAGAVGVPLNPQSTDAEFAHHLDDSGAVLVISGAAQAE
CAL-SL       57 GGRAVLYLDDGVEMREGSLAVLRSAAVGVPLTPHLTDAELAHLLDDSGADVLITAPALAD
consensus         G R lL m   VEM E  vAV R gAVGVPL P  TDAE AH LDDSGA lvIsg A Ae

CAL-LD-P8   121 QVLGVTGATTWAGTVVVVGAAPEVPDGAMRFETLAITEPESAARDDLGLDESAWMLYTSG
CAL-SL      117 QVLRVL--PHRPGCTVVVTGDGAAPDGTERYETLAATDPLAPVRDGLGLDDTAWMLYTSG
consensus         QVL V ga    G  VVV a    PDG  RfETLA TeP    RD LGLDesAWMLYTSG

CAL-LD-P8   181 TTGRPKAVVSTQQASLWATASCSVPLFGLGPEDRVLWPMPLFHAVSHSVGFLGVLAVGAT
CAL-SL      175 TTGRPEGVPATQRTCLWSTAAGTAPLLGPWALDRVLWPLPLFQGLTQSICLVEVRAADAT
consensus   181 TTGRP aV  TQ   LW TA  s PL G    DRVLWPmPLF avs Sv  l V A  AT
```

Figure S2. Genetic complementation of mutant strains. UPLC analysis of PM100117 (1) and PM100118 (2) production in strains *GUA*-pS, CP*gonP8*, CP*gonM4*, CP*gonMT*, CP*gonSL*, CP*gonS1*, CP*gonS2*, CP*gonCP*, CP*gonMR* and CP*gonL1*.

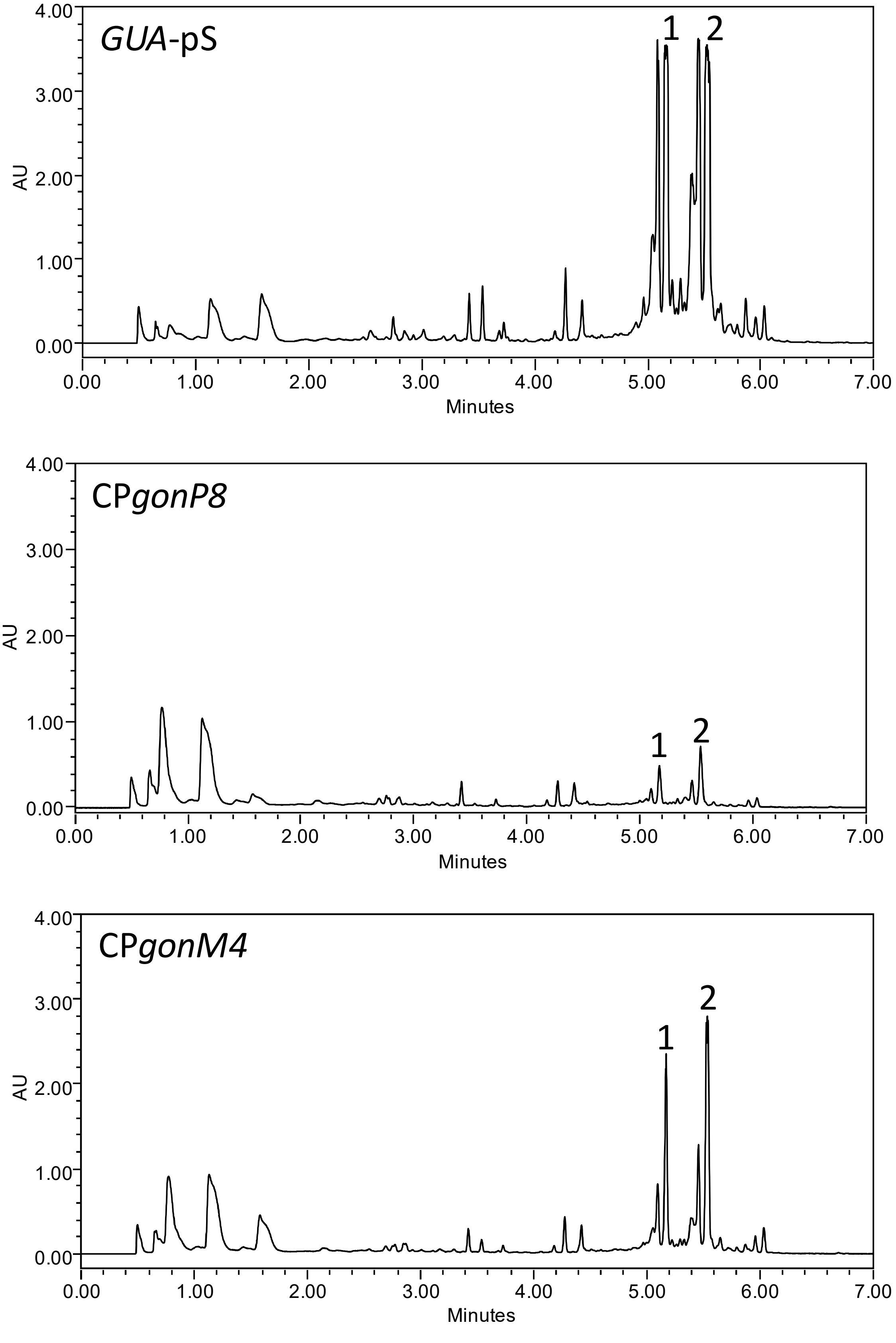

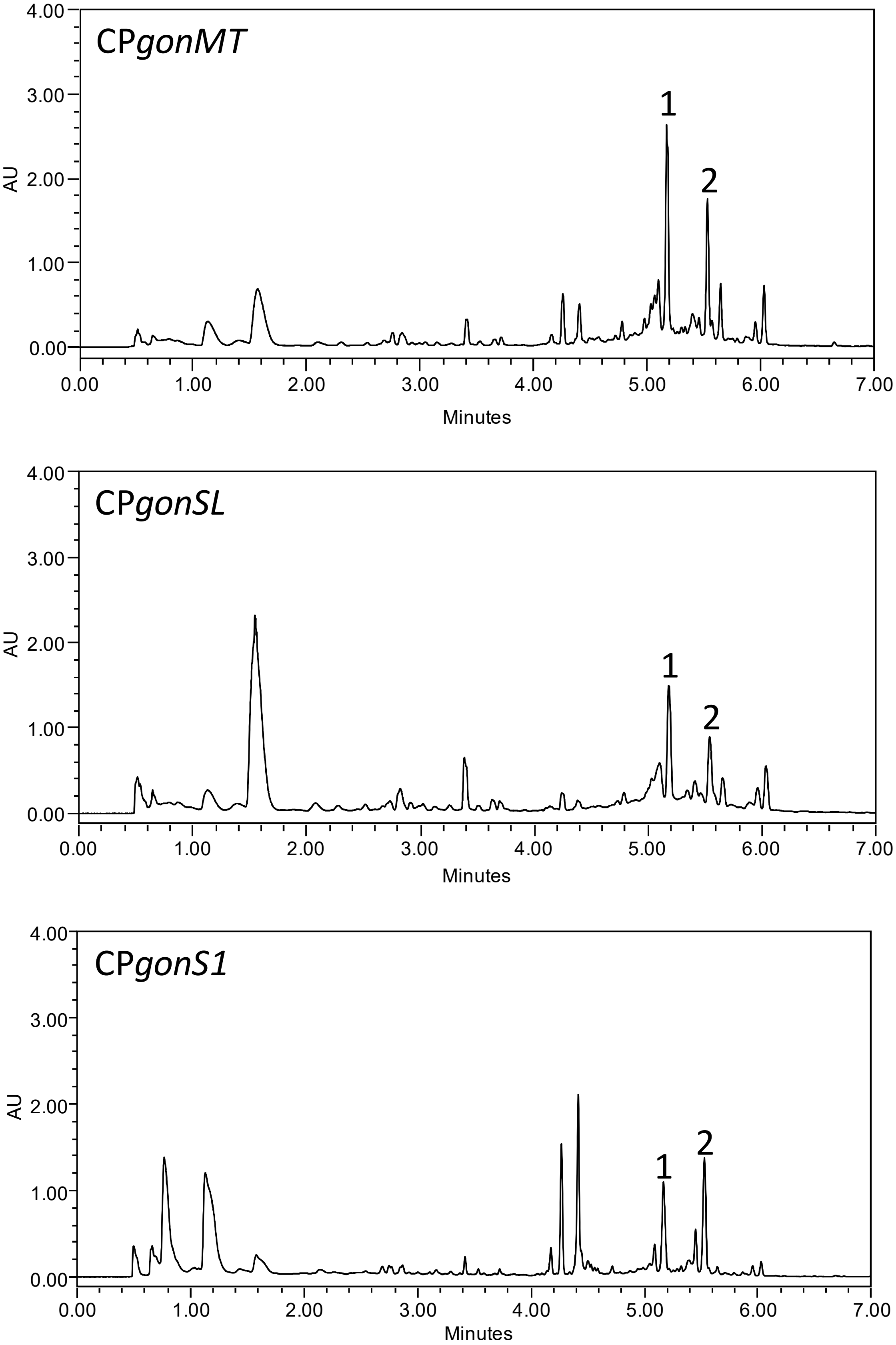

CPgonMT
CPgonSL
CPgonS1
AU
Minutes
1
2
0.00
1.00
2.00
3.00
4.00
5.00
6.00
7.00

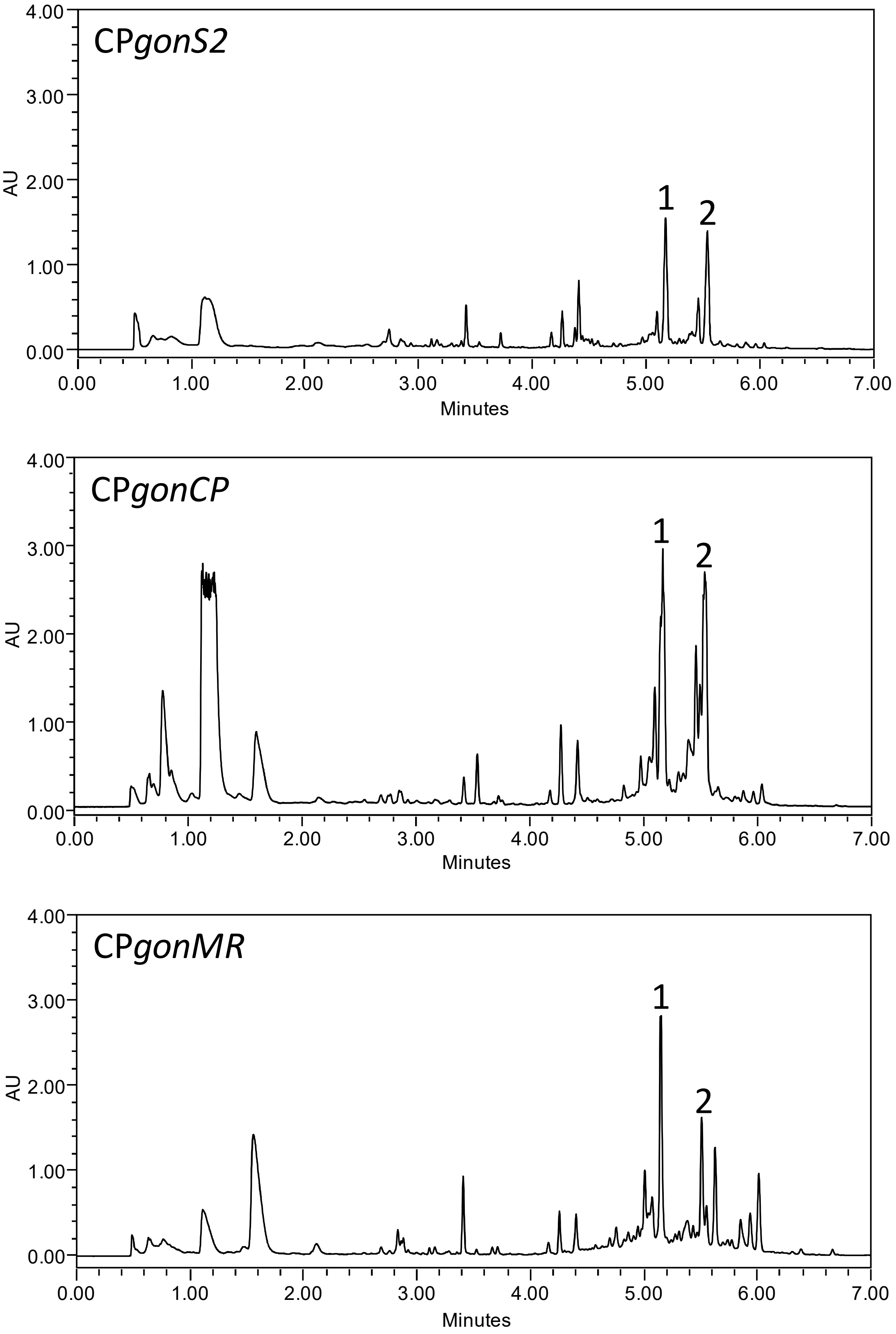

CPgonS2
CPgonCP
CPgonMR
AU
Minutes
1
2
0.00
1.00
2.00
3.00
4.00
5.00
6.00
7.00

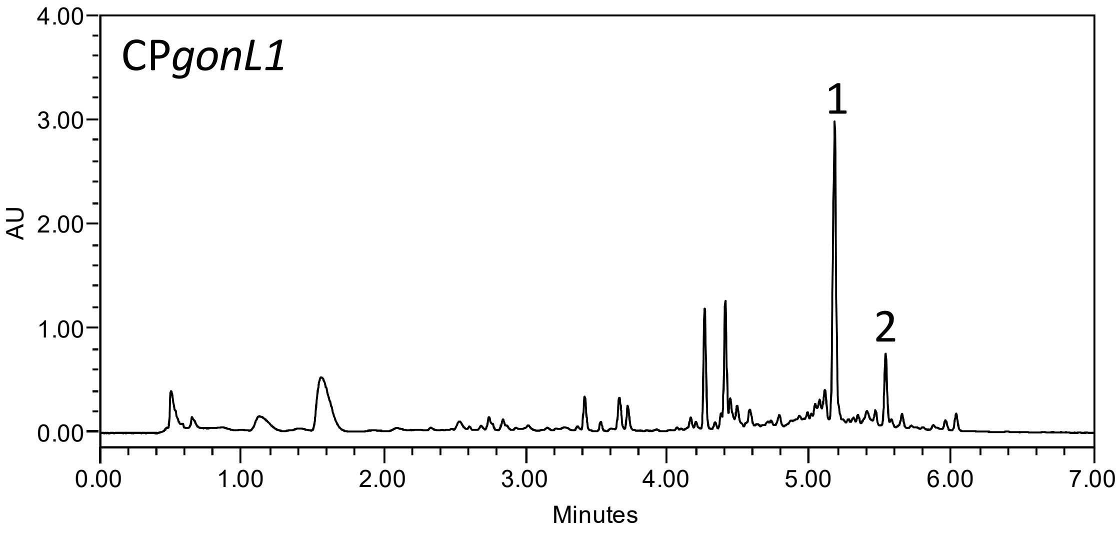

Figure S3. Antibiotic activity test of compound 5. Diffusion disc assay against *Saccharomyces cerevisiae* and *Micrococcus luteus*. The length (cm) of the inhibition-growth halo is indicated by numbers and yellow lines.

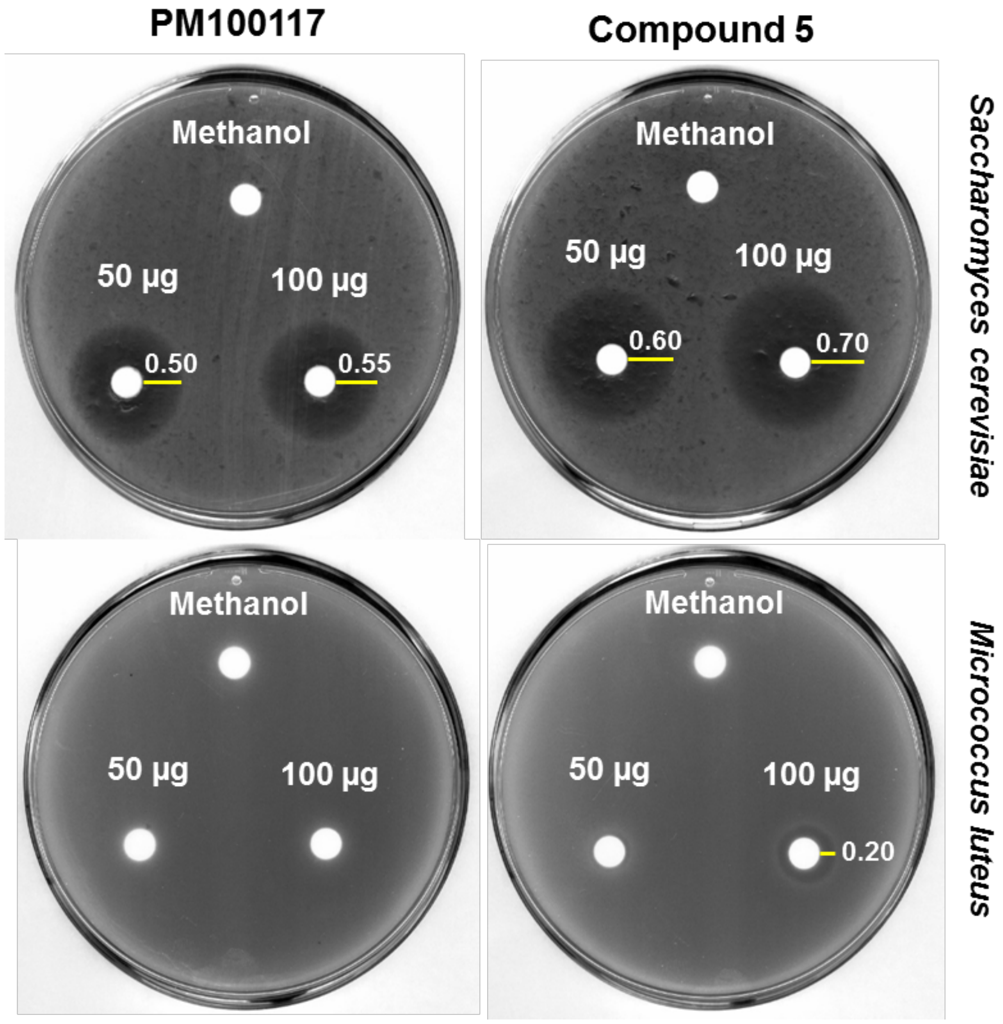

Methods S2. Antibiotic activity assay.

To perform antibiotic susceptibility tests, 20 ml of melted Sabouraud and TSA soft-agar (1%) medium were inoculated with 1 ml of a mild-exponential culture of *Saccharomyces cerevisiae* W303.1A and *Micrococcus luteus*, respectively. Soft-agar cultures were poured into two petri dishes and allowed to solidify at room temperature. Paper discs (9 mm) were impregnated with 50 µl of methanol or methanol extracts containing the indicated amounts of PM100117 or compound **5**, and then placed on the soft-agar dishes containing the test microorganisms. Plates were incubated at 30ºC and growth was recorded after 72h.
