# Supplementary material for: Characterization and engineering of the biosynthesis gene cluster for antitumor macrolides PM100117 and PM100118 from a marine actinobacteria: generation of a novel improved derivative
Source: Microb Cell Fact. 2016 Feb 22;15:44. doi: 10.1186/s12934-016-0443-5 (PMC4763440; doi:10.1186/s12934-016-0443-5)
Supplement: Supplementary file 2 — 10.1186/s12934-016-0443-5 1H-NMR (CD3OD, 500 MHZ) spectra of PM100117 and PM100118. Figure S5. Mass and NMR spectra of compounds 3, 4, 5 and 6. 1H-NMR (CD3OD, 500 MHZ), 1H and MS spectra of compounds 3, 4, 5 and 6. 13C, gCOSY, TOCSY, gHSQC, gHMBC and ROESY spectra of compounds 3, 4 and 5. Format: PDF. [file 12934_2016_443_MOESM2_ESM.pdf]

Figure S4. ^{1}**H-NMR (CD$_3$OD, 500 MHZ) spectra of PM100117 and PM100118.**

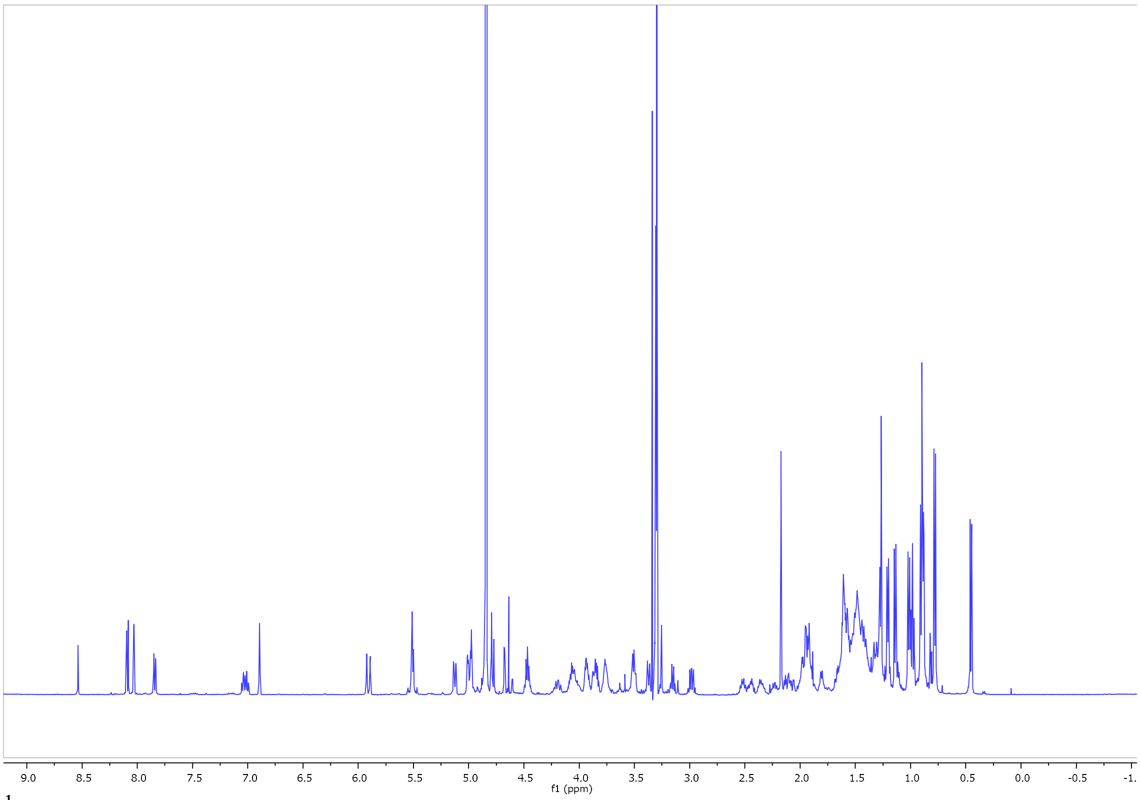

^{1}H-NMR (CD$_3$OD, 500 MHZ) of PM100117

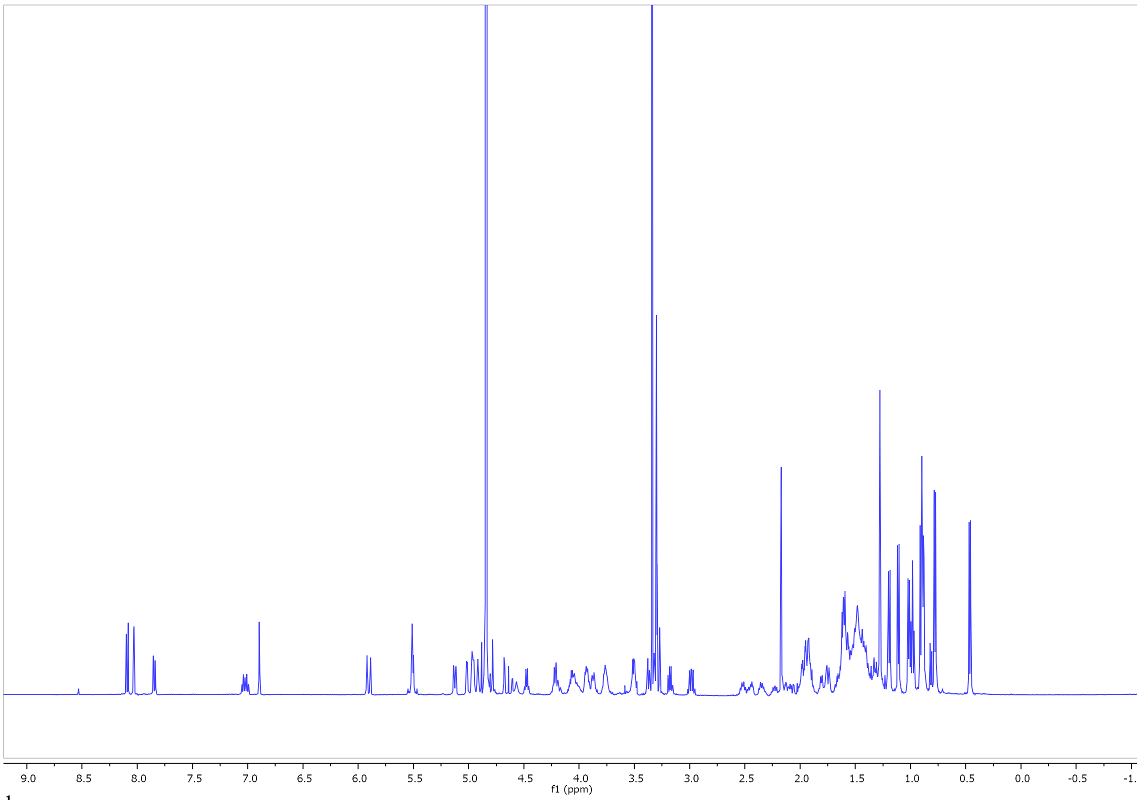

^{1}H-NMR (CD$_3$OD, 500 MHZ) of PM100118

Figure S5. Mass and NMR spectra of compounds 3, 4, 5 and 6. ^{1}H-NMR (CD_3OD, 500 MHZ), ^{1}H and MS spectra of compounds **3**, **4**, **5** and **6**. ^{13}C, gCOSY, TOCSY, gHSQC, gHMBC and ROESY spectra of compounds **3**, **4** and **5**.

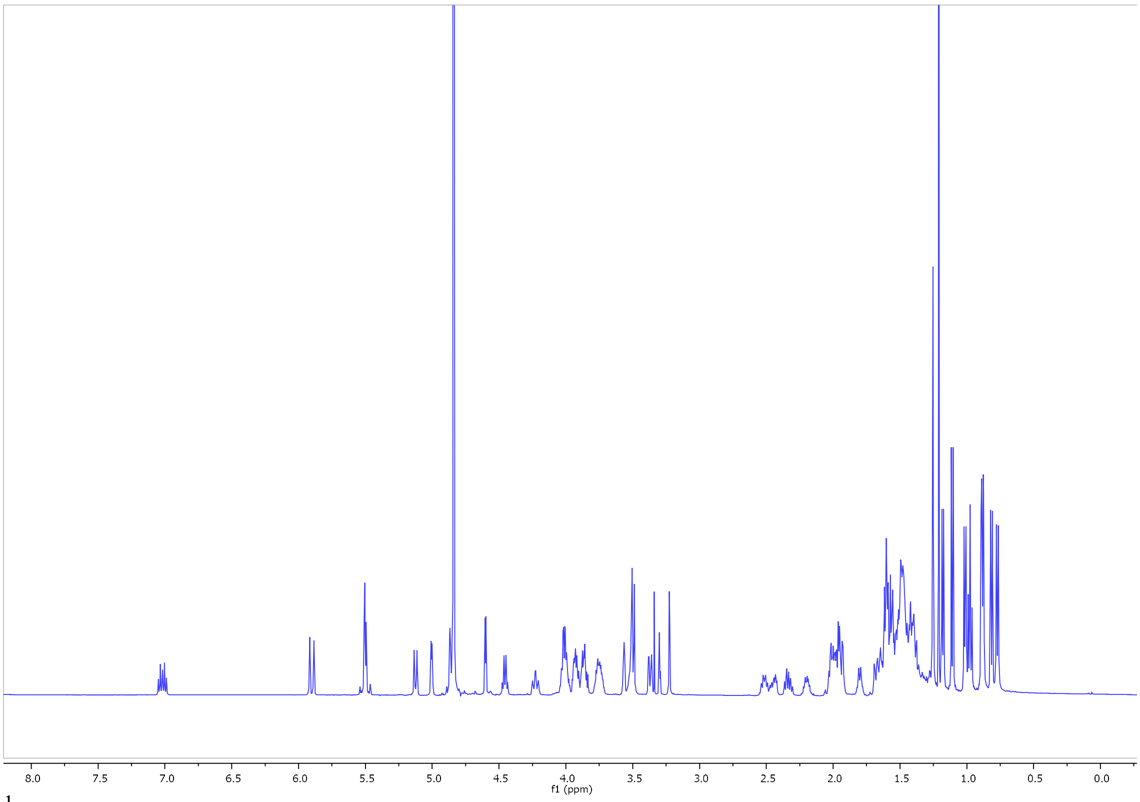

^{1}H-NMR (CD_3OD, 500 MHZ) of compound **3**

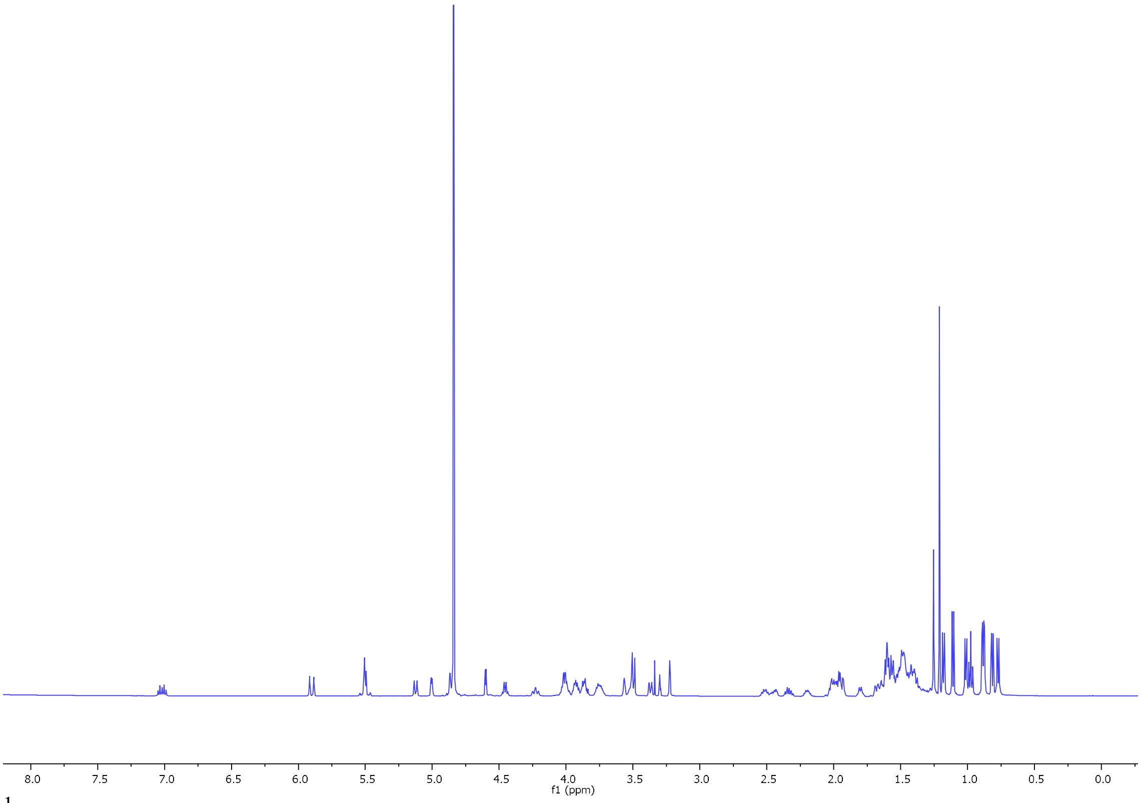

^{1}H spectrum of compound **3**

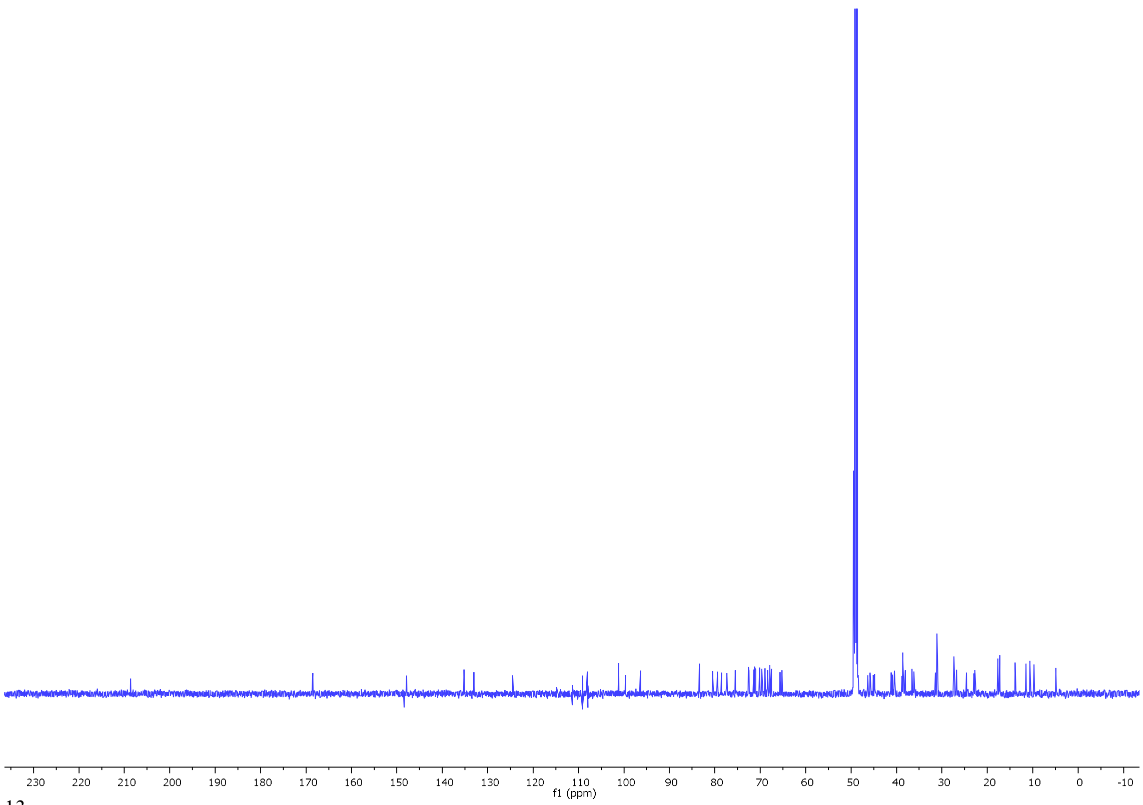

^{13}C spectrum of compound **3**

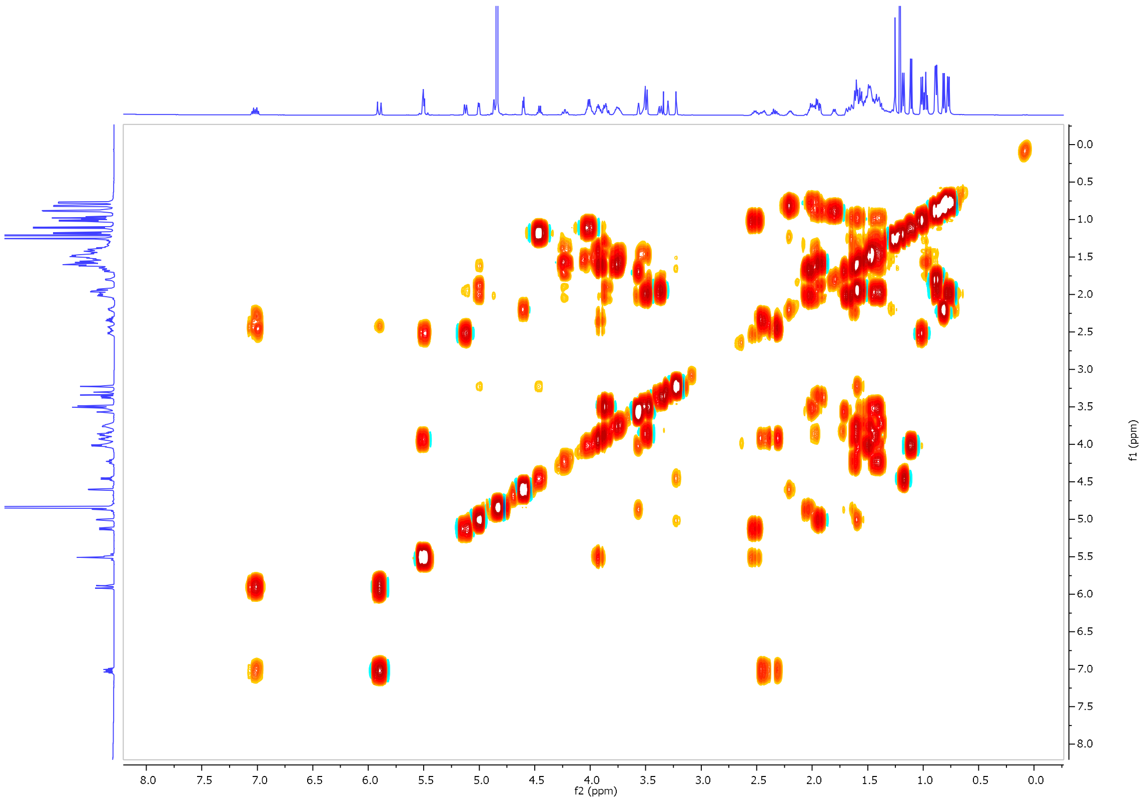

gCOSY spectrum of compound **3**

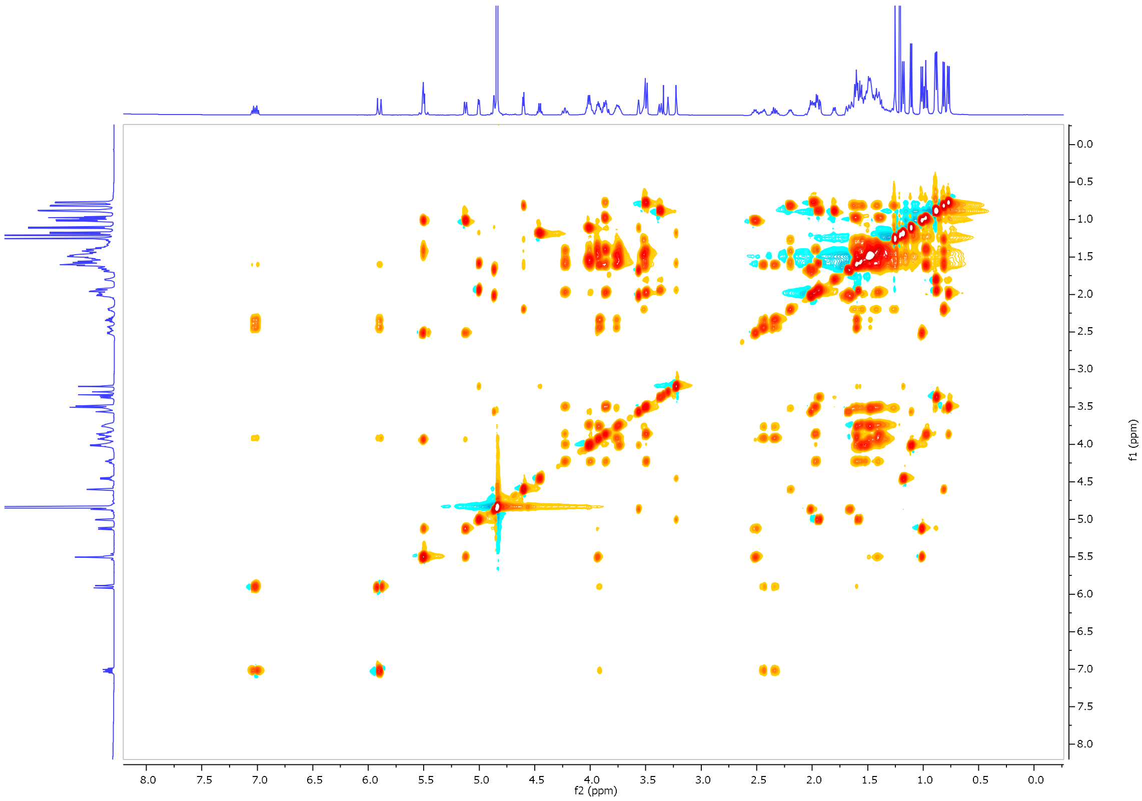

TOCSY spectrum of compound **3**

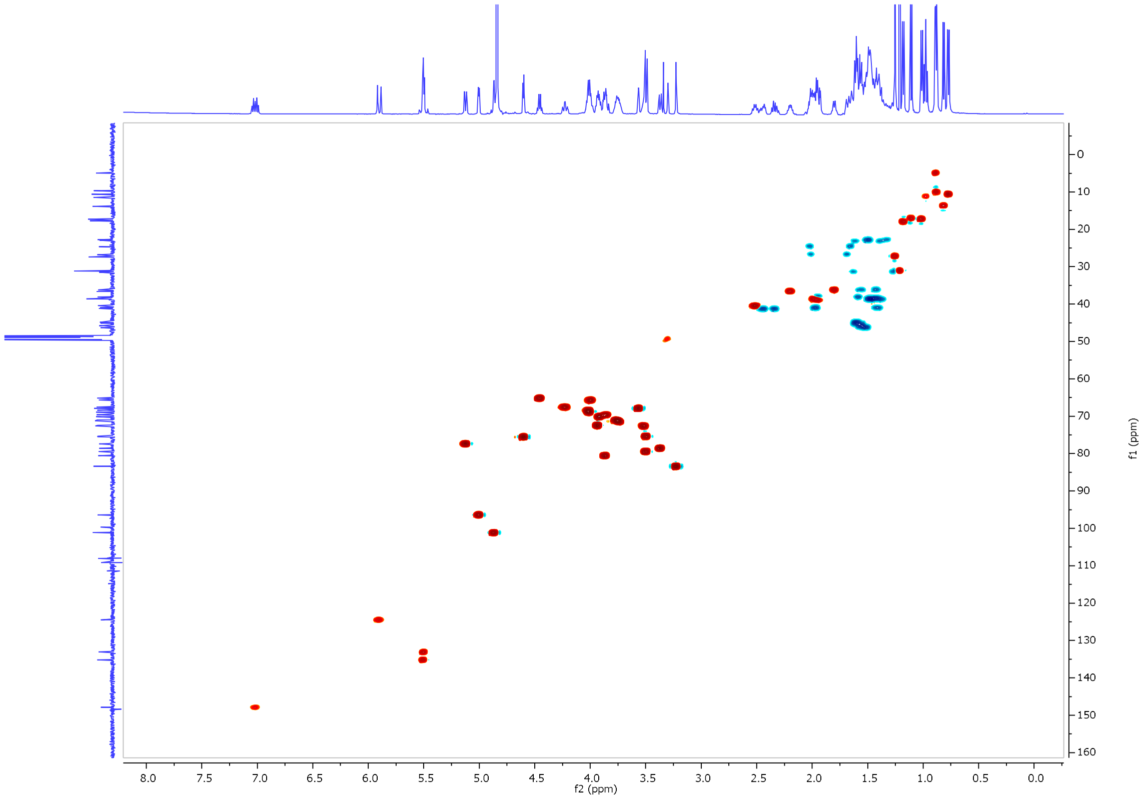

gHSQC spectrun of compound **3**

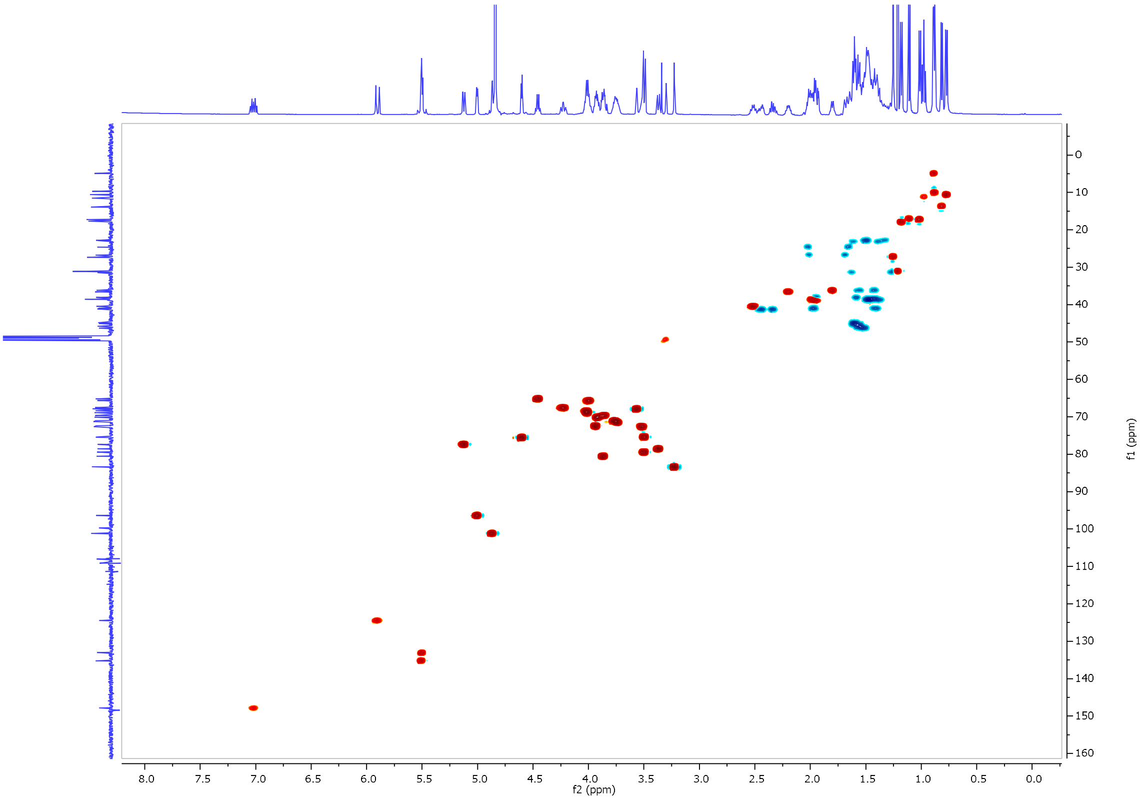

gHMBC spectrum of **3**

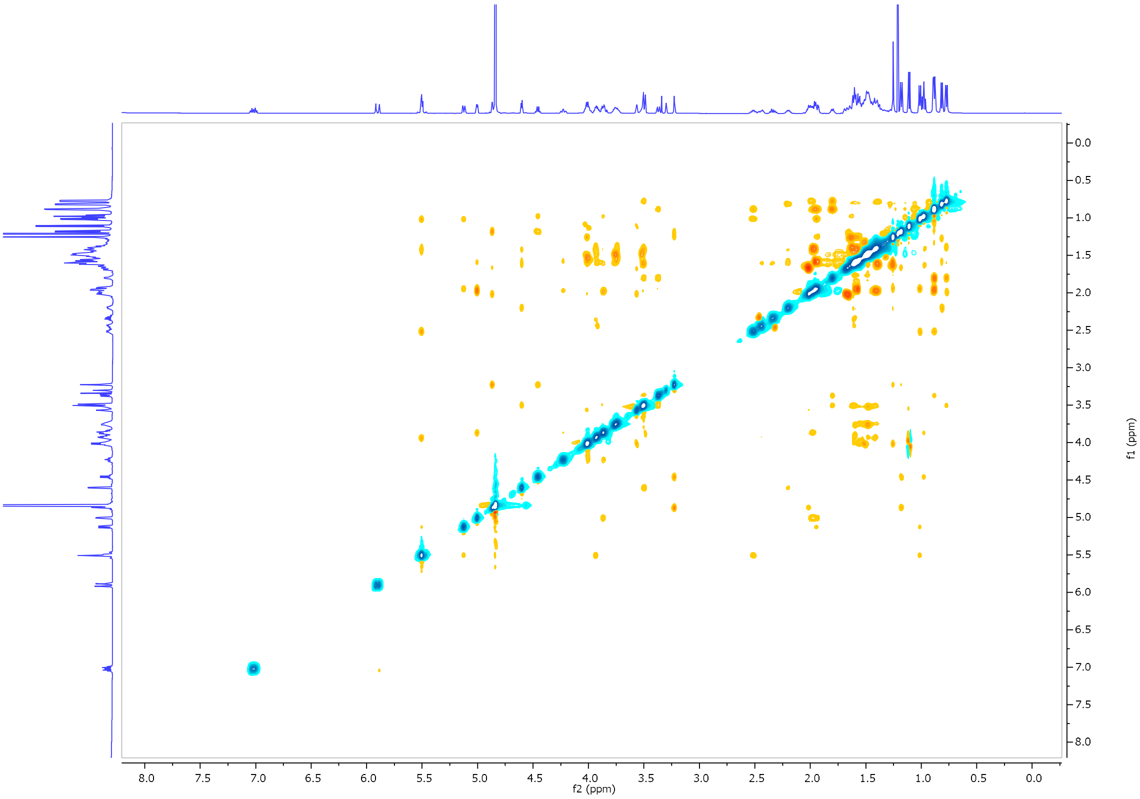

ROESY spectrum of **3**

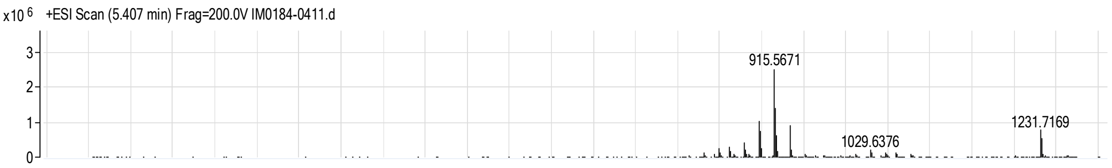

MS spectrum of **3**

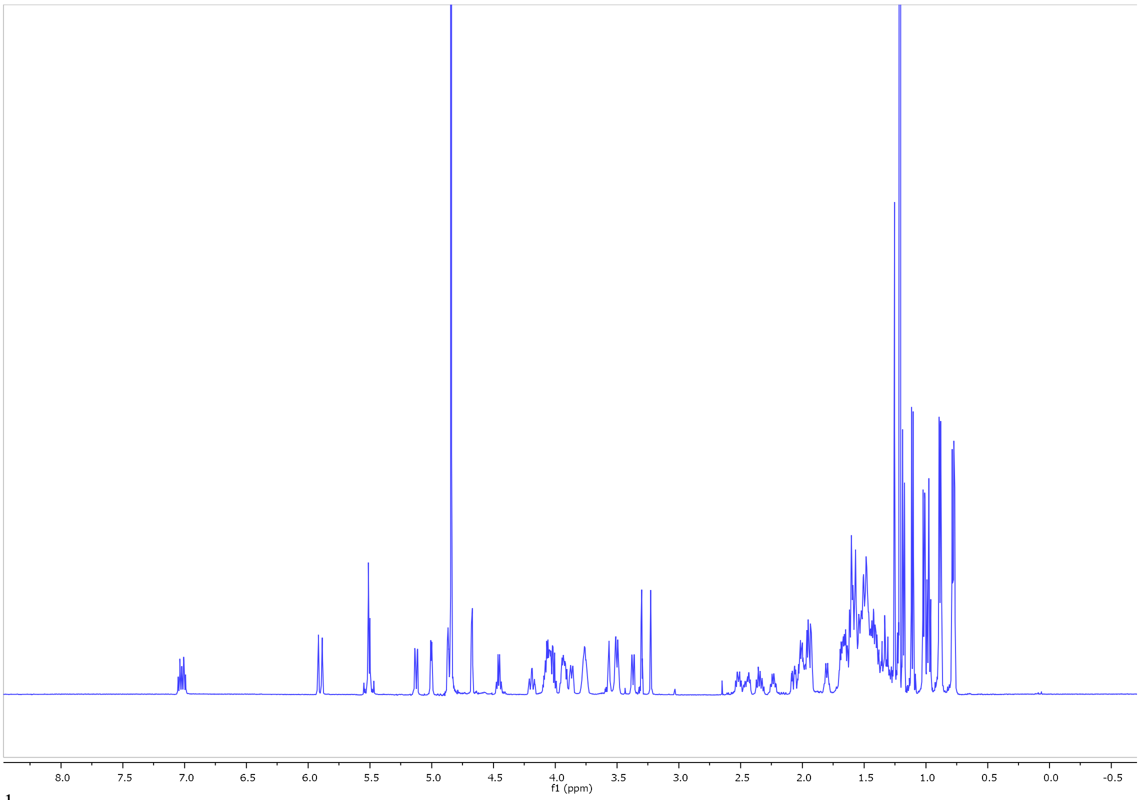

^{1}H-NMR (CD_3OD, 500 MHZ) of compound **4**

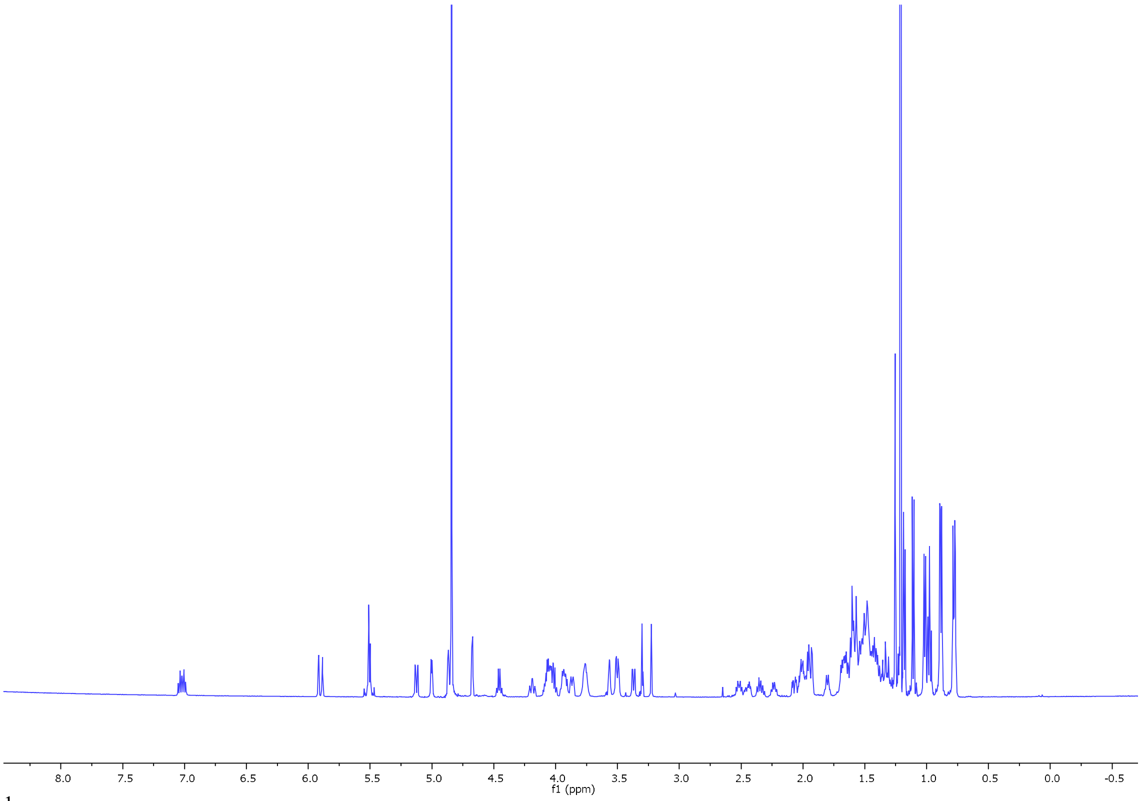

^{1}H spectrum of compound **4**

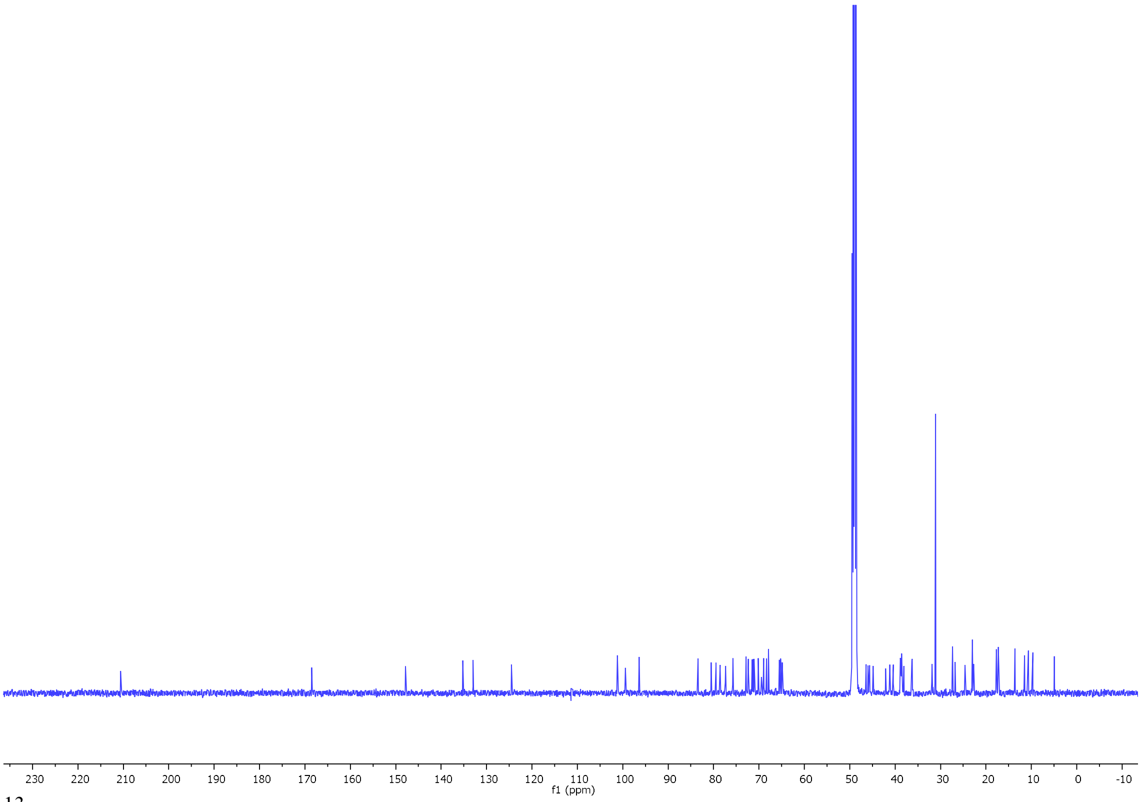

^{13}C spectrum of compound **4**

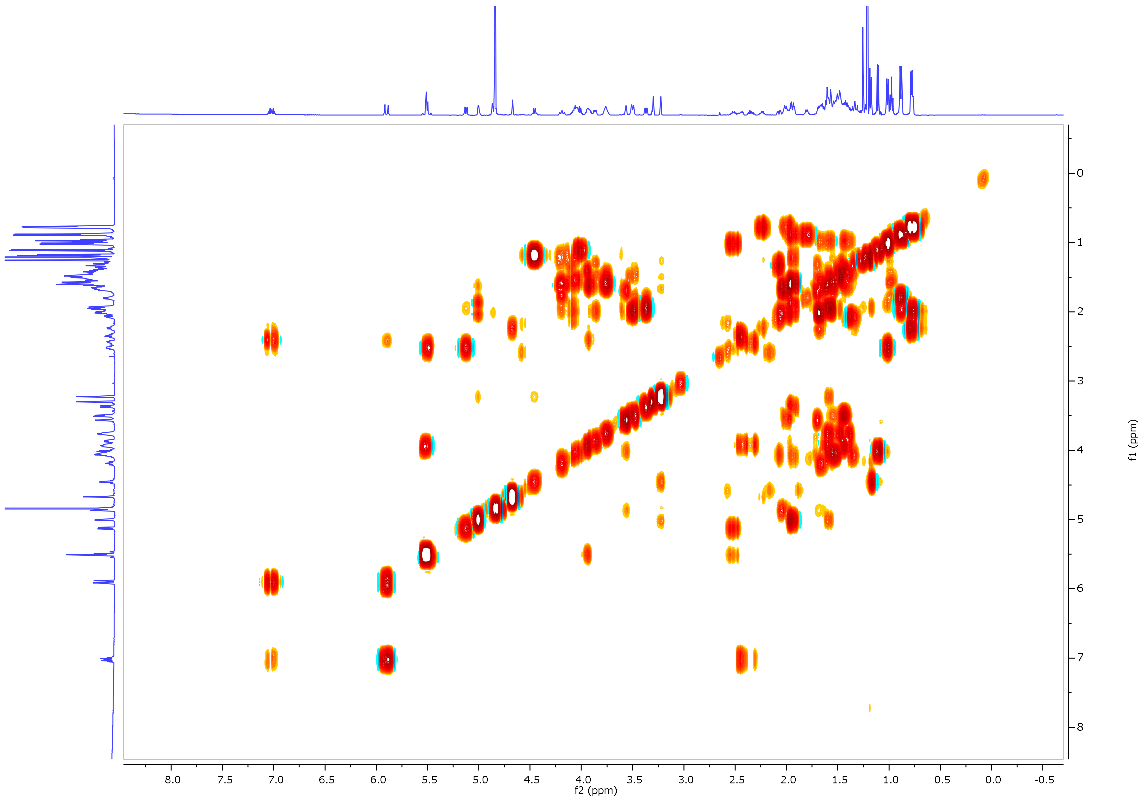

gCOSY spectrum of compound **4**

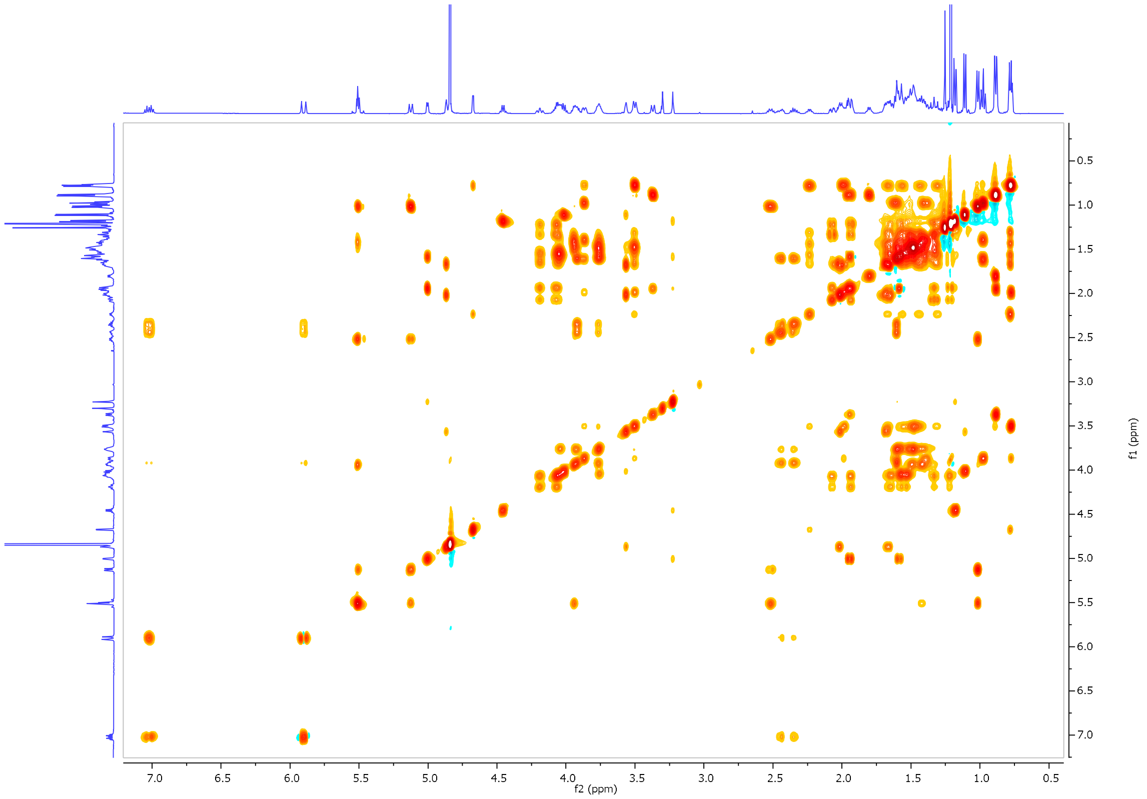

TOCSY spectrum of compound **4**

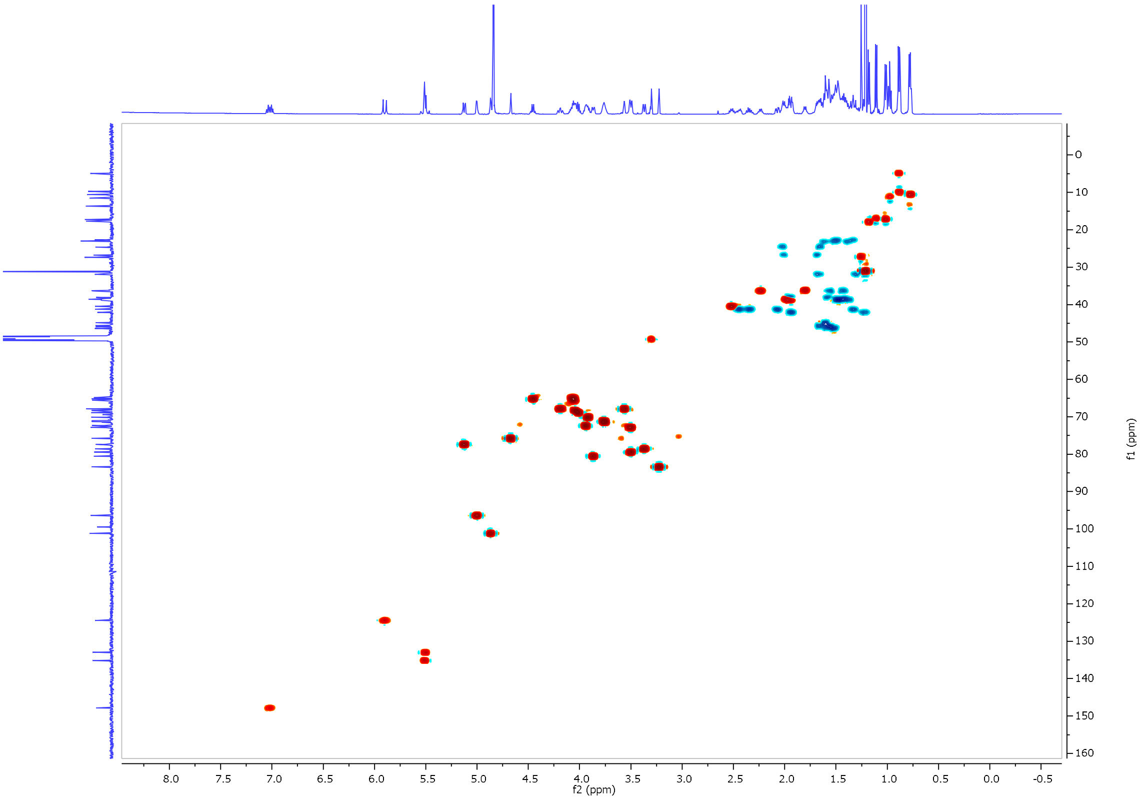

gHSQC spectrun of compound **4**

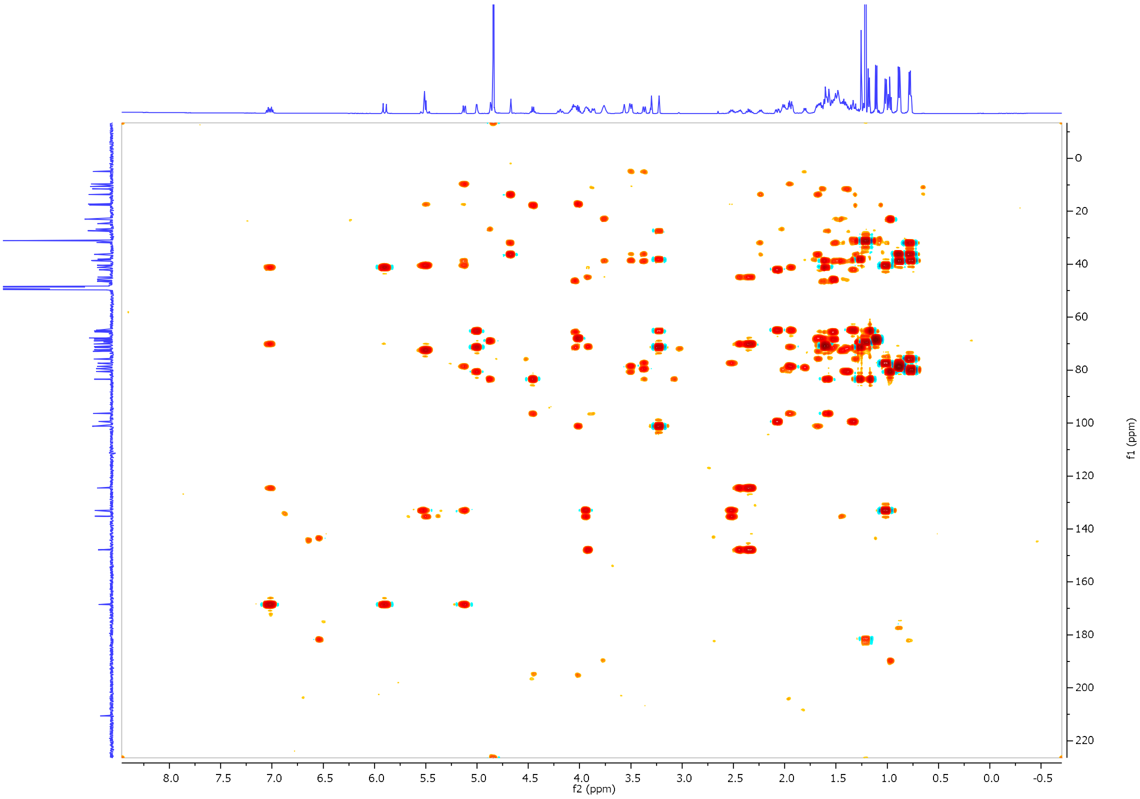

gHMBC spectrum of compound **4**

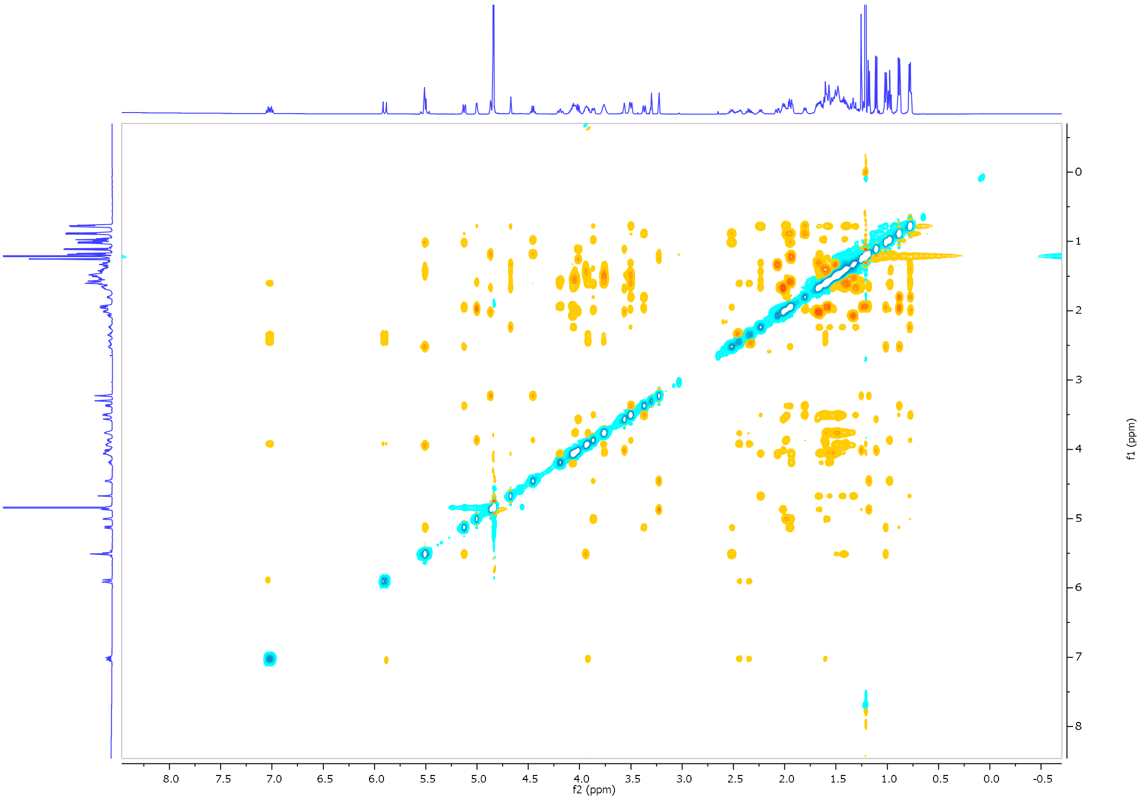

ROESY spectrum of compound **4**

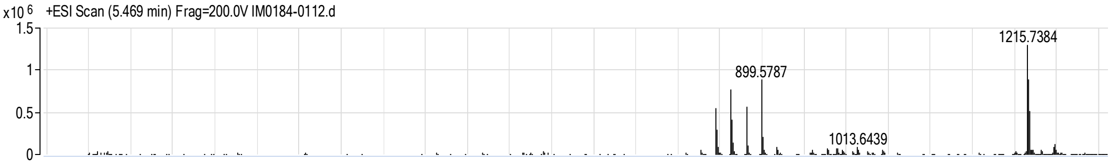

MS spectrum of compound **4**

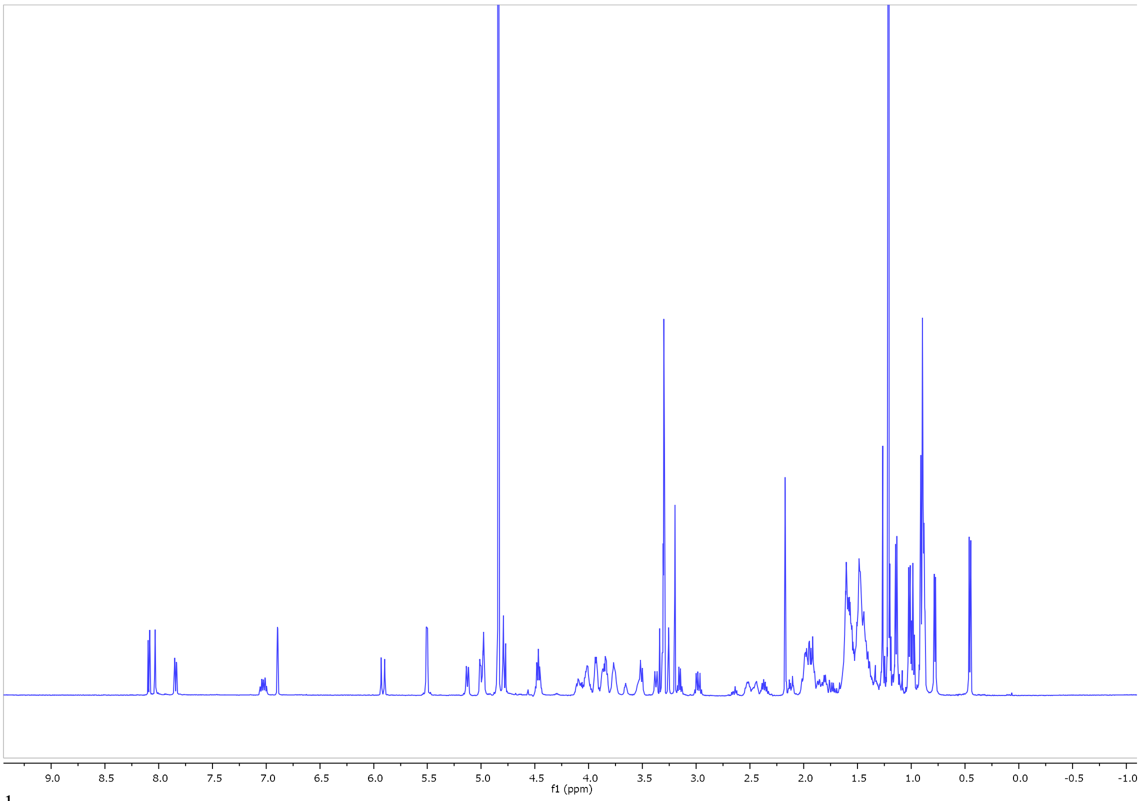

^{1}H-NMR (CD_3OD, 500 MHZ) of compound **5**

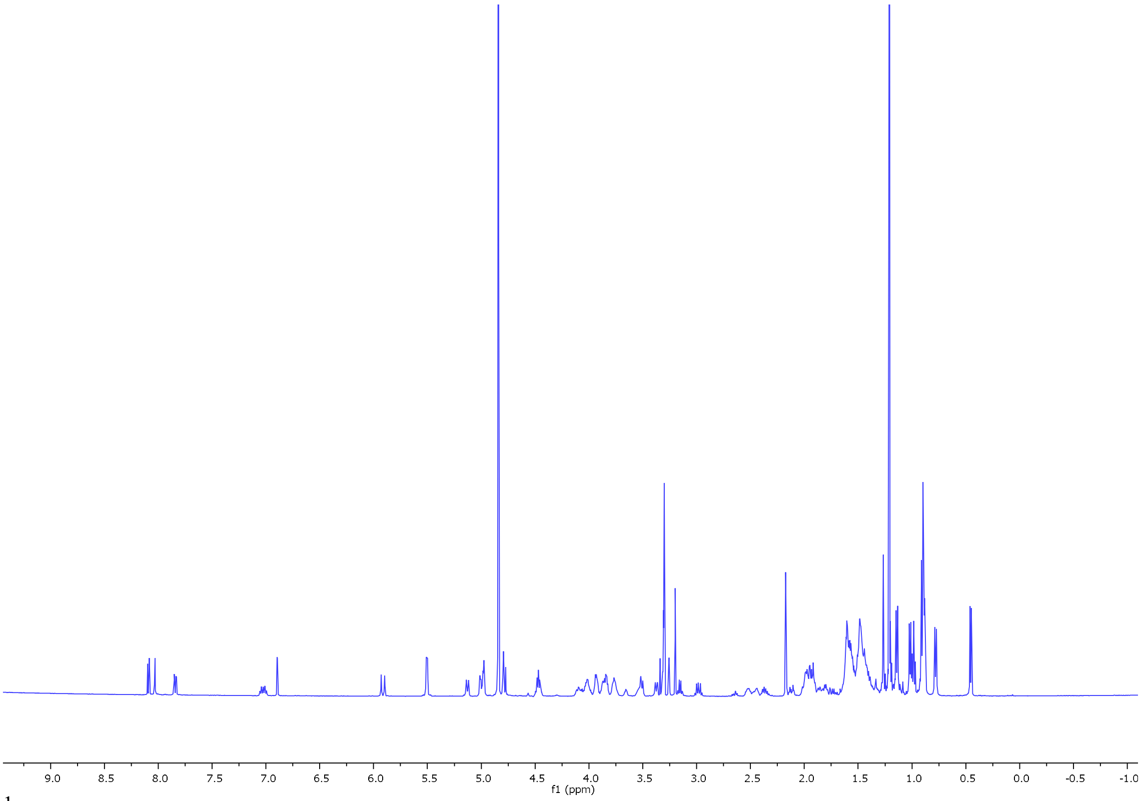

^{1}H spectrum of compound **5**

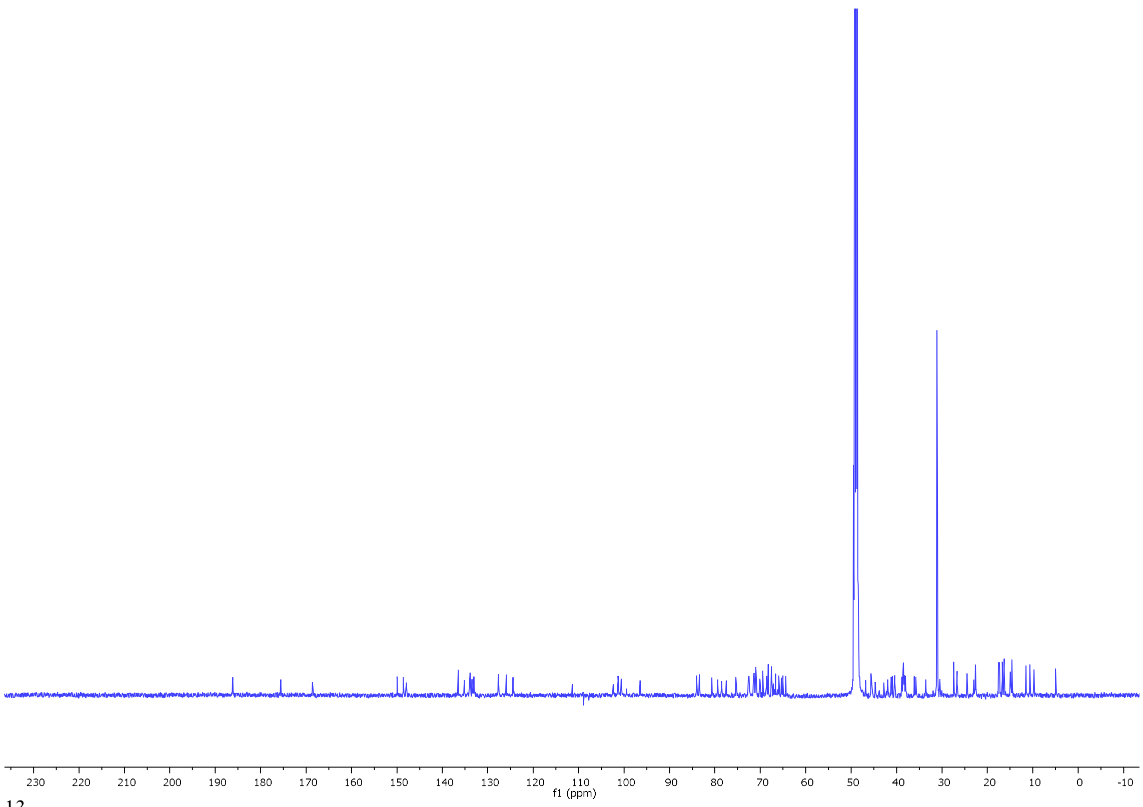

^{13}C spectrum of compound **5**

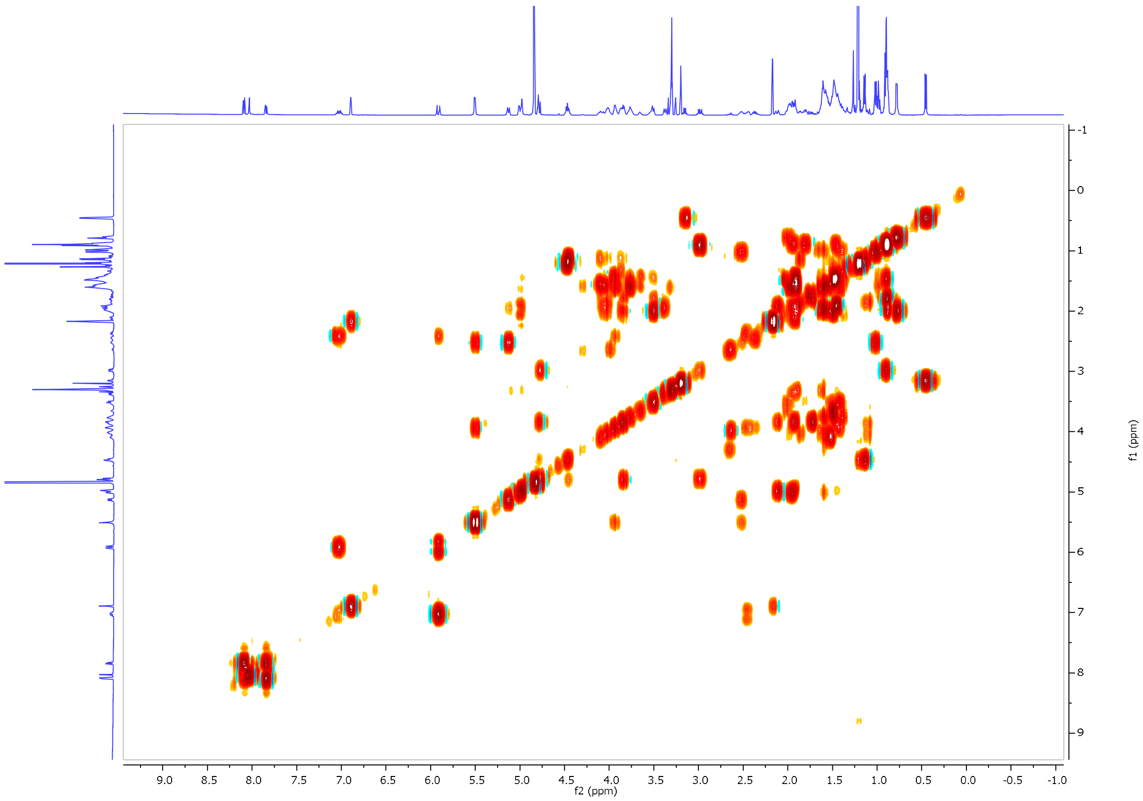

gCOSY spectrum of compound **5**

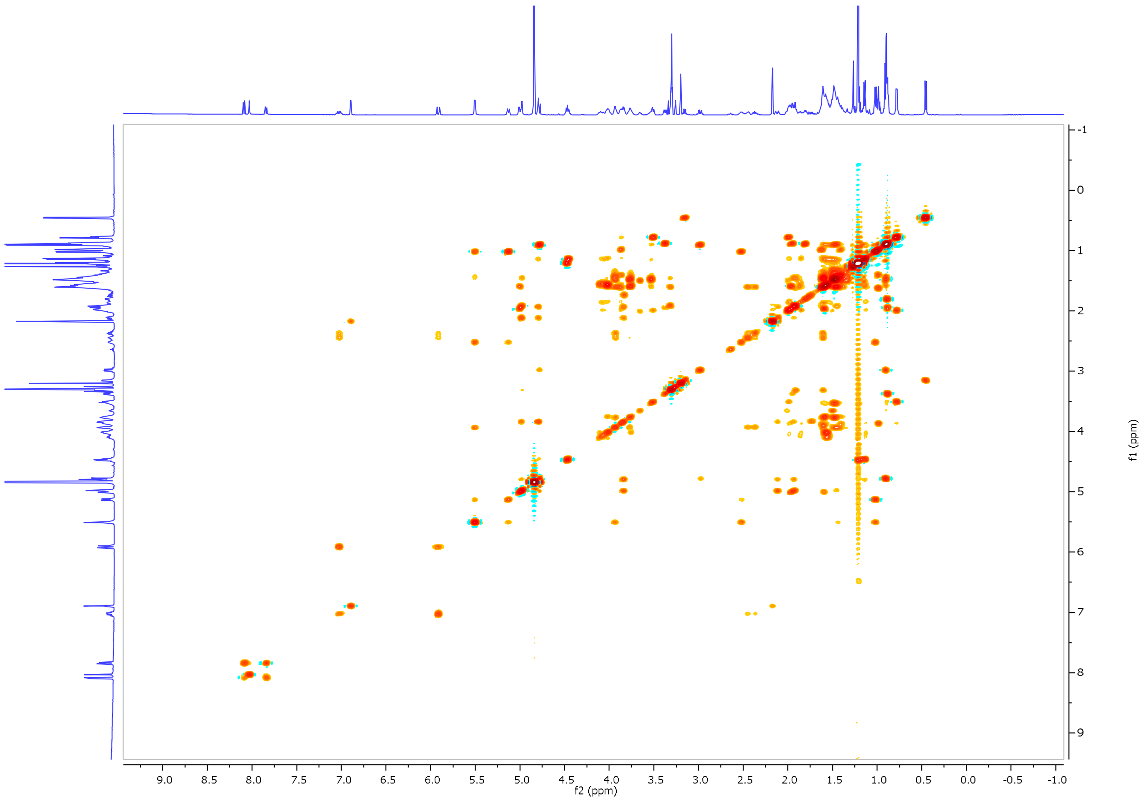

TOCSY spectrum of compound **5**

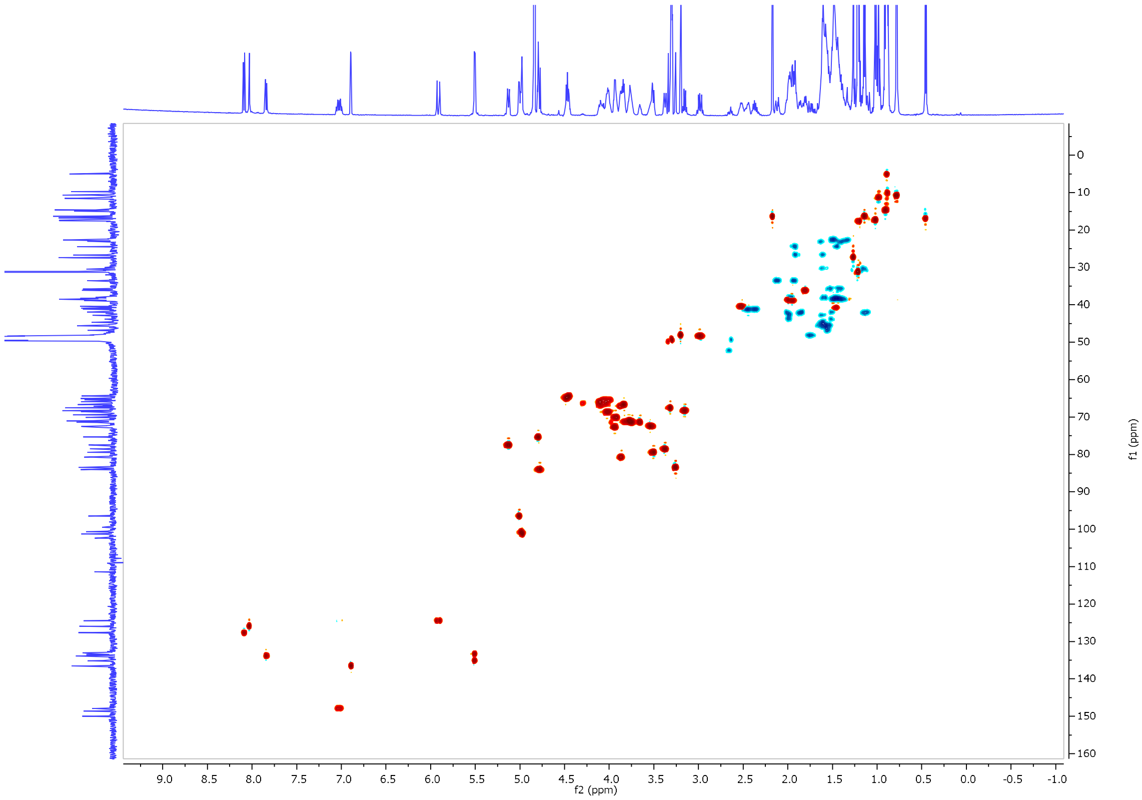

gHSQC spectrun of compound **5**

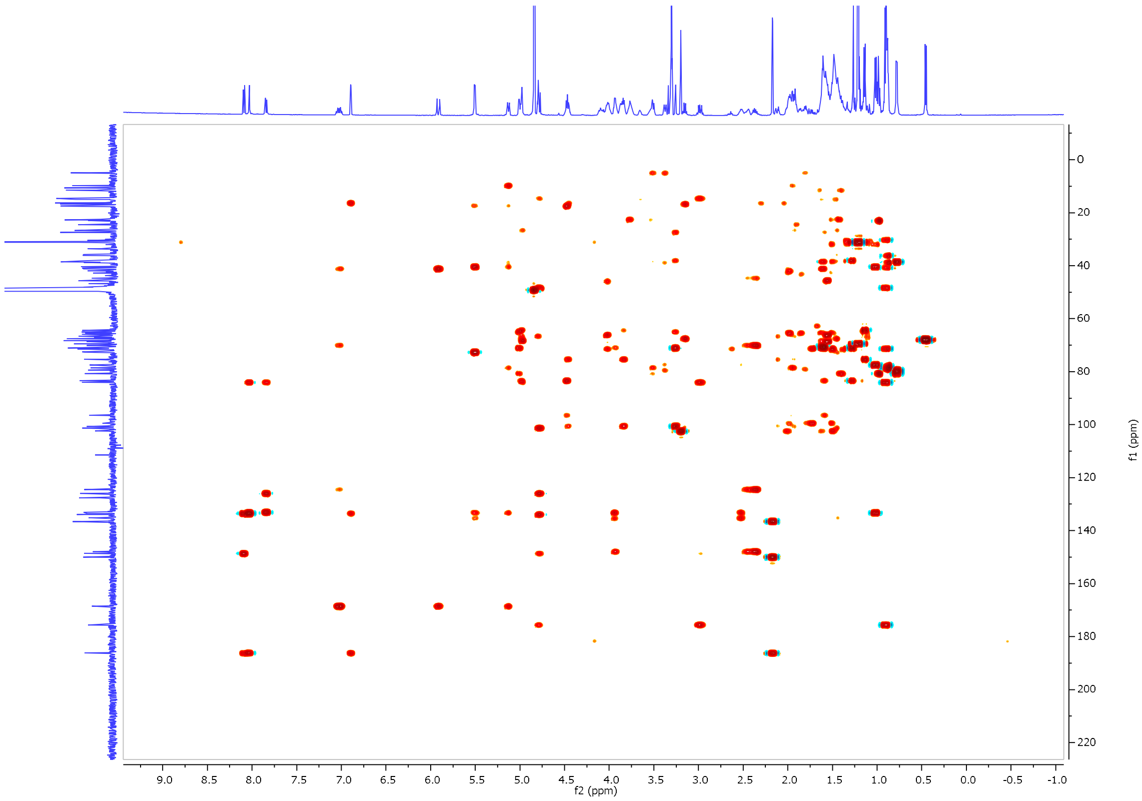

gHMBC spectrum of compound **5**

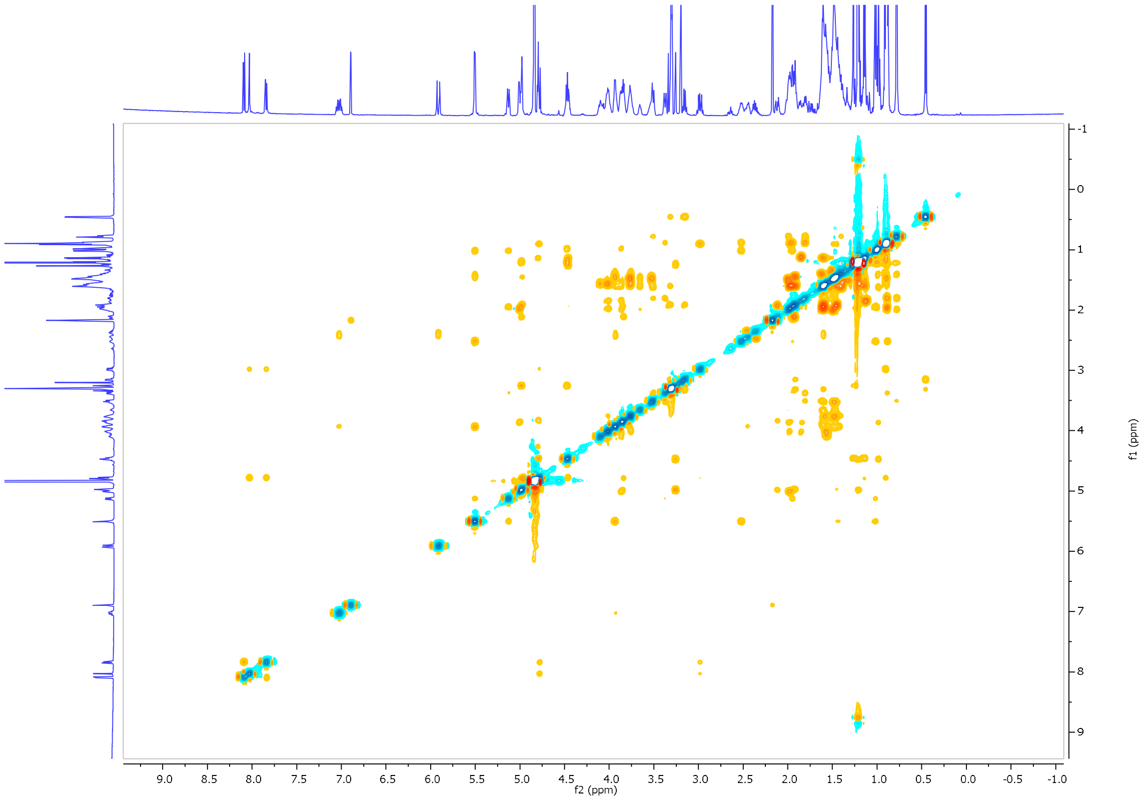

ROESY spectrum of compound **5**

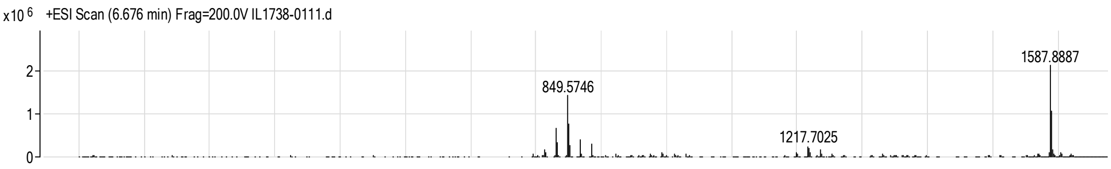

MS spectrum of compound **5**

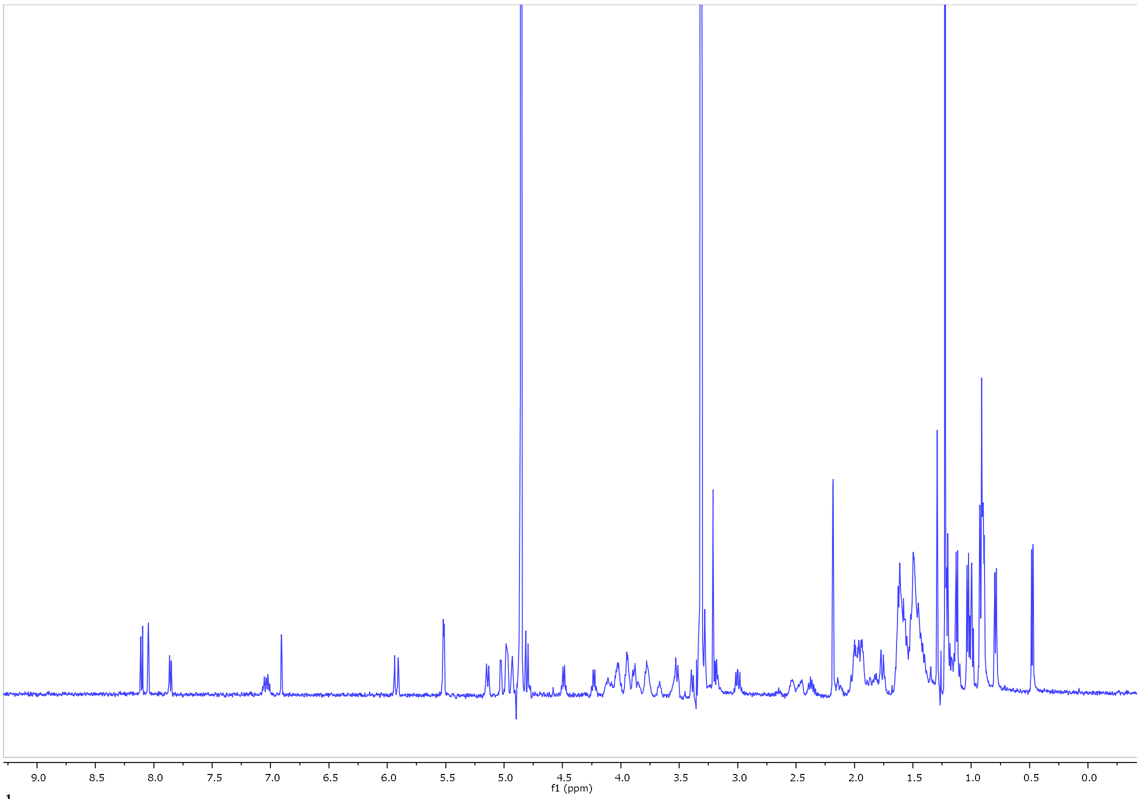

^{1}H-NMR (CD_3OD, 500 MHZ) of compound **6**

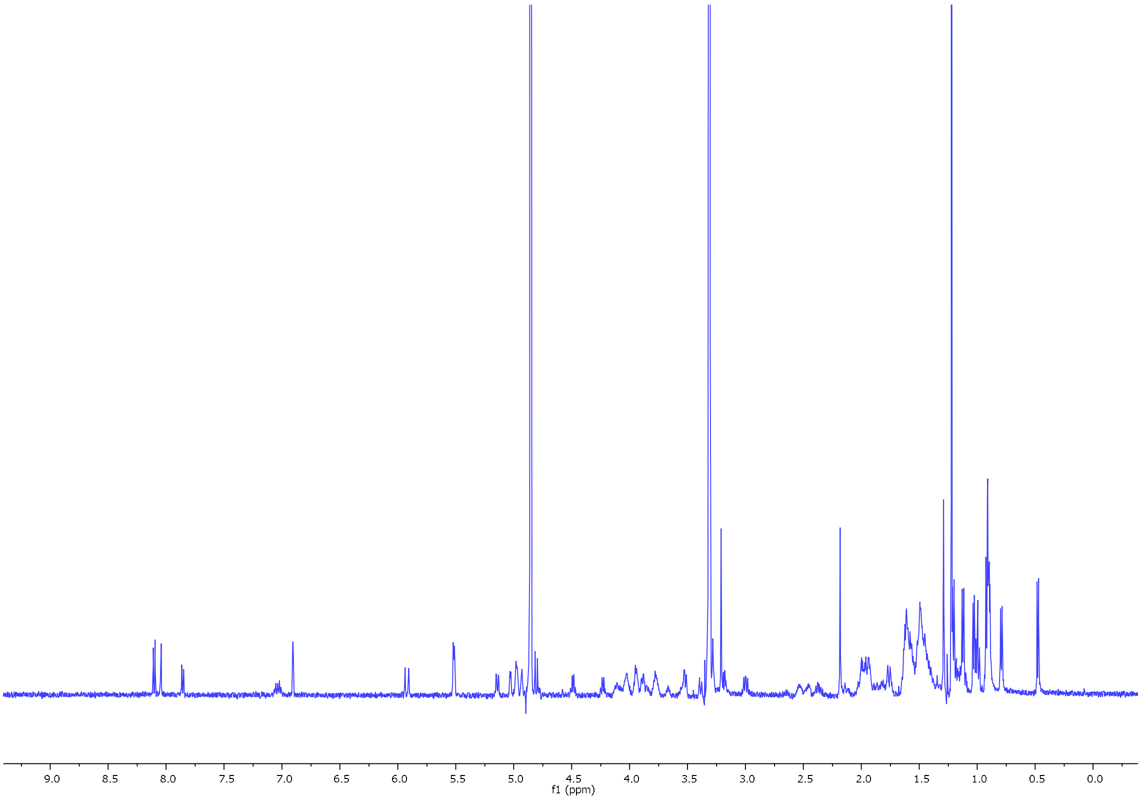

^{1}H spectrum of compound **6**

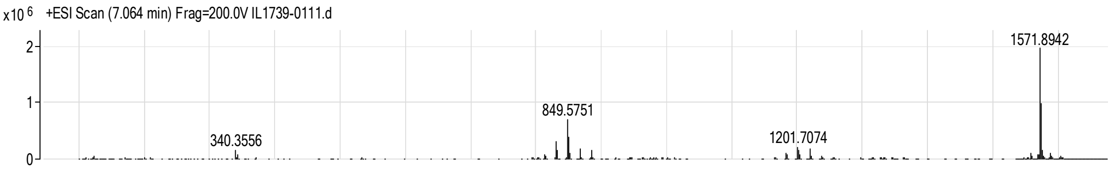

MS spectrum of compound **6**
