# Supplementary material for: Characterization and engineering of the biosynthesis gene cluster for antitumor macrolides PM100117 and PM100118 from a marine actinobacteria: generation of a novel improved derivative
Source: Microb Cell Fact. 2016 Feb 22;15:44. doi: 10.1186/s12934-016-0443-5 (PMC4763440; doi:10.1186/s12934-016-0443-5)
Supplement: Supplementary file 3 — 10.1186/s12934-016-0443-5 Plasmids construction. Table S1. Primers used in this work. Format: PDF. [file 12934_2016_443_MOESM3_ESM.pdf]

Methods S1. Plasmid construction.

pOJ-*gonP1* and pOJ-*gonP8*

An internal fragment of genes *gonP1* and *gonP8* was amplified with the primer pairs EcoRI-*P1*/HindIII-*P1* and EcoRI-*P8*/HindIII-*P8* (Table S1), respectively. The resulting PCR products were digested with EcoRI/HindIII and cloned into the same sites of plasmid pOJ260 to yield the gene disruption plasmids pOJ-*gonP1* and pOJ-*gonP8*. The correct insertion of the *gonP1* (3,504 bp) and *gonP8* (2,238 bp) gene fragments into pOJ260 was confirmed by sequencing with primer M13r. Plasmids pOJ-*gonP1* and pOJ-*gonP8* were transferred to *Streptomyces caniferus* GUA-06-05-006A by intergeneric conjugation to achieve *gonP1* and *gonP8* inactivation. Gene disruption in the resulting strains, *gonP1*$^{-}$ and *gonP8*$^{-}$, was verified by PCR with the primer pairs cf*P1*/M13r and cf*P8*/M13r, respectively. Primers cf*P1* and cf*P8* anneal to sequences outside the internal fragment used for gene inactivation and primer M13r anneals to the pOJ260 backbone.

pD-*gonM4*

The upstream (UP-*M4*) and downstream (DW-*M4*) sequences flanking gene *gonM4* were amplified with the primer pairs SpeI-*M4*/NsiI-*M4* and NdeI-*M4*/EcoRV-*M4* (Table S1), respectively. DW-*M4* (2,367 pb) and plasmid pEFBA-oriT were digested with NdeI/EcoRV and ligated to generate plasmid p*M4*-NE. This plasmid and fragment UP-*M4* (2,444 pb) were then digested with SpeI/NsiI and ligated to afford plasmid p*M4*-NESN. Insertion of UP-*M4* and DW-*M4* at both sides of the *aac(3)IV* gene in p*M4*-NESN was verified by PCR and sequencing with primers SpeI-*M4*, NsiI-*M4,* NdeI-*M4* and EcoRV-*M4*. A 1.6-kbp fragment containing the hygromycin B resistance (Hyg^{R}) gene marker, *hyg*, was extracted from pLHyg by SpeI/NheI digestion and cloned into the XbaI site of p*M4*-NESN to yield the gene-replacement plasmid pD-*gonM4*, which was transferred to *Streptomyces caniferus* GUA-06-05-006A by intergeneric conjugation to achieve *gonM4* deletion. Replacement of *gonM4* in the resulting Hyg^{s} Amp^{R} strain, Δ*gonM4*, was verified by PCR with primers cf*M4* (outside the deletion cassette) and Apra60 (internal to the Apm^{R} gene marker).

pD-*gonMT*

The upstream (UP-*MT*) and downstream (DW-*MT*) sequences flanking gene *gonMT* were amplified with the primer pairs SpeI-*MT*/NsiI-M*T* and BglII-*MT*/EcoRV-*MT* (Table S1), respectively. DW-*MT* (2,273 bp) and plasmid pEFBA-oriT were digested with BglII/EcoRV and BamHI/EcoRV, respectively, and ligated to generate plasmid p*MT*-BE. This plasmid and fragment UP-*MT* (2,294 bp) were then digested with SpeI/NsiI and ligated to afford plasmid p*MT*-BESN. Insertion of UP-*MT* and DW-*MT* at both sides of the *aac(3)IV* gene in p*MT*-BESN was verified by PCR and sequencing with primers SpeI-*MT*, NsiI-M*T*, BglII-*MT* and EcoRV-*MT*. A 1.6-kbp fragment containing the hygromycin B resistance (Hyg^R) gene marker, *hyg*, was extracted from pLHyg by SpeI/NheI digestion and cloned into the XbaI site of p*MT*-BESN to produce the gene replacement plasmid pD-*gonMT*, which was transferred to *Streptomyces caniferus* GUA-06-05-006A by intergeneric conjugation to achieve *gonMT* deletion. Replacement of *gonMT* in the resulting Hyg^s Amp^R strain, Δ*gonMT*, was verified by PCR with primers cf*MT* (outside the deletion cassette) and ApraII (internal to the Apm^R gene marker).

pD-*gonSL*

The upstream (UP-*SL*) and downstream (DW-*SL*) sequences flanking gene *gonSL* were amplified with the primer pairs SpeI-*SL*/NsiI-*SL* and NdeI-*SL*/EcoRV-*SL* (Table S1), respectively. UP-*SL* (2,138 bp) and plasmid pEFBA-oriT were digested with SpeI /NsiI and ligated to generate plasmid p*SL*-SN. This plasmid and fragment DW-*SL* were digested with NdeI/EcoRV. Digestion of DW-*SL* with NdeI generated two fragments, of 1.945 and 211 bp, respectively, the 211-bp fragment was cloned into p*SL*-SN (NdeI/EcoRV sites) to yield plasmid p*SL*-SN-200, which was then digested with NdeI and ligated to the 1.945-bp fragment to produce plasmid p*SL*-SNNE. The correct cloning orientation of the 1.945-bp DW-*SL* fragment was confirmed by PCR with primers NdeI-*SL* and EcoRV-*SL*. In addition, insertion of UP-*SL* and DW-*SL* at both sides of the *aac(3)IV* gene in p*SL*-SNNE was verified by sequencing with primers NdeI-*SL*, EcoRV-*SL*, SpeI-*SL* and NsiI-*SL*. A 1.6-kbp fragment containing the hygromycin B resistance (Hyg^R) gene marker, *hyg*, was then extracted from pLHyg by SpeI/NheI digestion and cloned into the XbaI site of p*SL*-NESN to yield the gene replacement plasmid pD-*gonSL*, which was transferred to *Streptomyces*

caniferus GUA-06-05-006A by intergeneric conjugation to achieve *gonSL* deletion. Replacement of *gonSL* in the resulting Hyg^{s} Amp^{R} strain, Δ*gonSL*, was verified by PCR with primers cf*SL* (outside the deletion cassette) and Apra60 (internal to the Apm^{R} gene marker).

pD-*gonS1*

The upstream (UP-*S1*) and downstream (DW-*S1*) sequences flanking gene *gonS1* were amplified with the primer pairs fNsiI-*S1*/rNsiI-*S1* and BglII-*S1*/EcoRV-*S1* (Table S1), respectively. DW-*S1* (2,131 bp) and plasmid pEFBA-oriT were digested with BglII/EcoRV and BamHI/EcoRV, respectively, and ligated to generate plasmid p*S1*-BE. This plasmid and fragment UP-*S1* (2.096 bp) were then digested with NsiI and ligated to afford plasmid p*S1*-BENN. Correct cloning orientation of the *gonS1* upstream fragment was confirmed by PCR with primers fNsiI-*S1*and apra60. In addition, insertion of UP-*S1* and DW-*S1* at both sides of the *aac(3)IV* gene in p*S1*-BENN was verified by sequencing with primers fNsiI-*S1*, rNsiI-*S1*, BglII-*S1* and EcoRV-*S1*. A 1.6-kbp fragment containing the hygromycin B resistance (Hyg^{R}) gene marker, *hyg*, was extracted from pLHyg by SpeI/NheI digestion and cloned into the XbaI site of p*S1*-BENN to yield the gene replacement plasmid pD-*gonS1*, which was transferred to *Streptomyces caniferus* GUA-06-05-006A by intergeneric conjugation to achieve *gonS1* deletion. Replacement of *gonS1* in the resulting Hyg^{s} Amp^{R} strain, Δ*gonS1*, was verified by PCR with primers cf*S1* (outside the deletion cassette) and Apra60 (internal to the Apm^{R} gene marker).

pD-*gonS2*

The upstream (UP-*S2*) and downstream (DW-*S2*) sequences flanking gene *gonS2* were amplified with the primer pairs NsiI-*S2*/SpeI-*S2* and fNdeI-*S2*/rNdeI-*S2* (Table S1), respectively. DW-*S2* (2,434 bp) and plasmid pEFBA-oriT were digested with NdeI and ligated to generate plasmid p*S2*-NN. Insertion of the *gonS2* downstream fragment in the correct orientation was confirmed by PCR with fNdeI-*S2* and apraII. This plasmid and UP-*S2* (2,208 bp) were then digested with NsiI/speI and ligated to afford plasmid p*S2*-NNNS. Insertion of UP-*S2* and DW-*S2* at both sides of the *aac(3)IV* gene in p*S2*-NNNS was verified by sequencing with primers NsiI-*S2*, SpeI-*S2*, fNdeI-*S2* and rNdeI-*S2*. A 1.6-kbp

fragment containing the hygromycin B resistance (Hyg^R) gene marker, *hyg*, was extracted from pLHyg by SpeI/NheI digestion and cloned into the XbaI site of p*S1*-BENN to yield the gene replacement plasmid pD-*gonS2*, which was transferred to *Streptomyces caniferus* GUA-06-05-006A by intergeneric conjugation to achieve *gonS2* deletion. Replacement of *gonS2* in the resulting Hyg^s Amp^R strain, Δ*gonS2*, was verified by PCR with primers cf*S2* (outside the deletion cassette) and ApraII (internal to the Apm^R gene marker).

pD-*gonCP*

The upstream (UP-*CP*) and downstream (DW-*CP*) sequences flanking gene *gonCP* were amplified with the primer pairs SpeI-*CP*/NsiI-*CP* and BamHI-*CP*/EcoRV-*CP* (Table S1), respectively. DW-*CP* (2,548 bp) and plasmid pEFBA-oriT were digested with BamHI/EcoRV and ligated to generate plasmid p*CP*-BE. This plasmid and UP-*CP* (2,100 bp) were then digested with SpeI/NsiI and ligated to afford plasmid p*CP*-BESN. Insertion of UP-*CP* and DW-*CP* at both sides of the *aac(3)IV* gene in p*CP*-BESN was verified by PCR and sequencing with primers SpeI-*CP*, NsiI-CP, BamHI-*CP* and EcoRV-*CP*. A 1.6-kbp fragment containing the hygromycin B resistance (Hyg^R) gene marker, *hyg*, was extracted from pLHyg by SpeI/NheI digestion and cloned into the XbaI site of p*CP*-BESN to yield the gene replacement plasmid pD-*gonCP*, which was transferred to *Streptomyces caniferus* GUA-06-05-006A by intergeneric conjugation to achieve *gonCP* deletion. Replacement of *gonLCP* in the resulting Hyg^s Amp^R strain, Δ*gonCP*, was verified by PCR with primers cf*CP* (outside the deletion cassette) and Apra60 (internal to the Apm^R gene marker).

pD-*gonMR*

The upstream (UP-*MR*) and downstream (DW-*MR*) sequences flanking gene *gonMR* were amplified with the primer pairs SpeI-*MR*/NsiI-M*R* and BglII-*MR*/EcoRV-*MR* (Table S1), respectively. DW-*MR* (2,393 bp) and plasmid pEFBA-oriT were digested with BglII/EcoRV and BamHI/EcoRV, respectively, and ligated to generate plasmid p*MR*-BE. This plasmid and UP-*MR* (2,571 bp) were then digested with SpeI/NsiI and ligated to yield plasmid p*MR*-BESN. Insertion of UP-*MR* and DW-*MR* at both sides of the *aac(3)IV* gene in p*MR*-BESN was verified by PCR and sequencing with primers SpeI-*MR*, NsiI-M*R,*

BglII-*MR* and EcoRV-*MR*. A 1.6-kbp fragment containing the hygromycin B resistance (Hyg^R) gene marker, *hyg*, was extracted from pLHyg by SpeI/NheI digestion and cloned into the XbaI site of p*MR*-BESN to yield the gene replacement plasmid pD-*gonMR*, which was transferred to *Streptomyces caniferus* GUA-06-05-006A by intergeneric conjugation to achieve *gonMR* deletion. Replacement of *gonMR* in the resulting Hyg^s Amp^R strain, Δ*gonMR*, was verified by PCR with primers cf*MR* (outside the deletion cassette) and ApraII (internal to the Apm^R gene marker).

pD-*gonL1*

The upstream (UP-*L1*) and downstream (DW-*L1*) sequences flanking gene *gonL1* were amplified with the primer pairs SpeI-*L1*/NsiI-*L1* and BglII-*L1*/EcoRV-*L1* (Table S1), respectively. DW-L1 (2,500 bp) and plasmid pEFBA-oriT were digested with BglII/EcoRV and BamHI/EcoRV, respectively, and ligated to generate plasmid p*L1*-BE. This plasmid and UP-*L1* (2,732 bp) were then digested with SpeI/NsiI and ligated to afford plasmid p*L1*-BESN. Insertion of UP-*L1* and DW-*L1* at both sides of the *aac(3)IV* gene in p*L1*-BESN was verified by PCR and sequencing with primers SpeI-*L1*, NsiI-*L1,* BglII-*L1* and EcoRV-*L1*. A 1.6-kbp fragment containing the hygromycin B resistance (Hyg^R) gene marker, *hyg*, was extracted from pLHyg by SpeI/NheI digestion and cloned into the XbaI site of p*L1*-BESN to yield the gene replacement plasmid pD-*gonL1*, which was transferred to *Streptomyces caniferus* GUA-06-05-006A by intergeneric conjugation to achieve *gonL1* deletion. Replacement of *gonL1* in the resulting Hyg^s Amp^R strain, Δ*gonL1,* was verified by PCR with primers cf*L1* (outside the deletion cassette) and ApraII (internal to the Apm^R gene marker).

pD-*orf9*

The upstream (UP-*orf9*) and downstream (DW-*orf9*) sequences flanking gene *orf9* were amplified with the primer pairs SpeI-*orf9*/NsiI-*orf9* and BglII-*orf9*/EcoRV-*orf9* (Table S1), respectively. DW-*orf9* (2,434 bp) and plasmid pEFBA-oriT were digested with BglII/EcoRV and BamHI/EcoRV, respectively, and ligated to generate plasmid pG*orf9*-BE. This plasmid and UP-*orf9* (2,496 bp) were then digested with SpeI/NsiI and ligated to afford plasmid pG*orf9*-BESN. Insertion of UP-*orf9* and DW-*orf9* at both sides of the

aac(3)IV gene in pG*orf9*-BESN was verified by PCR and sequencing with primers SpeI-*orf9*, NsiI-*orf9*, BglII-*orf9* and EcoRV-*orf9*. A 1.6-kbp fragment containing the hygromycin B resistance (Hyg^R) gene marker, *hyg*, was extracted from pLHyg by SpeI/NheI digestion and cloned into the XbaI site of pG*orf10*-BESN to produce the gene replacement plasmid pD-*orf9*, which was transferred to *Streptomyces caniferus* GUA-06-05-006A by intergeneric conjugation to achieve *orf9* deletion. Replacement of *orf9* in the resulting Hyg^s Amp^R strain, Δ5201, was verified by PCR with primers cf09 (outside the deletion cassette) and Apra60 (internal to the Apm^R gene marker).

pD-*orf10*

The upstream (UP-*orf10*) and downstream (DW-*orf10*) sequences flanking gene *orf10* were amplified with the primer pairs SpeI-*orf10*/NsiI-*orf10* and BglII-*orf10*/EcoRV-*orf10* (Table S1), respectively. DW-*orf10* (2,380 bp) and plasmid pEFBA-oriT were digested with BglII/EcoRV and BamHI/EcoRV, respectively, and ligated to generate plasmid pG*orf10*-BE. This plasmid and UP-*orf10* (2,667 bp) were then digested with SpeI/NsiI and ligated to afford plasmid pG*orf10*-BESN. Insertion of UP-*orf10* and DW-*orf10* at both sides of the *aac(3)IV* gene in pG*orf10*-BESN was verified by PCR and sequencing with primers SpeI-*orf10*, NsiI-*orf10*, BglII-*orf10* and EcoRV-*orf10*. A 1.6-kbp fragment containing the hygromycin B resistance (Hyg^R) gene marker, *hyg*, was extracted from pLHyg by SpeI/NheI digestion and cloned into the XbaI site of pG*orf10*-BESN to produce the gene replacement plasmid pD-*orf10*, which was transferred to *Streptomyces caniferus* GUA-06-05-006A by intergeneric conjugation to achieve *orf10* deletion. Replacement of *orf10* in the resulting Hyg^s Amp^R strain, Δ5257, was verified by PCR with primers cf10 (outside the deletion cassette) and ApraII (internal to the Apm^R gene marker).

pD-*orf11*

The upstream (UP-*orf11*) and downstream (DW-*orf11*) sequences flanking gene *orf11* were amplified with the primer pairs SpeI-*orf11*/NsiI-*orf11* and NdeI-*orf11*/EcoRV-*orf11* (Table S1), respectively. UP-*orf11* (2,195 bp) and plasmid pEFBA-oriT were digested with SpeI/NsiI and ligated to generate plasmid pG*orf11*-SN. This plasmid and DW-*orf11* (2,136 bp) were then digested with NdeI/EcoRV and ligated to afford plasmid pG*orf11*-SNNE.

Insertion of UP-*orf11* and DW-*orf11* at both sides of the *aac(3)IV* gene in pG*orf11*-SNNE was verified by PCR and sequencing with primers SpeI-*orf11*, NsiI-*orf11*, NdeI-*orf11* and EcoRV-*orf11*. A 1.6-kbp fragment containing the hygromycin B resistance (Hyg^R) gene marker, *hyg*, was extracted from pLHyg by SpeI/NheI digestion and cloned into the XbaI site of pG*orf11*-SNNE to produce the gene replacement plasmid pD-*orf11*, which was transferred to *Streptomyces caniferus* GUA-06-05-006A by intergeneric conjugation to achieve *orf11* deletion. Replacement of *orf11* in the resulting Hyg^s Amp^R strain, Δ5259, was verified by PCR with primers cf11 (outside the deletion cassette) and ApraII (internal to the Apm^R gene marker).

pD-*orf13*

The upstream (UP-*orf13*) and downstream (DW-*orf13*) sequences flanking gene *orf13* were amplified with the primer pairs SpeI-*orf13*/PstII-*orf13* and BamHI-*orf13*/EcoRV-*orf13* (Table S1), respectively. DW-*orf13* (2,347 bp) and plasmid pEFBA-oriT were digested with BamHI/EcoRV and ligated to generate plasmid pG*orf13*-BE. This plasmid and UP-*orf13* (2,360 bp) were then digested with SpeI/PstI and ligated to afford plasmid pG*orf13*-BESP. Insertion of DW-*orf13* and UP-*orf13* at both sides of the *aac(3)IV* gene in pG*orf13*-BESP was verified by PCR and sequencing with primers SpeI-*orf13*, PstII-*orf13*, BamHI-*orf13* and EcoRV-*orf13*. A 1.6-kbp fragment containing the hygromycin B resistance (Hyg^R) gene marker, *hyg*, was extracted from pLHyg by SpeI/NheI digestion and cloned into the XbaI site of pG*orf13*-BESP to produce the gene replacement plasmid pD-*orf13*, which was transferred to *Streptomyces caniferus* GUA-06-05-006A by intergeneric conjugation to achieve *orf13* deletion. Replacement of *orf13* in the resulting Hyg^s Amp^R strain, Δ5261, was verified by PCR with primers cf13 (outside the deletion cassette) and Apra60 (internal to the Apm^R gene marker).

pC-*gonP8*

A DNA fragment of 7,326 bp containing the entire ORF of *gonP8* (nt -20 to +7,306 from start codon) was amplified with the primer pair cp*P8*-Xb/cp*P8*-ERV (Table S1). The resulting PCR fragment was digested with XbaI/EcoRV and cloned into the same sites of plasmid pSETHe (see methods in the article), under the control of the *ermE*p* promoter, to

yield the complementation plasmid pC-*gonP8*. Insertion of *gonP8* into pSETHe was confirmed by PCR and sequencing with primers cp*P8*-Xb and cp*P8*-ERV. Plasmid pC-*gonP8* was then transferred to the mutant strain *gonP8*⁻ by intergeneric conjugation to produce the strain CP*gonP8* in which *gonP8* inactivation was complemented.

pC-*gonM4*

A DNA fragment of 998 bp containing the entire ORF of *gonM4* (nt -26 to +972 from start codon) was amplified with the primer pair cp*M4*-Xb/cp*M4*-ERV (Table S1). The resulting PCR fragment was digested with XbaI/EcoRV and cloned into the same sites of plasmid pSETHe (see methods in the article), under the control of the *ermE*p* promoter, to yield the complementation plasmid pC-*gonM4*. Insertion of *gonM4* into pSETHe was confirmed by PCR and sequencing with primers cp*M4*-Xb and cp*M4*-ERV. Plasmid pC-*gonM4* was transferred to the mutant strain Δ*gonM4* by intergeneric conjugation to produce the strain CP*gonM4* in which *gonM4* deletion was complemented.

pC-*gonMT*

A DNA fragment of 1,211 bp containing the entire ORF of *gonMT* (nt -65 to +1,146 from start codon) was amplified with the primer pair cp*MT*-Xb/cp*MT*-ERV (Table S1). The resulting PCR fragment was digested with XbaI/EcoRV and cloned into the same sites of plasmid pSETHe (see methods in the article), under the control of the *ermE*p* promoter, to yield the complementation plasmid pC-*gonMT*. Insertion of *gonMT* into pSETHe was confirmed by PCR and sequencing with primers cp*MT*-Xb and cp*MT*-ERV. Plasmid pC-*gonMT* was transferred to the mutant strain Δ*gonMT* by intergeneric conjugation to produce the strain CP*gonMT* in which *gonMT* deletion was complemented.

pC-*gonSL*

A DNA fragment of 2,514 bp containing the entire ORF of *gonSL* (nt -23 to +2,491 from start codon) was amplified with the primer pair cp*SL*-Xb/cp*SL*-ERV (Table S1). The resulting PCR fragment was digested with XbaI/EcoRV and cloned into the same sites of plasmid pSETHe (see methods in the article), under the control of the *ermE*p* promoter, to yield the complementation plasmid pC-*gonSL*. Insertion of *gonSL* into pSETHe was

confirmed by PCR and sequencing with primers cp*SL*-Xb and cp*SL*-ERV. Plasmid pC-*gonSL* was transferred to the mutant strain Δ*gonSL* by intergeneric conjugation to produce the strain CP*gonSL* in which *gonSL* deletion was complemented.

pC-*gonS1*

A DNA fragment of 1,135 bp containing the entire ORF of *gonS1* (nt -24 to +1,111 from start codon) was amplified with the primer pair cp*S1*-Xb/cp*S1*-ERV (Table S1). The resulting PCR fragment was digested with XbaI/EcoRV and cloned into the same sites of plasmid pSETHe (see methods in the article), under the control of the *ermE*p* promoter, to yield the complementation plasmid pC-*gonS1*. Insertion of *gonS1* into pSETHe was confirmed by PCR and sequencing with primers cp*S1*-Xb and cp*S1*-ERV. Plasmid pC-*gonS1* was transferred to the mutant strain Δ*gonS1* by intergeneric conjugation to produce the strain CP*gonS1* in which *gonS1* deletion was complemented.

pC-*gonS2*

A DNA fragment of 1,125 bp containing the entire ORF of *gonS2* (nt -23 to +1,102 from start codon) was amplified with the primer pair cp*S2*-Xb/cp*S2*-ERV (Table S1). The resulting PCR fragment was digested with XbaI/EcoRV and cloned into the same sites of plasmid pSETHe (see methods in the article), under the control of the *ermE*p* promoter, to yield the complementation plasmid pC-*gonS2*. Insertion of *gonS2* into pSETHe was confirmed by PCR and sequencing with primers cp*S2*-Xb and cp*S2*-ERV. Plasmid pC-*gonS2* was transferred to the mutant strain Δ*gonS2* by intergeneric conjugation to produce the strain CP*gonS2* in which *gonS2* deletion was complemented.

pC-*gonCP*

A DNA fragment of 1,361 bp containing the entire ORF of *gonCP* (nt -31 to +1,330 from start codon) was amplified with the primer pair cp*CP*-Xb/cp*CP*-ERV (Table S1). The resulting PCR fragment was digested with XbaI/EcoRV and cloned into the same sites of plasmid pSETHe (see methods in the article), under the control of the *ermE*p* promoter, to yield the complementation plasmid pC-*gonCP*. Insertion of *gonCP* into pSETHe was confirmed by PCR and sequencing with primers cp*CP*-Xb and cp*CP*-ERV. Plasmid pC-

gonCP was transferred to the mutant strain Δ*gonCP* by intergeneric conjugation to produce the strain CP*gonCP* in which *gonCP* deletion was complemented.

pC-*gonMR*

A DNA fragment of 600 bp containing the entire ORF of *gonMR* (nt -30 to +570 from start codon) was amplified with the primer pair cp*MR*-Xb/cp*MR*-ERV (Table S1). The resulting PCR fragment was digested with XbaI/EcoRV and cloned into the same sites of plasmid pSETHe (see methods in the article), under the control of the *ermE*p* promoter, to yield the complementation plasmid pC-*gonMR*. Insertion of *gonMR* into pSETHe was confirmed by PCR and sequencing with primers cp*MR*-Xb and cp*MR*-ERV. Plasmid pC-*gonMR* was transferred to the mutant strain Δ*gonMR* by intergeneric conjugation to produce the strain CP*gonMR* in which *gonMR* deletion was complemented.

pC-*gonL1*

A DNA fragment of 3,005 bp containing the entire ORF of *gonL1* (nt -24 to +2,981 from start codon) was amplified with the primer pair cp*L1*-Xb/cp*L1*-ERV (Table S1). The resulting PCR fragment was digested with XbaI/EcoRV and cloned into the same sites of plasmid pSETHe (see methods in the article), under the control of the *ermE*p* promoter, to yield the complementation plasmid pC-*gonL1*. Insertion of *gonL1* into pSETHe was confirmed by PCR and sequencing with primers cp*L1*-Xb and cp*L1*-ERV. Plasmid pC-*gonL1* was transferred to the mutant strain Δ*gonL1* by intergeneric conjugation to produce the strain CP*gonL1* in which *gonL1* deletion was complemented.

Table S1. Primers used in this work

Primer	Sequence (5'-3')	PCR product and purpose
EcoRI-*P1*	TATAGAATTCAGTCCCTGAACGAGGTGCT	internal gene fragment for *gonP1* inactivation
HindIII-*P1*	TATAAAGCTTGGGTCAACTTCCTTAGTTGTGC	
EcoRI-*P8*	TATAGAATTCTGTAGTCCCAGGAAGTAGGACAA	internal gene fragment for *gonP8* inactivation
HindIII-*P8*	TATAAAGCTTGGAGATGGTGGAGAGTTACGTG	
SpeI-*M4*	TATAACTAGTGACCATGTCGTCTCCCACTT	upstream flanking region for *gonM4* deletion
NsiI-*M4*	TATAATGCATTACTCGTCCAGCACATACGG	
NdeI-*M4*	TATACATATGCACATCGGGCTGTATGTGAA	downstream flanking region for *gonM4* deletion
EcoRV-*M4*	TATAGATATCGTTCCCCGTGGAGGACTACT	
SpeI-*MT*	TATAACTAGTGGTGTGTGACGGTGAAGATG	upstream flanking region for *gonMT* deletion
NsiI-*MT*	TATAATGCATCCATGAAGAGGAGGAAGTGG	
BglII-*MT*	TATAAGATCTGGTGTACACGCCGAAAGAGT	downstream flanking region for *gonMT* deletion
EcoRV-*MT*	TATAGATATCGTTCGGAATCTGTTGCTGGT	
SpeI-*SL*	TATAACTAGTCTCATGGAGGCGCTGTACTT	upstream flanking region for *gonSL* deletion
NsiI-*SL*	TATAATGCATGGAGGAGCAACTCGTCCAG	
NdeI-*SL*	TATACATATGTGCAGCACCTCAAGAGAGAA	downstream flanking region for *gonSL* deletion
EcoRV-*SL*	TATAGATATCACTGCACCATGACCAGTGAC	
fNsiI-*S1*	TATAATGCATGATAGCCCTGTCCGAGGAAC	upstream flanking region for *gonS1* deletion
rNsiI-*S1*	TATAATGCATCCATTCCGGAAGAAACACTC	
BglII-*S1*	TATAAGATCTGTCTTCATTTCCCTGGAGCA	downstream flanking region for *gonS1* deletion
ERV-*S1*	TATAGATATCTCCGAGATTCCAGTATGTTCG	
fNdeI-*S2*	TATACATATGACCTCCTCTTCCATGACACG	downstream flanking region for *gonS2* deletion
rNdeI-*S2*	TATACATATGTCCTGGACGGTATGACACAA	
NsiI-*S2*	TATAATGCATGACTCCCAGCGAGTTGATGT	upstream flanking region for *gonS2* deletion
SpeI-*S2*	TATAACTAGTCCGAAGAAGTGCGGTACG	

EcoRV-*CP*	TATAGATATCGCACAGACCTTCTCCTCCAG	downstream flanking region for *gonCP* deletion
BamHI-*CP*	TATAGGATCCTACGAACTGCCTGTGACCTG	
NsiI-*CP*	TATAATGCATGTCGGGACAGGTCCGTACTA	upstream flanking region for *gonCP* deletion
SpeI-*CP*	TATAACTAGTGTGGATGATCTCCGTCGAGT	
EcoRV-*MR*	TATAGATATCCGATGAAGAACCCGTTCAC	downstream flanking region for *gonMR* deletion
BglII-*MR*	TATAAGATCTGATTGCTGTCGAAGCTGGAG	
NsiI-*MR*	TATAATGCATCTCCAGCGGTGAGGTGTC	upstream flanking region for *gonMR* deletion
SpeI-*MR*	TATAACTAGTATCCTGCTGGTGCTGTCC	
EcoRV-*L1*	TATAGATATCGCACCCTCGTCTACTGCTG	downstream flanking region for *gonL1* deletion
BglII-*L1*	TATAAGATCTTCGAGCAACATCTGACATCC	
NsiI-*L1*	TATAATGCATAACGGGAAGTCATGTTCCAG	upstream flanking region for *gonL1* deletion
SpeI-*L1*	TATAACTAGTGTCTTCACCCTGTCGTCGAT	
SpeI-*orf9*	TATAACTAGTACCAACTGGGCATGCTGAC	upstream flanking region for *orf9* deletion
NsiI-*orf9*	TATAATGCATGTGGCTTCACGGGACAGG	
BglII-*orf9*	TATAAGATCTCCAGCAGGAGAACCTCTACC	downstream flanking region for *orf9* deletion
EcoRV-*orf9*	TATAGATATCGGATCTGCTACGGCACTGTC	
EcoRV-*orf10*	TATAGATATCGTTGTGGAGATCGGCTTCTC	downstream flanking region for *orf10* deletion
BglII-*orf10*	TATAAGATCTACGAGGTCGTACCTCAGCAG	
NsiI-*orf10*	TATAATGCATACCTTGTCCGTCAGGTTCAG	upstream flanking region for *orf10* deletion
SpeI-*orf10*	TATAACTAGTTATCGAGATTCCTGGCCAAC	
EcoRV-*orf11*	TATAGATATCGGCTCGTACTGCTGGATCTT	downstream flanking region for *orf11* deletion
NdeI-*orf11*	TATACATATGGACTGGACGCACCAGTACCT	
NsiI-*orf11*	TATAATGCATGTCGTGTATGCCGCTGAACT	upstream flanking region for *orf11* deletion
SpeI-*orf11*	TATAACTAGTACGGACTGTTTTGGTCTCGT	
SpeI-*orf13*	TATAACTAGTACCTTGTCCGTCAGGTTCAG	upstream flanking region for *orf13* deletion
PstI-*orf13*	TATACTGCAGCTCAATACGGCCCACACATT	
BamHI-*orf13*	TATAGGATCCTCGCTGATGAAGTGTTGAGC	downstream flanking region for *orf13* deletion
EcoRV-*orf13*	TATAGATATCTTCGAGGACAACCTGAGAGG	

cp*P8*-ERV	TATAGATATCGACTCCCAGCGAGTTGATGT	*gonP8*$^{-}$ complementation
cp*P8*-Xb	TATATCTAGAGTGATTGGAATCCGAGAAGC	
cp*M4*-Xb	TATATCTAGAAGCTCGACGAGCCCCGGAG	Δ*gonM4* complementation
cp*M4*-ERV	TATAGATATCCATGATGGAACTGGTCGATG	
cp*M*T-Xb	TATATCTAGACCACTTCCTCCTCTTCATGG	Δ*gonMT* complementation
cp*MT*-ERV	TATAGATATCTCCTGGACGGTATGACACAA	
cp*SL*-Xb	TATATCTAGAAGCCCCTGAGAGTGAGTGC	Δ*gonSL* complementation
cp*SL*-ERV	TATAGATATCGCAGCGTCGCCAGCAGCGCGT	
cp*S1*-Xb	TATATCTAGACCACACGGAATGAGGAGATT	Δ*gonS1* complementation
cp*S1*-ERV	TATAGATATCATCTTCACCGTCACACACCA	
cp*S2*-ERV	TATAGATATCCTGATCCCTGTTGCAGGTC	Δ*gonS2* complementation
cp*S2*-Xb	TATATCTAGAGGACAGAACGGAGACAATCC	
cp*CP*-ERV	TATAGATATCCCAATGGAATTCCTCGGATA	Δ*gonCP* complementation
cp*CP*-Xb	TATATCTAGACCCAGTGGTCACCTACAGCA	
cp*MR*-ERV	TATAGATATCCTGACCGGCGGCGGGTTCCG	Δ*gonMR* complementation
cp*MR*-Xb	TATATCTAGACCGGGAGGGCAGTTCGGGGAG	
cp*L1*-ERV	TATAGATATCCGATCACCCGGGATAGTTTA	Δ*gonL1* complementation
cp*L1*-Xb	TATATCTAGAAGGTGCGTGCAGAGGAGACT	
cf*M4*	CTCGGACCGTATGGAACTGT	used with Apra60 *for* Δ*gonM4* confirmation
cf*MT*	GACACCCCGATGAACGACT	used with ApraII *for* Δ*gonMT* confirmation
cf*SL*	TCTTCCGTACGTGTGTGGAC	used with Apra60 *for* Δ*gonSL* confirmation
cf*S1*	GCGTGGATGCTCTACACCTC	used with Apra60 *for* Δ*gonS1* confirmation
cf*S2*	GGCCCAGGAGCACTTCAC	used with ApraII *for* Δ*gonS2* confirmation
cf*CP*	TCGACTTCGAGACCAATGTG	used with Apra60 *for* Δ*gonCP* confirmation
cf*MR*	CTCACCCTGCTCGTCTTCA	used with ApraII *for* Δ*gonMR* confirmation
cf*L1*	CACCGGCTACCTCTTCCTC	used with ApraII *for* Δ*gonL1* confirmation
cf09	CTTCATCCACGAGACCAAGG	used with Apra60 *for* Δ5201 confirmation
cf10	AGCACCAGATCACGTTCCTC	used with ApraII *for* Δ5257 confirmation

cf11	GAAGACCTTCACCAGGTCCA	used with ApraII *for* Δ5259 confirmation
cf13	CGACGTTGTTGACGAGTACG	used with Apra60 *for* Δ5261 confirmation
Apra60	CCAAGGTTGAGAAGCTGACC	reverse primer annealing to *aac(3)IV*
ApraII	CTTCAGGATGGCAAGTTGGT	forward primer annealing to *aac(3)IV*
cf*P1*	GAAGCGACCAGTCCGTAAAC	used with M13r for *gonP1*$^{-}$ confirmation
cf*P8*	GTGATTGGAATCCGAGAAGC	used with M13r for *gonP8*$^{-}$ confirmation
M13r	CAGGAAACAGCTATGAC	M13 reverse primer
